# Suboptimal gestational weight gain and neonatal outcomes in low and middle income countries: individual participant data meta-analysis

**DOI:** 10.1136/bmj-2022-072249

**Published:** 2023-09-21

**Authors:** Nandita Perumal, Dongqing Wang, Anne Marie Darling, Enju Liu, Molin Wang, Tahmeed Ahmed, Parul Christian, Kathryn G Dewey, Gilberto Kac, Stephen H Kennedy, Vishak Subramoney, Brittany Briggs, Wafaie W Fawzi, GWG Pooling Project Consortium, Ajibola Ibraheem Abioye, Manfred Accrombessi, Seth Adu-Afarwuah, Joao Guilherme Alves, Carla Adriane Leal de Araújo, Shams Arifeen, Rinaldo Artes, Per Ashorn, Ulla Ashorn, Omolola Olukemi Ayoola, Gabriela Chico-Barba, Robin Bernstein, Zulfiqar A Bhutta, Valérie Briand, Elvira Beatriz Calvo, Marly Augusto Cardoso, Yue Cheng, Peter Ellis Clayton, Shalean M Collins, John Kennedy Cruickshank, Delanjathan Devakumar, Christopher P Duggan, Pratibha Dwarkanath, Frankie J Fair, Henrik Friis, Alison D Gernand, Shibani Ghosh, Exnevia Gomo, Rebecca Grais, Ousmane Guindo, Guadalupe Estrada Gutierrez, K Michael Hambidge, Rezwanul Haque, Lieven Huybregts, Romaina Iqbal, Sheila Isanaka, Keith P West, José Roberto da Silva Junior, Maria Ome-Kaius, Joanne Katz, Subarna Khatry, Patrick Kolsteren, Nancy F Krebs, Teija Kulmala, Pratap Kumar, Anura V Kurpad, Carl Lachat, Anna Lartey, Qian Li, Jacqueline M Lauer, See Ling Loy, Nur Indrawaty Lipoeto, Laura Beatriz López, Abdullah Al Mahmud, G Arun Maiya, Kenneth Maleta, Maíra Barreto Malta, Dharma S Manandhar, Charles Mangani, Yves Martin-Prevel, Reynaldo Martorell, Susana L Matias, Elizabeth M McClure, Joshua D Miller, Marhazlina Mohamad, Hamid Jan Jan Mohamed, Sophie Moore, Paola S Mosquera, Malay Kanti Mridha, Ferdinand M Mugusi, Cinthya Muñoz-Manrique, Salifu Nanga, Barnabas K Natamba, Minyanga Nkhoma, David Osrin, Andrea B Pembe, Otilia Perichart-Perera, Ahmed Tijani Bawah, Eric Kwasi Ofori, Zul Premji, Andrew M Prentice, Juha Pyykkö, Preetha Ramachandra, Usha Ramakrishnan, Juan Rivera, Dominique Roberfroid, Ameyalli Rodríguez-Cano, Patricia Lima Rodrigues, Stephen J Rogerson, Hugo Martínez-Rojano, Patricia H C Rondó, Daniel E Roth, Reyna Sámano, Naomi M Saville, Saijuddin Shaikh, Bhim P Shrestha, Robin Shrestha, Hora Soltani, Sajid Soofi, Tinku Thomas, James Tielsch, Holger W Unger, Willy Urassa, Juliana dos Santos Vaz, Lee Wu, Nianhong Yang, Sera L Young, Lingxia Zeng, Chunrong Zhong, Zhonghai Zhu

**Affiliations:** 1 Department of Epidemiology and Biostatistics, University of South Carolina, Columbia, SC, USA; 2 Department of Global Health and Population, Harvard T H Chan School of Public Health, Harvard University, Boston, MA, USA; 3 Department of Global and Community Health, College of Health and Human Services, George Mason University, Fairfax, VA, USA; 4 Institutional Centers for Clinical and Translational Research, Boston Children’s Hospital, Boston, MA, USA; 5 Division of Gastroenterology, Hepatology and Nutrition, Boston Children’s Hospital, Harvard Medical School, Boston, MA, USA; 6 Department of Epidemiology, Harvard T H Chan School of Public Health, Harvard University, Boston, MA, USA; 7 Department of Biostatistics, Harvard T H Chan School of Public Health, Harvard University, Boston, MA, USA; 8 Nutrition & Clinical Services, International Centre for Diarrheal Disease Research, Dhaka, Bangladesh; 9 Department of International Health, Johns Hopkins Bloomberg School of Public Health, Baltimore, MD, USA; 10 Department of Nutrition, University of California, Davis, CA, USA; 11 Nutritional Epidemiology Observatory, Josué de Castro Nutrition Institute, Rio de Janeiro Federal University, Rio de Janeiro, Brazil; 12 Nuffield Department of Women's & Reproductive Health, University of Oxford, Oxford, UK; 13 DVPL Tech, Dubai, United Arab Emirates; 14 Certara USA, on behalf of the Bill & Melinda Gates Foundation, Seattle, WA, USA; 15 Department of Nutrition, Harvard T H Chan School of Public Health, Harvard University, Boston, MA, USA

## Abstract

**Objective:**

To estimate the associations between gestational weight gain (GWG) during pregnancy and neonatal outcomes in low and middle income countries.

**Design:**

Individual participant data meta-analysis.

**Setting:**

Prospective pregnancy studies from 24 low and middle income countries.

**Main outcome measures:**

Nine neonatal outcomes related to timing (preterm birth) and anthropometry (weight, length, and head circumference) at birth, stillbirths, and neonatal death.

**Analysis methods:**

A systematic search was conducted in PubMed, Embase, and Web of Science which identified 53 prospective pregnancy studies published after the year 2000 with data on GWG, timing and anthropometry at birth, and neonatal mortality. GWG adequacy was defined as the ratio of the observed maternal weight gain over the recommended weight gain based on the Institute of Medicine body mass index specific guidelines, which are derived from data in high income settings, and the INTERGROWTH-21st GWG standards. Study specific estimates, adjusted for confounders, were generated and then pooled using random effects meta-analysis models. Maternal age and body mass index before pregnancy were examined as potential modifiers of the associations between GWG adequacy and neonatal outcomes.

**Results:**

Overall, 55% of participants had severely inadequate (<70%) or moderately inadequate (70% to <90%) GWG, 22% had adequate GWG (90-125%), and 23% had excessive GWG (≥125%). Severely inadequate GWG was associated with a higher risk of low birthweight (adjusted relative risk 1.62, 95% confidence interval 1.51 to 1.72; 48 studies, 93 337 participants; τ^2^=0.006), small for gestational age (1.44, 1.36 to 1.54; 51 studies, 93 191 participants; τ^2^=0.016), short for gestational age (1.47, 1.29 to 1.69; 40 studies, 83 827 participants; τ^2^=0.074), and microcephaly (1.57, 1.31 to 1.88; 31 studies, 80 046 participants; τ^2^=0.145) compared with adequate GWG. Excessive GWG was associated with a higher risk of preterm birth (1.22, 1.13 to 1.31; 48 studies, 103 762 participants; τ^2^=0.008), large for gestational age (1.44, 1.33 to 1.57; 47 studies, 90 044 participants; τ^2^=0.009), and macrosomia (1.52, 1.33 to 1.73; 29 studies, 68 138 participants; τ^2^=0) compared with adequate GWG. The direction and magnitude of the associations between GWG adequacy and several neonatal outcomes were modified by maternal age and body mass index before pregnancy.

**Conclusions:**

Inadequate and excessive GWG are associated with a higher risk of adverse neonatal outcomes across settings. Interventions to promote optimal GWG during pregnancy are likely to reduce the burden of adverse neonatal outcomes, however further research is needed to assess optimal ranges of GWG based on data from low and middle income countries.

## Introduction

Optimal maternal nutrition during pregnancy is essential for supporting fetal growth and newborn health.^1^ Gestational weight gain (GWG) is an important measure of maternal nutritional status during pregnancy^2^ ^3^ and has been associated with a wide range of adverse perinatal outcomes.^4^ In 2009, the Institute of Medicine (IOM; now the National Academy of Medicine) provided guidelines on the recommended ranges of GWG based on maternal body mass index (BMI) before pregnancy.^4^ Women who are underweight (BMI<18.5), normal weight (18.5-24.9), overweight (25-29.9), and obese (≥30) at conception are recommended to gain 12.5-18 kg, 11.5-16 kg, 7-11.5 kg, and 5-9 kg, respectively, during pregnancy.^4^ However, the IOM recommendations for GWG are based on data from high income countries only (primarily North America). More recently, the INTERGROWTH-21st Gestational Weight Gain (IG-GWG) international standards were released, which provide a normative tool to evaluate GWG among well nourished, educated, normal weight women from geographically diverse settings with satisfactory maternal, perinatal, and infant outcomes.^5^ Inadequate GWG has previously been associated with an increased risk of low birthweight, small for gestational age, and preterm birth. In contrast, excessive GWG has been associated with an increased risk of large for gestational age and macrosomia.^4^ ^6^ ^7^ ^8^ Adverse birth outcomes are associated with a higher risk of mortality, suboptimal infant growth, neurodevelopmental delay, and suboptimal cardiometabolic outcomes later in life.^9^ ^10^ ^11^ ^12^ Therefore, supporting optimal weight gain during pregnancy could be an important pathway to reduce the risk of adverse birth outcomes and long term health consequences.^13^

Most of the evidence on the associations between GWG and adverse perinatal and neonatal outcomes to date has been primarily based on data from high income countries^7^ ^14^ ^15^ ^16^ ^17^ ^18^ ^19^ ^20^ ^21^ where only about 5-9% of the global burden of adverse birth outcomes is estimated to occur.^22^ ^23^ ^24^ In contrast, a disproportionate number of the estimated 14.6 million preterm births, 20 million low birthweight births, and 23 million small for gestational age births occur in countries in sub-Saharan Africa and South Asia.^22^ ^23^ ^24^ Although the epidemiology of maternal nutritional status at conception, GWG, and birth outcomes differs substantially for high income countries compared with low and middle income countries, only a limited number of studies have examined the association between GWG and neonatal outcomes in low and middle income countries.^8^ ^25^ ^26^ ^27^ For example, in one of the largest systematic reviews and meta-analyses examining the role of GWG in perinatal outcomes, which included individual level data from 1.3 million pregnancies in 18 studies, almost all studies were from high income settings in North America, Europe, and Asia (with the exception of four studies from mainland China); most of the participants (76%) were from North America and Europe.^6^ ^7^ Approximately 7% of women in that study were underweight, 18% were overweight, and 20% had obesity based on their BMI before pregnancy, with 23% and 47% of women overall gaining weight below and above the IOM recommendations, respectively.^4^ ^6^

While overweight and obesity at the start of pregnancy and excessive GWG during pregnancy are major concerns in high income settings,^28^ evidence from population representative surveys in 67 low and middle income countries suggests that inadequate GWG remains a substantial public health issue. On average, women in sub-Saharan Africa and South Asia are estimated to gain less than 60% of the minimum recommended GWG for women of normal weight based on the IOM guidelines.^29^ Recent evidence from systematic reviews and meta-analyses of relatively few studies conducted in sub-Saharan Africa,^8^ Brazil,^25^ and Asia^27^ suggests that the associations between inadequate and excessive GWG and newborn outcomes might be consistent with observations from high income settings. However, most of the studies included in these reviews had cross sectional, case-control, or retrospective cohort study designs, used metrics of GWG that are likely to be confounded by gestational duration, were noted to have poor control of confounding variables, and examined associations primarily with newborn weight.

To understand the contribution of GWG to adverse birth outcomes in the context of low and middle income countries, where the burden of adverse birth outcomes is greatest, we aimed to quantify the associations between GWG and a wide range of neonatal outcomes using individual participant data from prospective pregnancy studies conducted in these countries. We further aimed to determine whether the association between GWG adequacy and neonatal outcomes varied among subgroups of women based on their age and BMI before pregnancy. We also assessed the adequacy of maternal GWG using the IOM guidelines and the IG-GWG standards. The findings of this study will provide robust evidence on the associations between GWG adequacy and the risk of adverse neonatal outcomes, identifying potential subgroups of women at higher risk of adverse outcomes who might benefit from public health interventions.

## Methods

### Study and participant inclusion

In February and March 2019, we conducted a systematic search in PubMed, Embase, and Web of Science to identify prospective longitudinal studies from low and middle income countries that had weighed women during pregnancy. Search terms included MeSH headings and keywords related to pregnancy, weight gain, randomised trials or prospective cohort studies, and names of individual low and middle income countries. There were no language restrictions; however, we only examined titles and abstracts published after the year 2000 to capture relatively recent studies for generalisability of results. Titles and abstracts were screened in duplicate and full text reviews were performed on all selected abstracts independently by two team members to ensure that repeated measures of weight were available. We selected randomised controlled trials and prospective cohort studies for inclusion if they had prospectively measured weight, gestational age, and maternal height during pregnancy, and were not conducted exclusively among women with HIV or women with other health conditions that could limit the generalisability of the results. Studies that included self-reported maternal weight during pregnancy were not included. Because our primary aim was to identify eligible studies for inclusion in the individual participant data meta-analysis, we did not prospectively register the systematic search as a standalone review in a PROSPERO registration.

Individual study investigators (first, last, and corresponding authors) were then invited to participate in a survey designed to assess study eligibility and to indicate their willingness to participate in the data contribution effort for the GWG Pooling Project Consortium (supplementary fig 1). Among the 337 investigators contacted, 50% responded to the survey and identified 145 studies that were eligible for inclusion. Non-response or delay in executing the data contributor agreement, withdrawal, missingness of gestational age data or retrospective data collection led to the exclusion of several studies (supplementary fig 1). Data from 53 studies were included in this pooled analysis examining the associations between GWG adequacy and neonatal outcomes (supplementary tables 1-3).^30^ ^31^ ^32^ ^33^ ^34^ ^35^ ^36^ ^37^ ^38^ ^39^ ^40^ ^41^ ^42^ ^43^ ^44^ ^45^ ^46^ ^47^ ^48^ ^49^ ^50^ ^51^ ^52^ ^53^ ^54^ ^55^ ^56^ ^57^ ^58^ ^59^ ^60^ ^61^ ^62^ ^63^ ^64^ ^65^ ^66^ ^67^ ^68^ ^69^ ^70^ ^71^ ^72^ ^73^ ^74^ ^75^ ^76^ ^77^ ^78^ We systematically assessed that the variables included in the analysis were defined consistently across all studies and evaluated the potential for selection, attrition, and measurement biases for each study using the Quality in Prognosis Studies tool (supplementary table 4).^79^ Participants were excluded from these analyses if data on gestational age, date of delivery, or maternal height (an essential measure for the assessment of maternal BMI before pregnancy) were missing. Women who were living with HIV and those who had multiple fetuses were also excluded (3173 of 121 380; 2.6%).

### GWG assessment

Maternal GWG was defined as the difference between the final weight measure before delivery and the first trimester weight. Gestational age was assessed using ultrasound based measures or date of last menstrual period. For participants for whom first trimester weight was unavailable (33%), we imputed values by deriving subject specific slopes and intercepts from a mixed effects restricted cubic spline model regressing weight on gestational age with 3 knots based on the pooled database, stratified by geographical region. The methods and validation procedures for the imputation have been described in detail elsewhere.^80^ Briefly, compared with other imputation strategies, including nearest measure, simple arithmetic imputation based on the nearest two measures, and marginal models with generalised estimating equations, we observed that mixed effects imputation models that allow for appropriate degree of flexibility to accommodate nonlinear patterns showed the highest accuracy between observed and predicted early pregnancy weights (mean absolute error 1.99 kg and 1.60 kg) in two pregnancy cohorts in Tanzania with repeated pregnancy weights. We imputed first trimester weight at nine weeks of gestation, as opposed to the estimated date of conception, to balance the degree of extrapolation (imputing values farther away from the centre of the available data for studies with no first trimester weight). To assess maternal BMI before pregnancy, we used the imputed or observed first trimester weight, when available, as a proxy measure. Women were classified as being underweight (BMI<18.5), normal weight (18.5-24.9), overweight (25-29.9), or obese (≥30) based on the World Health Organization definitions. For adolescent mothers (<20 years at enrolment), we used the WHO BMI for age growth references^81^ to classify underweight (less than −2 standard deviations), normal weight (−2 to <1 standard deviation), overweight (1 to <2 standard deviations), or obese (≥2 standard deviations).

We used the GWG adequacy ratio based on the IOM guidelines as a primary exposure of interest because it is independent of gestational duration^82^; this ratio has also been previously used in the context of low and middle income countries.^83^ The GWG adequacy ratio was derived as a ratio of the observed weight gain between the first and last weight measures over the recommended weight gain in the same gestational duration and expressed as a percentage (equation 1): 

% Adequacy GWG = (Observed GWG ÷ Recommended GWG) × 100

The recommended weight gain according to IOM guidelines was estimated as follows (equation 2):

Recommended GWG at the last observed weight measure =

((BMI specific expected first trimester weight ÷ 13.86 weeks) × (13.86 weeks − GA at first observed or imputed weight measure))

+ ((GA at last weight measure − 13.86 weeks) × BMI specific recommended mean rate of GWG in the second and third trimesters)

where GA represents the gestational age, and the recommended weight gain for each participant was the sum of the following: the BMI specific expected first trimester weight gain (2 kg for underweight or normal weight, 1 kg for overweight, 0.5 kg for women classified as obese) for the number of weeks since the observed or imputed first trimester weight until the end of the first trimester (13^6/7^ weeks=13.86 weeks); and the weight gain up to the last weight measure based on the BMI specific recommended mean rate of weight gain in the second and third trimesters (0.51 kg/week for women who are underweight, 0.42 kg/week for women of normal weight, 0.28 kg/week for women who are overweight, and 0.22 kg/week for women with obesity).^4^

In sensitivity analyses, we estimated the GWG adequacy ratio using the lower limit of the recommended range of weight gain in the first trimester (0.5 kg) and the lower bound of the mean rate of weight gain in the second and third trimester: 0.44 kg/week for women who were underweight, 0.35 kg/week for women of normal weight, 0.23 kg/week for women who were overweight, and 0.17 kg/week for women with obesity. The GWG adequacy ratio was derived as a continuous measure and categorised as follows for analyses: severely inadequate (<70%), moderately inadequate (70% to <90%), adequate (90% to <125%), and excessive (≥125%). The cutoffs of <90% and >125% were chosen because they correspond to the upper and lower limits of the IOM recommended weekly GWG range. For example, as illustrated by Adu-Afarwuah and colleagues,^83^ the expected GWG by 40 weeks of gestation for women of normal weight is 2.0 kg+((40 weeks−13 weeks)×0.4 kg/week)=12.8 kg. The IOM’s recommended total weight gain range for women of normal weight is 11.5-16.0 kg. These values correspond to 90% (11.5 kg/12.8 kg×100) and 125% (16 kg/12.8 kg×100) of the 12.8 kg expected weight gain. However, IOM based categorisations are based on data from high income countries only and therefore might not capture the wide range of weight gain possible in other populations. As a result, to capture the severity of inadequate GWG in these data from low and middle income countries, we created an additional category (<70%) to reflect this. We also used IG-GWG standards to derive standardised indices (z scores) of GWG among women of normal weight.^5^ Outlying values, defined as observations with GWG z score greater than or less than 6 standard deviations from the median, were excluded from analysis. GWG z scores were categorised into four categories for analyses: very low (less than −2 standard deviations), low (−2 to less than −1 standard deviations), adequate (−1 to less than 1 standard deviation), or high (at least 1 standard deviation).

### Outcome definitions

We examined the associations between GWG adequacy and nine neonatal outcomes related to timing of birth, mortality, and size at birth that are common indicators of neonatal health (table 1). Newborn anthropometry was conducted within 48 hours of birth for 96.7% of the observations. Anthropometric outcomes at birth, such as small for gestational age, large for gestational age, short for gestational age, and microcephaly, were defined using the INTERGROWTH-21st international newborn size standards for gestational age and sex.^84^

**Table 1:** Neonatal outcomes examined and their operational definitions

Neonatal outcome	Definition
Stillbirth	Fetal death between 28 weeks gestational age and delivery
Neonatal death	Newborn death <28 days of life
Preterm birth	Birth at <37 weeks gestational age
Low birthweight	Birthweight <2500 g
Small for gestational age	Birthweight <10th centile of IG-NS
Large for gestational age	Birthweight >90th centile of IG-NS
Macrosomia	Birthweight >4000 g
Short for gestational age	Length for gestational age z score less than −2 SD at birth based on IG-NS
Microcephaly	Head circumference for gestational age z score less than −2 SD at birth based on IG-NS

IG-NS=INTERGROWTH-21st newborn size standards; SD=standard deviation.

### Confounders

**Fig 1:**
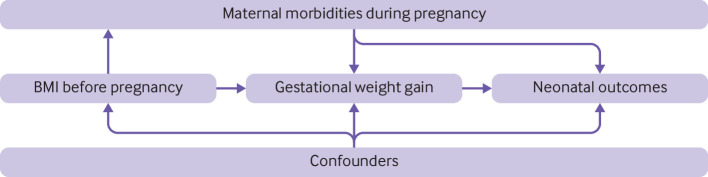
Directed acyclic graph of association between gestational weight gain and neonatal outcomes. Confounders included maternal age, parity, gravidity, maternal education, marital status, wealth index, smoking status, malaria infection, and intervention group assignment (if applicable). Maternal morbidities included any hypertension and gestational diabetes. BMI=body mass index

Guided by the causal structure (fig 1), we identified confounders of the association between GWG adequacy and neonatal outcomes a priori based on previous literature^4^ ^8^ ^17^ ^18^ ^85^ ^86^ ^87^ ^88^ ^89^ ^90^ and expert knowledge. Confounders included maternal age at the time of the study (<20, 20-24, 25-29, or ≥30 years), parity (0, 1, 2, or ≥3), gravidity (1, 2, 3, or ≥4), maternal education (0-4, 5-7, 8-11, or ≥12 years), marital status (yes or no), wealth index, smoking status (yes or no), malaria (yes or no), any hypertension (yes or no), gestational diabetes (yes or no), and intervention group (if any). Additionally, we considered other potential confounders of interest, including family violence, food security, dietary diversity, physical activity during pregnancy, maternal depression, stress, and social support; however, data on these factors were largely unavailable across studies. We did not adjust for maternal reproductive history because adjusting for such factors can introduce bias and underestimate the association for factors in the current pregnancy.^91^ We adjusted for hypertension (chronic or gestational) and gestational diabetes, when data permitted, because in the context of studying the association with total GWG adequacy, as a composite variable of cumulative weight gain, the time varying nature of GWG is reduced to a single time point and associations with newborn outcomes would be confounded by gestational hypertension and diabetes.^92^ The availability of key confounders of the association between GWG adequacy and neonatal outcomes was highly variable across studies (supplementary table 1). Variables consistently available across all studies included maternal age, maternal BMI before pregnancy, and study intervention arm (if any). Therefore, we adjusted for confounders in a two stage meta-analysis. This approach has the advantage of adjusting for all available confounders within each study. In sensitivity analyses, we investigated the potential influence of including studies with fewer confounders available sequentially on the pooled effect size when using a two stage meta-analytic approach.

### Statistical analyses

We used a two stage meta-analytic approach to estimate the associations between GWG adequacy ratio and neonatal outcomes. In the first stage, we used modified Poisson regression with robust standard error to estimate the relative risks (with 95% confidence intervals) of neonatal outcomes as a function of GWG adequacy for each study separately. Because of convergence issues with modified Poisson regression, we used Firth’s logistic regression to estimate the odds ratios (with 95% confidence intervals) for measures of association with GWG for neonatal outcomes with very low prevalence at the study level (primarily stillbirth, neonatal death, and macrosomia) to account for very low or zero cell counts. Odds ratios approximate the relative risk in the context of rare outcomes. We then combined individual study estimates using a DerSimonian-Laird random effects meta-analytic approach to estimate the pooled association between GWG adequacy and neonatal outcomes. We excluded study specific estimates from the second stage of the meta-analysis if the cross tabulation cell count for a given GWG adequacy stratum (severely inadequate, moderately inadequate, or excessive) and a given neonatal outcome in a study was less than three or if the reference category (adequate GWG) had a cell count of zero as this resulted in implausible or extremely unstable point estimates. However, the study overall was not excluded and estimates for associations between other GWG adequacy stratums and neonatal outcomes with a minimum cell count of three were included in the meta-analysis. In sensitivity analyses we included all studies in the second stage of the meta-analysis, irrespective of instability or extreme point estimates due to zero cell counts in the reference category, to check for robustness of primary inferences. We used the I^2^ statistic to report the proportion of total variability caused by between study variability. However, because I^2^ tends to increase with accumulating evidence (closer to 100% as the number of participants increases), τ^2^ was used to assess the degree of underlying between study variance.^93^ To ensure robustness of our inferences, we conducted sensitivity analyses using a missing indicator approach to account for confounder missingness and examined associations between GWG adequacy and neonatal outcomes among participants with their last weight measure in the third trimester.

We used one stage meta-analysis to assess whether associations between GWG adequacy and neonatal outcomes were modified by maternal age and BMI before pregnancy. This approach allowed us to minimise further issues of low cell counts by pooling data across studies. We then used modified Poisson or logistic regression models with study as a fixed effect rather than a random effect to be more conservative. The minimum set of confounders available across all studies included maternal age, BMI before pregnancy, and study intervention arm, if applicable. To separate the within and between study heterogeneity, we applied a one stage model with centred covariates and used the variable means as additional covariates in the models^94^ ^95^ (supplementary online text, p. 16). Because of the small proportion of women classified as having obesity based on their BMI before pregnancy across all studies, we combined women with overweight and obese BMI classifications before pregnancy for interaction analyses. We used a significance level of 0.05 for interaction terms when assessing effect modification of associations between GWG adequacy and neonatal outcomes by maternal age and BMI before pregnancy. We did not adjust for multiple hypothesis testing because this might be unnecessary and constrain the ability to detect heterogeneity in effects.^96^ We further explored the pattern in the associations between GWG adequacy ratio, as a continuous measure centred on study specific means, and neonatal outcomes using one stage mixed effects robust Poisson or logistic regression models with restricted cubic splines, where study was included as a random intercept and slope to obtain study specific nonlinear associations. All analyses were conducted in Stata version 16 (College Station, Texas, USA) and were guided by a prospectively developed statistical analysis plan that was reviewed by technical advisory group members.

### Patient and public involvement

This study was a secondary data analysis of deidentified existing datasets, which did not involve new direct contact with participants. For all parts of these secondary data analyses, participants, care givers, and lay people were not involved in the development of the research question, study design, or outcome measures, nor the interpretation or writing up of the results. Some of the original studies contributing data to this analysis included recruitment of participants by lay community health workers.

## Results

This meta-analysis included 53 studies with 118 207 participants. Most studies were from sub-Saharan Africa (34%), Latin America and the Caribbean (28%), and South Asia (25%; table 2). Most women (75%) were <30 years of age, with 18% being <20 years of age. More than half of the women had up to seven years of education (57%). The proportion of women who were underweight before pregnancy was 15.6%, 63.9% of women were of normal weight, and 20.5% were overweight or had obesity (table 2). Overall, severely inadequate or moderately inadequate GWG was observed among 36.5% and 18.9% of women, respectively, and excessive GWG was observed among 22.6% of women (table 2). The prevalence of small for gestational age, low birthweight, and short for gestational age was 28.8%, 16.9%, and 13.5% of newborns, respectively. The prevalence of preterm birth and microcephaly was 12.1% and 10.7%, respectively. The least prevalent neonatal outcomes included stillbirth, neonatal death, and macrosomia (all <3%). Supplementary tables 2, 3, and 5 summarise the distribution of maternal BMI before pregnancy, GWG adequacy, and neonatal outcomes for each study.

**Table 2:** Summary of maternal characteristics and neonatal outcomes

Maternal characteristics and infant outcomes	No of studies or participants (%)
**World region**	
Latin America and Caribbean	15 (28.3)
North Africa and the Middle East	1 (1.89)
South Asia	13 (24.5)
Sub-Saharan Africa	18 (34.0)
South-east Asia, East Asia, and Oceania	6 (11.3)
**Study type**	
Intervention	27 (50.9)
Cohort	26 (49.1)
**Maternal age (years)**	
<20	20 692 (17.6)
20-24	35 158 (29.9)
25-29	32 563 (27.7)
≥30	29 195 (20.5)
**Maternal education (years)**	
0-4	28 477 (32.9)
5-7	21 136 (24.4)
8-11	21 938 (25.3)
≥12	15 039 (17.4)
Maternal BMI before pregnancy*	
Underweight (<18.5)	18 895 (15.6)
Normal weight (18.5-24.9)	76 988 (63.9)
Overweight or obese (≥25)	24 646 (20.5)
**Gestational weight gain adequacy (%)**	
Severely inadequate (<70)	43 186 (36.5)
Moderately inadequate (70 to <90)	22 319 (18.9)
Adequate (90 to <125)	25 956 (22.0)
Excessive (≥125)	26 746 (22.6)
**Neonatal outcomes**	
Preterm birth	14 094 (12.1)
Low birthweight	17 548 (16.9)
Small for gestational age	29 691 (28.8)
Large for gestational age	8705 (8.43)
Stillbirth	1409 (1.31)
Neonatal death	1598 (2.26)
Short for gestational age	12 607 (13.5)
Microcephaly	9272 (10.7)
Macrosomia	2151 (2.07)

* For adolescent women <20 years we used World Health Organization body mass index (BMI) for age standards to define underweight (less than −2 standard deviations), normal weight (−2 to less than 1 standard deviation), overweight (1 to less than 2 standard deviations), or obese (at least 2 standard deviations).

### Associations between GWG adequacy and neonatal outcomes

Table 3 summarises the associations between GWG adequacy and neonatal outcomes (supplementary figs 2-10). Compared with women with adequate GWG, women with severely inadequate GWG had a higher risk of having newborns with low birthweight (relative risk 1.62, 95% confidence interval 1.51 to 1.72; n=48; τ^2^=0.006), small for gestational age (1.44, 1.36 to 1.54; n=51; τ** ^2^=0.016), short for gestational age (1.47, 1.29 to 1.69; n=40; τ^2^=0.074), and microcephaly (1.57, 1.31 to 1.88; n=31; τ^2^=0.145). Women with moderately inadequate GWG similarly had a higher risk of having newborns with low birthweight, small for gestational age, and microcephaly compared with those with adequate GWG; however, the magnitudes of the associations were attenuated. Women with excessive GWG had a higher risk of preterm birth (1.22, 1.13 to 1.31; n=48; τ^2^=0.008) and having newborns large for gestational age (1.44, 1.33 to 1.57; n=47; τ^2^=0.009) and with macrosomia (1.52, 1.33 to 1.73; n=29; τ^2^=0) compared with those with adequate GWG. Inferences remained unchanged when analyses were restricted to participants with their last weight measure in the third trimester (supplementary table 6) and when using a missing indicator approach (data not shown). Inferences were also similar when using the lower rate of mean weight gain (supplementary table 7) and when using a one stage meta-analysis approach to examine the association between GWG adequacy and neonatal outcomes (supplementary table 8). We observed nonlinear associations between GWG adequacy ratio and several neonatal outcomes using pooled data (data not shown), but were unable to estimate the study specific nonlinear associations for some neonatal outcomes owing to rare events.

**Table 3:** Adjusted associations between gestational weight gain (GWG) adequacy categories at last weight measure and neonatal outcomes in two stage meta-analysis

Neonatal outcomes	No of studies	No of participants	Severely inadequate GWG (<70%)				Moderately inadequate GWG (70% to <90%)				Excessive GWG (≥125%)		
			Relative risk (95% CI)	I^2^ (%)	τ^2^		Relative risk (95% CI)	I^2^ (%)	τ^2^		Relative risk (95% CI)	I^2^ (%)	τ^2^
Preterm birth	48	103 762	1.06 (0.95 to 1.18)	67	0.059		1.02 (0.94 to 1.11)	35	0.017		1.22 (1.13 to 1.31)	16	0.008
Low birthweight	48	93 337	1.62 (1.51 to 1.72)	15	0.006		1.26 (1.20 to 1.32)	0	0		0.92 (0.83 to 1.02)	16	0.014
Small for gestational age	51	93 191	1.44 (1.36 to 1.54)	55	0.016		1.22 (1.18 to 1.26)	0	0		0.79 (0.75 to 0.83)	0	0
Large for gestational age	47	90 044	0.65 (0.57 to 0.74)	55	0.064		0.82 (0.72 to 0.88)	0	0		1.44 (1.33 to 1.57)	20	0.009
Stillbirth	20	60 470	1.11 (0.88 to 1.41)	28	0.064		1.02 (0.85 to 1.24)	0	0		1.11 (0.89 to 1.38)	0	0.000
Neonatal death	18	56 654	0.90 (0.71 to 1.14)	37	0.073		0.86 (0.72 to 1.02)	0	0		1.12 (0.88 to 1.42)	0	0.000
Short for gestational age	40	83 827	1.47 (1.29 to 1.69)	68	0.074		1.21 (1.11 to 1.31)	16	0.007		0.88 (0.81 to 0.97)	4.30	0.003
Microcephaly	31	80 046	1.57 (1.31 to 1.88)	78	0.145		1.26 (1.12 to 1.41)	38	0.026		0.88 (0.79 to 0.99)	4.30	0.003
Macrosomia	29	68 138	0.59 (0.49 to 0.72)	0	0		0.62 (0.51 to 0.76)	0	0		1.52 (1.33 to 1.73)	0	0

Multivariable adjusted relative risk based on two stage meta-analysis of study specific models for each neonatal outcome adjusted for maternal age, maternal body mass index before pregnancy, study intervention arm (if applicable), and other important confounders available in each study. Reference category: adequate GWG (90-125%). For stillbirth, neonatal death, and macrosomia, measures of association with GWG adequacy were estimated using odds ratio given very low prevalence of these outcomes and convergence issues with modified Poisson regression. Odds ratios approximate relative risk in context of rare outcomes.

### Effect modification by maternal BMI before pregnancy

**Fig 2:**
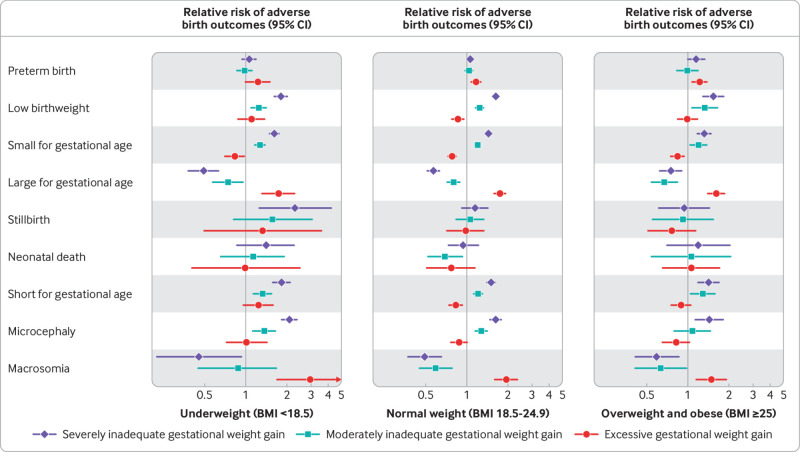
Effect modification of associations between gestational weight gain (GWG) adequacy and neonatal outcomes by maternal body mass index (BMI) before pregnancy: underweight (BMI<18.5), normal weight (18.5-24.9), and overweight or obese (≥25). Results are shown for associations between GWG adequacy (severely inadequate <70%, moderately inadequate 70% to <90%, excessive ≥125% compared with reference category of adequate GWG, 90% to <125%) and neonatal outcomes using multivariable modified Poisson regression models with robust variance adjusted for maternal age, study intervention arm (if applicable), and study fixed effects using a one stage meta-analytic approach. For stillbirth, neonatal death, macrosomia, and large for gestational age, one stage logistic regression models were used. Relative risks on x axis are presented on log scale

There was notable heterogeneity in the magnitude of the associations between GWG adequacy and some neonatal outcomes by maternal BMI before pregnancy (fig 2; supplementary table 9). For example, associations between severely inadequate GWG and the risk of low birthweight (relative risk 1.81, 95% confidence interval 1.62 to 2.02; P for interaction=0.072), small for gestational age (1.62, 1.51 to 1.75; P for interaction=0.003), short for gestational age (1.83, 1.58 to 2.12; P for interaction=0.012), and microcephaly (2.09, 1.76 to 2.49; P for interaction=0.005) were greater in magnitude among women who were underweight compared with associations among women of normal weight (fig 2; supplementary table 9). Severely inadequate GWG was also associated with a higher risk of stillbirth among women who were underweight (2.30, 1.25 to 4.23; P for interaction=0.033), but not among women of normal weight. In contrast, among women who were overweight or with obesity, severely inadequate GWG was associated with a lower magnitude of risk of low birthweight, small for gestational age, short for gestational age, and microcephaly compared with associations observed among women of normal weight; however, the magnitudes of the associations were not statistically significantly different among these two subgroups of women. Excessive GWG was associated with a lower risk of low birthweight and short for gestational age among women of normal weight, but the risk of these outcomes was higher among women who were underweight, though the confidence intervals crossed the null (fig 2, left and middle panel). The magnitudes of the associations between excessive GWG and large for gestational age and macrosomia were larger among women of normal weight and underweight compared with associations observed among women with overweight or obesity. Patterns of association were similar, though some inferences changed, when analyses were restricted to participants with weight measured in the third trimester (supplementary table 10).

### Effect modification by maternal age

**Fig 3:**
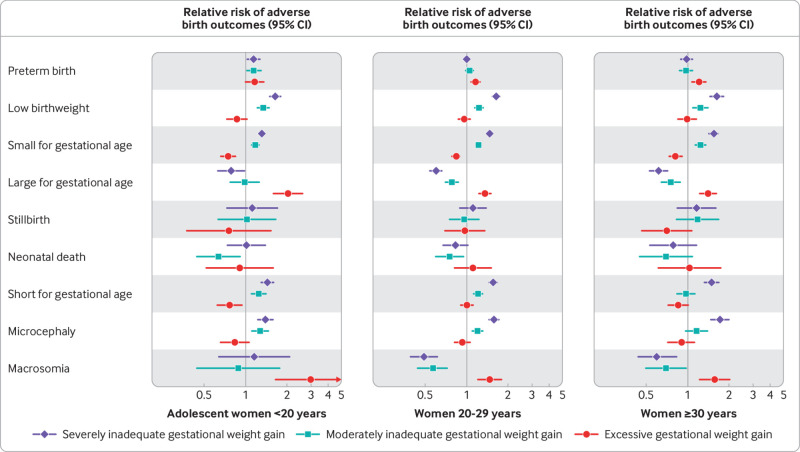
Effect modification of associations between gestational weight gain (GWG) adequacy and neonatal outcomes by maternal age. Results are shown for association between GWG adequacy (severely inadequate <70%, moderately inadequate 70% to <90%, and excessive ≥125% compared with reference category of adequate GWG, 90% to <125%) and neonatal outcomes using multivariable modified Poisson regression models with robust variance adjusted for maternal body mass index before pregnancy, study intervention arm (if applicable), and study fixed effects using one stage meta-analytic approach. For stillbirth, neonatal death, macrosomia, and large for gestational age, one stage logistic regression models were used. Relative risks on x axis are presented on log scale

The associations between GWG adequacy and neonatal outcomes were attenuated or strengthened in women aged <20 years and ≥30 years compared with those aged 20-29 years (fig 3; supplementary table 11). For example, severely inadequate GWG was associated with a higher risk of preterm birth (relative risk 1.14, 95% confidence interval 1.03 to 1.27; P for interaction=0.032) among women <20 years compared with associations among women aged 20-29 years; a similar pattern was observed for moderately inadequate GWG (fig 3, supplementary table 11). In contrast, excessive GWG was associated with a higher risk of preterm birth among women aged 20-29 years (1.16, 1.07 to 1.25) and ≥30 years (1.21, 1.07 to 1.36), but not among women <20 years (fig 3, middle and right panels; supplementary table 11). Moderately inadequate GWG, but not severely inadequate GWG, was associated with a lower risk of neonatal death among women <20 years (0.63, 0.43 to 0.91; P for interaction=0.41) and 20-29 years (0.75, 0.59 to 0.95), but not among older women (0.69, 0.44 to 1.08; P for interaction=0.76). The associations between excessive GWG and large for gestational age and macrosomia were strengthened among adolescent women <20 years compared with those aged 20-29 years; and excessive GWG was associated with a lower risk of short for gestational age (0.76, 0.61 to 0.93; P for interaction=0.02) among women <20 years compared with those aged 20-29 years (fig 3; supplementary table 11). Maternal age did not meaningfully modify the association between GWG adequacy and other neonatal outcomes (fig 3; supplementary table 11). Inferences were largely consistent when analyses were restricted to participants with the last weight measure in the third trimester (supplementary fig 12).

### Using GWG international standards

Associations between GWG z scores among women of normal weight and neonatal outcomes were similar to associations observed with GWG adequacy ratio based on the IOM guidelines, with one notable exception (table 4; supplementary figs 11-19). Compared with women with GWG z scores between −1 and less than 1 standard deviation, women with GWG z scores less than −2 standard deviations had a lower risk of preterm birth (relative risk 0.81, 95% confidence interval 0.71 to 0.93; n=44; τ^2^=0.070) and women with GWG z scores of at least 1 standard deviation had a higher risk of preterm birth (1.55, 1.35 to 1.79; n=44; τ^2^=0.036; table 4).

**Table 4:** Adjusted associations between gestational weight gain (GWG) z score categories derived using INTERGROWTH-21st GWG standards, and neonatal outcomes among women of normal weight

Neonatal outcomes	No of studies	No of participants	GWG z score less than −2 SD				GWG z score −2 to less than −1 SD				GWG z score ≥1 SD		
			Relative risk (95% CI)	I^2^ (%)	τ^2^		Relative risk (95% CI)	I^2^ (%)	τ^2^		Relative risk (95% CI)	I^2^ (%)	τ^2^
Preterm birth	44	72 107	0.81 (0.71 to 0.93)	67	0.070		0.86 (0.79 to 0.93)	40	0.016		1.55 (1.35 to 1.79)	31	0.036
Low birthweight	43	63 523	1.54 (1.36 to 1.75)	70	0.067		1.27 (1.15 to 1.41)	59	0.038		0.98 (0.79 to 1.21)	29	0.062
Small for gestational age	48	63 733	1.65 (1.47 to 1.87)	85	0.085		1.36 (1.26 to 1.47)	68	0.026		0.79 (0.67 to 0.93)	33	0.048
Large for gestational age	36	60 171	0.50 (0.41 to 0.61)	59	0.098		0.64 (0.56 to 0.72)	47	0.037		1.96 (1.69 to 2.28)	36	0.030
Stillbirth	17	35 154	0.97 (0.72 to 1.31)	28	0.085		0.90 (0.74 to 1.08)	0	0		2.33 (0.94 to 5.75)	46	0.428
Neonatal death	14	44 299	0.95 (0.77 to1.18)	10	0.015		0.84 (0.68 to 1.03)	21	0.027		1.06 (0.52 to 2.18)	0	0.000
Short for gestational age	32	56 136	1.71 (1.43 to 2.05)	82	0.127		1.39 (1.24 to 1.56)	61	0.039		0.94 (0.78 to 1.14)	2.80	0.003
Microcephaly	29	55 015	1.77 (1.45 to 2.15)	80	0.149		1.36 (1.18 to 1.55)	63	0.054		0.77 (0.61 to 0.97)	0	0
Macrosomia	17	27 751	0.81 (0.41 to 1.61)	63	0.640		0.68 (0.54 to 0.86)	0	0		2.70 (1.79 to 4.07)	57	0.171

Multivariable adjusted relative risk based on two stage meta-analysis of study specific models for each neonatal outcome adjusted for maternal age, maternal body mass index before pregnancy, study intervention arm (if applicable), and other important confounders available in each study. Reference category: GWG z score: −1 to <1 standard deviation. For stillbirth, neonatal death, and macrosomia, measures of association with GWG adequacy were estimated using odds ratio given very low prevalence of these outcomes and convergence issues with modified Poisson regression. Odds ratios approximate relative risk in context of rare outcomes.

SD=standard deviation.

### Sensitivity analyses

In sensitivity analyses, we examined the robustness of primary inferences for the associations between GWG adequacy and neonatal outcomes when studies with fewer confounders available were sequentially included in the two stage meta-analysis (supplementary figs 20-28). Although we observed some variation in the point estimates, the confidence intervals overlapped when studies with a varying number of confounders were pooled. Additionally, including all studies in the second stage of the meta-analysis, irrespective of instability or extreme point estimates due to very low or zero cell counts in the reference category, did not change the primary inferences (data not shown).

## Discussion

### Principal findings

In this two stage meta-analysis of prospective longitudinal data from 53 studies conducted in low and middle income countries, the prevalence of suboptimal GWG was high, with 78% of women gaining inadequate or excessive weight during pregnancy. Compared with women with adequate GWG, those with severely or moderately inadequate GWG had a higher risk of having a newborn with low birthweight, small for gestational age, short for gestational age, and microcephaly, whereas women with excessive GWG were at a higher risk of having a newborn with large for gestational age and macrosomia. Excessive GWG was also associated with an increased risk of preterm birth. However, these associations were modified by maternal BMI before pregnancy. Associations between severely inadequate GWG and small size at birth were strengthened among women who were underweight but attenuated among women with overweight or obesity compared with associations among women of normal weight. Severely inadequate GWG was also associated with a higher risk of stillbirth among women who were underweight compared with those of normal weight, and a higher risk of preterm birth was found among women aged <20 years compared with those aged 20-29 years. In contrast, moderately inadequate GWG was associated with lower risk of neonatal death among women aged <20 years and 20-29 years.

### Comparison with other studies

Findings from this study suggest that the trend in the association between GWG and newborn weight related anthropometric indicators is similar across studies, and the patterns are in line with evidence from high income settings^6^ ^7^ ^15^ ^97^ ^98^ and previous studies from Latin America,^25^ ^26^ Asia,^27^ and sub-Saharan Africa.^8^ Weight gain during pregnancy is a cumulative measure of changing maternal physiology (fat free and fat mass accumulation, blood volume expansion), the placental weight, and the developing fetus (fat and fat free mass and amniotic fluid accretion).^4^ The availability and supply of nutrients to support fetal growth are dependent on maternal nutrient stores, dietary intake, placental function, and a complex array of hormonal and metabolic processes.^2^ ^3^ ^4^ Unlike birthweight, very few studies have previously examined the associations between GWG and birth length, a proxy measure of fetal skeletal growth, and head circumference at birth, which is a marker of fetal brain growth.^87^ ^99^ ^100^ Consistent with previous evidence, findings from this study suggest that women with severely inadequate GWG are also at higher risk of having newborns that are short for gestational age and with a smaller head at birth. However, findings from a study conducted in the Gambia found higher GWG to be positively associated with larger newborn head circumference in a linear fashion, whereas higher GWG z scores were associated with birth weight and length only after a threshold was reached. These findings suggest that brain growth might be prioritised over fetal weight and linear growth.^99^ Further research is needed to elucidate the mechanisms underlying the associations between GWG and birth length and head circumference, and to explore the prevalence and functional consequences of a small head size in relation to maternal weight gain during pregnancy.

We also observed a higher risk of preterm birth associated with excessive GWG and with severely and moderately inadequate GWG among adolescent mothers aged <20 years. One possible mechanism for this association might be that inadequate GWG is a marker for macronutrient and micronutrient deficiencies that result in preterm birth, particularly if nutritional insults occur early in pregnancy and could affect plasma volume expansion or lead to inadequate maternal tissue development to support the fetus until term.^101^ Conversely, excessive GWG could be indicative of metabolic imbalances and underlying disease processes (eg, hypertension and gestational diabetes) that increase the level of placental corticotrophin releasing hormone contributing to earlier parturition.^102^ However, evidence on the association between GWG and preterm birth has been mixed, with findings from the recent individual participant data meta-analyses from high income settings^6^ ^15^ showing a greater risk of preterm birth with suboptimal GWG, while another systematic review reported a lower risk of preterm birth associated with excessive GWG.^14^ We also found a lower risk of preterm birth among women of normal weight with GWG z score less than −1 standard deviation compared with women with GWG z scores between −1 and 1 standard deviation when using IG-GWG standards. While we are unsure of the mechanisms that might contribute to this finding, it should be noted that the classifications of inadequate GWG using IOM guidelines and IG-GWG are not directly comparable.

Systematic studies of the associations between GWG and mortality related outcomes, such as stillbirth and neonatal death, in low and middle income countries are lacking, probably because these outcomes would be rare in a single study. We did not observe an overall association between GWG adequacy and stillbirth and neonatal death; however, severely inadequate GWG was associated with a higher risk of stillbirth among women who were underweight, whereas moderately inadequate GWG was associated with a lower risk of neonatal death among women <30 years of age. These findings add to the sparse and conflicting evidence base examining the association between GWG and stillbirth and neonatal death. For example, a population based cohort study in Sweden found no association between GWG and stillbirth,^18^ whereas a recent multicentre case-control study from the United States found that low GWG measured by internally standardised z scores was associated with higher odds of stillbirth.^103^ Findings from a recent analysis of 85 822 pregnancies in the Danish Birth Cohort suggest that placental dysfunction and infections might partly explain the higher risk of stillbirth among women with low GWG,^104^ although the risk of unexplained intrauterine death in this Danish study was greater with low GWG compared with high GWG. Placental dysfunction, inflammation, metabolic abnormalities, and intrapartum events remain the most commonly cited mechanisms contributing to stillbirth.^105^ We are unsure why moderately inadequate GWG might be associated with a lower risk of neonatal death. Further research is needed to understand how GWG directly or indirectly (eg, through low birthweight or small for gestational age) influences mechanisms that contribute to perinatal death.

Maternal preconception nutritional status, as measured by maternal body size, is widely recognised as an important determinant of fetal growth.^2^ In line with previous evidence,^6^ ^7^ ^15^ ^106^ maternal BMI before pregnancy modified the associations between GWG and neonatal outcomes in this study. The magnitude of the associations between suboptimal GWG and newborn anthropometric outcomes was generally strengthened among women who were underweight and attenuated among women who were overweight or had obesity compared with associations among women of normal weight. This outcome might partly be due to differential underlying baseline risks for adverse neonatal outcomes among women who are underweight and overweight or have obesity.^106^ We observed a higher risk of low birthweight, small for gestational age, and short for gestational age associated with excessive GWG among women who were underweight before pregnancy, which could indicate extracellular fluid retention or weight accumulation caused by hormonal, metabolic, or inflammatory processes not directly related to fetal growth but disrupting feto-placental nutrient transfer.^4^

We also observed notable differences in the magnitude of the associations between GWG adequacy and neonatal outcomes based on maternal age, which has rarely been previously examined^87^ given the low prevalence of adolescent pregnancies in high income settings or in a single study. We observed a higher risk of low birthweight, small for gestational age, short for gestational age, and microcephaly associated with severely inadequate GWG, relative to associations with moderate or excessive GWG, among women <20 years of age. These findings are in line with the well recognised higher risk of adverse neonatal outcomes associated with adolescent pregnancies.^107^ Maternal-fetal competition for nutrients and the lack of adequate intake of macronutrients and micronutrients required to support growth of the young mother and the fetus are largely thought to contribute to the increased risk of small newborn size among adolescent mothers.^2^ Given the high prevalence of adolescent pregnancies in countries in sub-Saharan Africa^108^ and South Asia, GWG might be a particularly important indicator of maternal nutritional needs and fetal growth. However, the lower magnitude of risks of small size at birth associated with severely inadequate GWG among adolescent mothers compared with older women suggests that other pathways probably play an important role in birth size among young mothers. Strategies to support sexual and reproductive health rights for adolescent girls to delay pregnancy^109^ are likely to contribute to lowering the risk of adverse neonatal outcomes associated with adolescent pregnancies.

### Strengths and limitations of this study

There are several strengths of this study. We pooled data from 53 studies representing 24 countries from different regions of the world and used individual level participant data analysis to ensure consistency of the exposure, outcomes, and confounders across studies. We used GWG adequacy ratio as the primary metric in our analyses because it has the advantage of being largely independent of gestation duration. Other measures of GWG, such as total absolute weight gain (in kg), are susceptible to confounding by gestational duration and were not used.^82^ ^110^ We also examined a wide range of outcomes to assess the implications of GWG adequacy comprehensively given that the direction of associations with inadequate and excessive GWG differs by the type of neonatal outcome.

However, the limitations of this study are important to acknowledge. We derived a metric of GWG adequacy that was based on IOM guidelines, which were developed using data from pregnant women in high income settings only. We used the international IG-GWG standards to assess adequacy of GWG among women of normal weight relative to a reference population of healthy pregnancies from geographically diverse populations^5^; however, these standards are based on a population of women of normal weight only and are therefore not directly applicable to women who are underweight, overweight, or have obesity. As a result, the thresholds used to assess GWG adequacy based on IOM recommendations might not accurately reflect GWG adequacy across diverse populations of pregnant women from different settings. Using the lower limit of the recommended rate of weight gain in the second and third trimesters in sensitivity analyses largely did not change the primary inferences. The consistency of our findings with those of previous studies also provides some evidence that risks associated with GWG outside the IOM based optimal ranges are relatively robust across populations. However, further work is needed to determine thresholds for adequate GWG among women in diverse populations in low and middle income countries. We chose to align the categorisation of GWG adequacy with the evidence based IOM recommendations based on their potential for clinical impact, but explored nonlinearity of the associations between GWG adequacy as a continuous measure and each neonatal outcome. Further research is needed to examine nonlinear trends in the association between GWG adequacy ratio, particularly for neonatal outcomes with low prevalence in this study.

Gestational age was assessed using ultrasound based measures or date of last menstrual period and therefore imprecision in the estimates might occur. Although we adjusted for confounders in each study in a two stage meta-analytic approach, data on confounders were not consistently available across studies. We also conducted sensitivity analyses to test the validity of our inferences with varying numbers of confounders in the two stage analysis, however we cannot rule out the possibility of unmeasured confounding. Additionally, because there were fewer women across studies who were overweight or had obesity based on their BMI before pregnancy or fewer women with excessive GWG, these findings are limited by small sample sizes for excessive GWG and neonatal outcomes, particularly in the analyses examining effect modification. Future research should also prioritise examining risks of inadequate or excessive GWG among women who are overweight or have obesity in low resource settings because many low or middle income countries are undergoing nutrition transition. We also used standardised definitions to evaluate exposure, outcomes, and covariates across studies and observed low risk of bias in participant inclusion and measurement overall; however, the potential for selection bias due to attrition in some studies cannot be eliminated.

Finally, while we used data from a large number of prospective cohorts and randomised controlled trials, with repeated weight measures during pregnancy, we did not have weight measures for approximately one third of women before pregnancy or in the first trimester, and therefore imputed these values based on predictions from a mixed effects model with restricted cubic splines. We did not draw imputed values from a predicted distribution using a multiple imputation approach, which might have affected the uncertainty in maternal BMI before pregnancy and GWG adequacy ratio. Heterogeneity in measures of association between GWG and neonatal outcomes between studies might in part be due to interventions in the trials. Further research is needed to investigate the effect of specific interventions that support optimal GWG and the mediating and modifying role of GWG in reducing the risk of adverse birth outcomes.

### Implications and conclusions

The WHO antenatal guidelines for positive pregnancy experience^13^ recommend that all pregnant women should be provided with counselling about nutrition, healthy diet, and physical activity to support optimal GWG. Therefore, weight monitoring at antenatal visits starting in the first trimester is a central component of care during pregnancy and will be important to track progress in improving maternal nutritional status and measuring the impact of public health interventions and policies. With over three quarters of the women included in this study gaining suboptimal weight (inadequate or excessive GWG), greater efforts are needed for weight monitoring and nutrition counselling during antenatal visits. This shortfall might partly be due to a lack of international consensus on what guidelines should say to support healthy maternal weight and which policies might be most effective.^111^ Most women in low resource settings do not have their weight measured despite recommendations and few receive nutritional counselling during antenatal care visits.^112^ Multisectoral approaches are needed because maternal weight monitoring alone without complementary interventions might not be an adequate strategy to support optimal GWG.^113^ Additionally, several cultural beliefs and practices, economic factors, and food preferences could hinder optimal GWG during pregnancy.^112^ Lifestyle interventions, particularly for reducing the risk of excessive GWG, have shown some benefit^114^; however, current data are primarily from high income countries. Interventions to support macronutrient and micronutrient requirements of pregnant women, including improving quality of the diet,^115^ multiple micronutrient supplements, small quantity lipid based nutrient supplements,^76^ ^116^ or balanced energy protein supplementation,^117^ might be useful strategies to reduce the prevalence of inadequate GWG. However, further evidence is needed to determine GWG adequacy thresholds among women in low and middle income countries and then to assess the efficacy, targeting, and subsequent benefits of these interventions to support optimal GWG in vulnerable women. Further research is also needed to understand the association of antimalarial and antiretroviral drugs, which are routinely provided in antenatal care settings to prevent or treat infectious diseases, with GWG adequacy.

We present findings from a large individual participant data meta-analysis of pregnancy studies conducted in low and middle income countries to assess the association of GWG with a wide range of neonatal outcomes. Our findings suggest that inadequate and excessive GWG are associated with an increased risk of suboptimal newborn anthropometric outcomes and with preterm birth, although the associations between suboptimal GWG and timing of birth are complex. It is important to note that we only considered associations between GWG adequacy and neonatal outcomes in this study; efforts to support optimal GWG should balance the benefits and risks for the mother and the infant. Further research is needed to elucidate the potential underlying mechanisms that explain the role of GWG adequacy in neonatal outcomes, particularly among subgroups of younger and older women, or those with low or high BMI before pregnancy. Holistic interventions that address the direct and indirect causes of suboptimal GWG among women in low and middle income countries are needed to support women to start pregnancies at an optimal age and nutritional status, and to maintain healthy pregnancies that minimise the risk of adverse neonatal outcomes.

**What is already known on this topic**

Gestational weight gain (GWG) during pregnancy is an important predictor of fetal and newborn health, however few studies have focused on the associations between GWG and neonatal outcomes in low and middle income countries
Few studies from Latin America, Asia, and sub-Saharan Africa have examined the association between GWG and birthweight, with limitations such as small sample sizes and poor control for confounding

**What this study adds**

Suboptimal weight gain (inadequate or excessive) was associated with an increased risk of low birthweight, small for gestational age, large for gestational age, macrosomia, low head circumference, and short for gestational age at birth
Women who were underweight, overweight or had obesity had a higher risk of adverse neonatal outcomes associated with suboptimal GWG compared with women of normal weight
Adolescent women younger than 20 years had a higher risk of some adverse neonatal outcomes associated with suboptimal GWG compared with women aged 20-29 years

## Data Availability

The original individual prospective study data used in this study are not available for data sharing. However, data for individual studies might be available from individual investigators upon reasonable request.

## Web extra

Extra material supplied by authors

Web appendix: Supplementary material

Web appendix: Members of consortium

## Notes

Contributors: NP, DQW, AMD, EL, MW, and WWF designed the study (project conception, development of overall research plan, and study oversight) with substantial input from technical advisory group members (PC, KGD, GK, SHK, and TA) and support from VS and BB for data curation. All authors of the GWG Pooling Project Consortium contributed data, provided feedback on the study methods and interpretation of the findings, and critically reviewed the manuscript for important intellectual content. NP, DQW, AMD, and EL had access to the pooled data. NP led the statistical analysis, drafted and iteratively revised the paper, has the primary responsibility for the final content, and is the guarantor. All GWG Pooling Project Consortium members reviewed and contributed to the final manuscript. KMH and BKN are deceased. The corresponding author attests that all listed authors meet authorship criteria and that no others meeting the criteria have been omitted. Please address correspondence to NP (nperumal@mailbox.sc.edu) and WWF (mina@hsph.harvard.edu).

GWG Pooling Project Consortium members: Ajibola Ibraheem Abioye, Manfred Accrombessi, Seth Adu-Afarwuah, Joao Guilherme Alves, Carla Adriane Leal de Araújo, Shams Arifeen, Rinaldo Artes, Per Ashorn, Ulla Ashorn, Omolola Olukemi Ayoola, Gabriela Chico-Barba, Robin Bernstein, Zulfiqar A Bhutta, Valérie Briand, Elvira Beatriz Calvo, Marly Augusto Cardoso, Yue Cheng, Peter Ellis Clayton, Shalean M Collins, John Kennedy Cruickshank, Delanjathan Devakumar, Christopher P Duggan, Pratibha Dwarkanath, Frankie J Fair, Henrik Friis, Alison D Gernand, Shibani Ghosh, Exnevia Gomo, Rebecca Grais, Ousmane Guindo, Guadalupe Estrada Gutierrez, K Michael Hambidge, Rezwanul Haque, Lieven Huybregts, Romaina Iqbal, Sheila Isanaka, Keith P West, Jr, José Roberto da Silva Junior, Maria Ome-Kaius, Joanne Katz, Subarna Khatry, Patrick Kolsteren, Nancy F Krebs, Teija Kulmala, Pratap Kumar, Anura V Kurpad, Carl Lachat, Anna Lartey, Qian Li, Jacqueline M Lauer, See Ling Loy, Nur Indrawaty Lipoeto, Laura Beatriz López, Abdullah Al Mahmud, G Arun Maiya, Kenneth Maleta, Maíra Barreto Malta, Dharma S Manandhar, Charles Mangani, Yves Martin-Prevel, Reynaldo Martorell, Susana L Matias, Elizabeth M McClure, Joshua D Miller, Marhazlina Mohamad, Hamid Jan Jan Mohamed, Sophie Moore, Paola S Mosquera, Malay Kanti Mridha, Ferdinand M Mugusi, Cinthya Muñoz-Manrique, Salifu Nanga, Barnabas K Natamba, Minyanga Nkhoma, David Osrin, Andrea B Pembe, Otilia Perichart-Perera, Ahmed Tijani Bawah, Eric Kwasi Ofori, Zul Premji, Andrew M Prentice, Juha Pyykkö, Preetha Ramachandra, Usha Ramakrishnan, Juan Rivera, Dominique Roberfroid, Ameyalli Rodríguez-Cano, Patricia Lima Rodrigues, Stephen J Rogerson, Hugo Martínez-Rojano, Patricia H C Rondó, Daniel E Roth, Reyna Sámano, Naomi M Saville, Saijuddin Shaikh, Bhim P Shrestha, Robin Shrestha, Hora Soltani, Sajid Soofi, Tinku Thomas, James Tielsch, Holger W Unger, Willy Urassa, Juliana dos Santos Vaz, Lee Wu, Nianhong Yang, Sera L Young, Lingxia Zeng, Chunrong Zhong, Zhonghai Zhu.

The lead author (NP) affirms that the manuscript is an honest, accurate, and transparent account of the study being reported; that no important aspects of the study have been omitted; and that any discrepancies from the study as planned (and, if relevant, registered) have been explained.

Dissemination to participants and related patient and public communities: Findings from this study will be published and made publicly available. Investigators in the GWG Pooling Consortium might share the results with local ministries of health, patients (including original study participants), and relevant medical organisations in the respective countries where the original studies were conducted.

Provenance and peer review: Not commissioned; externally peer reviewed.
