# Supplementary material for: Suboptimal gestational weight gain and neonatal outcomes in low and middle income countries: individual participant data meta-analysis
Source: BMJ. 2023 Sep 21;382:e072249. doi: 10.1136/bmj-2022-072249 (PMC10512803; doi:10.1136/bmj-2022-072249)
Supplement: Supplementary file 1 — Web appendix: Supplementary material [file pern072249.ww1.pdf]

Supplementary Material

Suboptimal gestational weight gain and neonatal outcomes in low- and middle-income countries: individual participant data meta-analyses

Perumal N, Wang D, Darling AM, Liu E, Wang M, Ahmed T, Christian P, Dewey KG, Kac G, Kennedy SH, Subramoney V, Briggs B, Fawzi WW
on behalf of the GWG Pooling Project Consortium

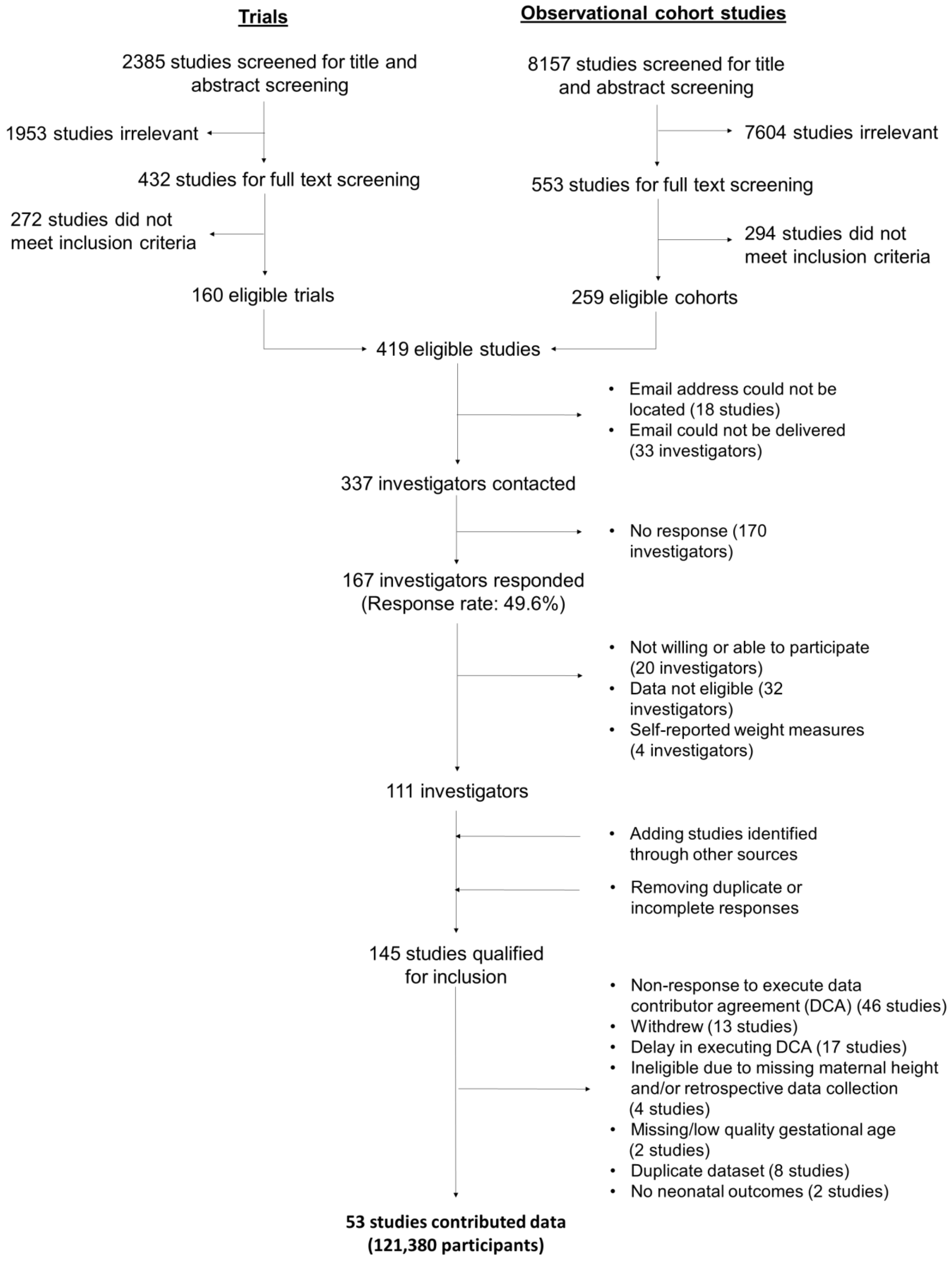

Supplementary Figure 1: Flowchart of study selection and data contribution.

Supplementary Table 1: Availability of data on *a priori* confounders of the association between GWG adequacy and neonatal outcomes.

Study acronym	Author, publication year	Confounders	No. of confounders
AKU-MMN	Bhutta 2009	Age, pre-pregnancy BMI, maternal education, parity, gravidity, hypertension	6
AKU-VITD	Khan 2016	Age, pre-pregnancy BMI, maternal education, parity, gravidity, hypertension, diabetes	7
Arg GWG Curves	Calvo 2009	Age, pre-pregnancy BMI, maternal education, parity, gravidity, hypertension	6
EU-MMN	Ramakrishnan 2003	Age, pre-pregnancy BMI, maternal education, parity, gravidity, hypertension, diabetes	7
FU-GWG	Rodrigues 2010	Age, pre-pregnancy BMI, maternal education, marital status, smoking, alcohol	6
FU-LEPTINGWG	Franco-Sena 2016	Age, pre-pregnancy BMI, maternal education, marital status, smoking, alcohol, gravidity, hypertension, diabetes	9
HERO-G	Moore 2020	Age, pre-pregnancy BMI, maternal education, parity, gravidity, hypertension, diabetes	7
HUST-TMCHC	Zhong 2019	Age, pre-pregnancy BMI, maternal education, parity, gravidity, hypertension, diabetes	7
ICDDR-MINIMat	Tofail 2008	Age, pre-pregnancy BMI, maternal education, marital status, wealth index, parity, hypertension	7
ILINS-DYAD-G	Adu-Afarwuah 2015	Age, pre-pregnancy BMI, maternal education, marital status, gravidity, malaria, hypertension	7
ILINS-DYAD-M	Ashorn 2015	Age, pre-pregnancy BMI, maternal education, marital status, smoking, alcohol, parity, gravidity, malaria, hypertension	10
IMIP-BRAMAG	de Araújo 2020	Age, pre-pregnancy BMI, maternal education, marital status, smoking, alcohol, gravidity, hypertension	8
IMIP-GestDM	do Nascimento 2019	Age, pre-pregnancy BMI, maternal education, marital status, smoking, alcohol, parity, gravidity, hypertension, diabetes	10
INPer-CAR	Sámano 2021	Age, pre-pregnancy BMI, maternal education, marital status, gravidity	5
INPer-FICA	Sámano 2017	Age, pre-pregnancy BMI, maternal education, marital status	4
INPer-GDM	Unpublished	Age, pre-pregnancy BMI, maternal education, marital status, parity, diabetes	6
INPR-NeuroObesity	Unpublished	Age, pre-pregnancy BMI, maternal education, marital status, smoking, alcohol, parity, gravidity, hypertension, diabetes	10
INPer-Poli	Unpublished	Age, pre-pregnancy BMI	2
INPer-REDES	Sámano 2018	Age, pre-pregnancy BMI, maternal education, gravidity, hypertension, diabetes	6
IRD-RECIPAL	Accrombessi 2018	Age, pre-pregnancy BMI, marital status, wealth index, malaria, hypertension	6
JHU-MothersGift	Tielsch 2015	Age, pre-pregnancy BMI, maternal education, marital status, smoking, alcohol, parity	7
JiVitA3	West 2014	Age, pre-pregnancy BMI, maternal education, marital status, wealth index, parity, gravidity	7
LAIS	Hallamaa 2019	Age, pre-pregnancy BMI, marital status, gravidity, malaria, diabetes	6
LCSS	Espo 2002	Age, pre-pregnancy BMI, maternal education, malaria	4
MRCG-ENID	Moore 2012	Age, pre-pregnancy BMI, maternal education, marital status, smoking, alcohol, parity gravidity, hypertension	9
MAHE-SCFPPP	Ramachandra 2016	Age, pre-pregnancy BMI	2
MAL1	Etheredge 2016	Age, pre-pregnancy BMI, maternal education, marital status, wealth index, parity, gravidity, hypertension	8
MAL2	Darling 2017	Age, pre-pregnancy BMI, maternal education, marital status, wealth index, parity, gravidity, hypertension	8
MDIG	Roth 2018	Age, pre-pregnancy BMI, maternal education, marital status, wealth index, parity, gravidity, hypertension	8
MINA-Brazil	Cardoso 2020	Age, pre-pregnancy BMI, maternal education, marital status, smoking, alcohol, parity, gravidity, malaria, hypertension, diabetes	11
MIRA-Janakpur	Osrin 2005	Age, pre-pregnancy BMI, maternal education, marital status, wealth index, parity, gravidity, hypertension	8
MISAME-1	Roberfroid 2008	Age, pre-pregnancy BMI, maternal education	3
MISAME-2	Huybregts 2009	Age, pre-pregnancy BMI, maternal education	3
NNIPS-3	Christian 2003	Age, pre-pregnancy BMI, maternal education, marital status, smoking, alcohol, wealth index, parity, gravidity	9
NWU-PMPEN	Widen 2019	Age, pre-pregnancy BMI, marital status, smoking, alcohol, parity, gravidity, malaria, hypertension, diabetes	10
NWU-PreNAPS	Widen 2017	Age, pre-pregnancy BMI, maternal education, marital status, parity, gravity, malaria	7
PNS	Fawzi 2007	Age, pre-pregnancy BMI, maternal education, marital status, smoking, alcohol, wealth index, parity, gravidity, malaria, hypertension	11
ROSE	Isanaka 2019	Age, pre-pregnancy BMI, maternal education, marital status, parity, gravidity, malaria, hypertension	8
SBUMS-GDM	Tehrani 2019	Age, pre-pregnancy BMI, marital status, smoking, gravidity, hypertension, diabetes	7

SHU-BMIGWG	Soltani 2017	Age, pre-pregnancy BMI, maternal education, gravidity, hypertension	5
SPAZ-IPTp	Unger 2015	Age, pre-pregnancy BMI, maternal education, smoking, alcohol, gravidity, malaria, hypertension	8
St-Johns	Unpublished	Age, pre-pregnancy BMI, maternal education, marital status, smoking, alcohol, gravidity, hypertension	8
TU-Aflatoxin	Lauer 2018	Age, pre-pregnancy BMI, maternal education, marital status, smoking, alcohol, parity, gravidity, hypertension	9
UC-RDNS	Matias 2016	Age, pre-pregnancy BMI, maternal education, marital status, gravidity, hypertension	6
UCL-LBWSAT	Saville 2018	Age, pre-pregnancy BMI, maternal education, marital status, wealth index, smoking, alcohol, parity, gravidity, hypertension	10
UHAS-AHPI	Yeboah 2017	Age, pre-pregnancy BMI, hypertension, diabetes	4
UMan-MatHealth	Ayoola 2012	Age, pre-pregnancy BMI, maternal education, marital status, gravidity, malaria, hypertension	7
USM-PregCohort	Loy 2014	Age, pre-pregnancy BMI, maternal education, marital status, hypertension, diabetes	6
USP-MatStress	Rondó 2003	Age, pre-pregnancy BMI, maternal education, marital status, smoking, alcohol, gravidity, hypertension, diabetes	9
UZ-MatNutri	Friis 2004	Age, pre-pregnancy BMI, parity, gravidity, malaria	5
WomenFirst	Hambidge 2019	Pre-pregnancy BMI, maternal education, parity, malaria, hypertension, diabetes	6
XJU-RuralChina	Zeng 2008	Age, pre-pregnancy BMI, maternal education, wealth index, gravidity, hypertension	6
XJU-Tibet	Kang 2017	Age, pre-pregnancy BMI, maternal education, wealth index, gravidity, hypertension	6

Supplementary Table 2: Summary of study characteristics.

Study acronym	Author, publication year	Country	Study type	Sample size	Enrolment eligibility criteria for GA or mean GA at enrolment in weeks ± SD	Maternal pre-pregnancy body-mass-index (BMI), kg/m^2			
						Underweight (<18.5)	Normal weight (18.5 to <25)	Overweight (25 to <30)	Obese (≥ 30)
AKU-MMN	Bhutta 2009	Pakistan	Intervention	1560	<16 wks	327 (21.0)	995 (64.8)	187 (12.0)	51 (3.27)
AKU-VITD	Khan 2016	Pakistan	Intervention	545	12 to 16 wks	54 (9.91)	372 (68.3)	90 (16.5)	29 (5.32)
Arg GWG Curves	Calvo 2009	Argentina	Cohort	1090	<16 wks	81 (7.43)	667 (61.2)	232 (21.3)	110 (10.1)
EU-MMN	Ramakrishnan 2003	Mexico	Intervention	457	≤13 wks	9 (1.97)	277 (60.6)	136 (28.8)	35 (7.66)
FU-GWG	Rodrigues 2010	Brazil	Cohort	176	8 to 13 wks	9 (5.11)	115 (65.3)	30 (17.1)	22 (12.5)
FU-LEPTINGWG	Franco-Sena 2016	Brazil	Cohort	275	5 to 13 wks	15 (5.45)	162 (58.9)	65 (23.6)	33 (12.0)
HERO-G	Moore 2020	The Gambia	Cohort	249	<28 weeks	41 (16.5)	169 (67.9)	25 (10.0)	14 (5.62)
HUST-TMCHC	Zhong 2019	China	Cohort	7329	<16 wks	1451 (19.8)	5317 (72.6)	504 (6.88)	57 (0.78)
ICDDR- MINIMat	Tofail 2003	Bangladesh	Intervention	3560	8 to 17 wks	886 (24.9)	2464 (69.2)	199 (5.59)	11 (0.31)
ILINS-DYAD-G	Adu-Afarwuah 2015	Ghana	Intervention	1186	≤20 wks	64 (5.40)	722 (60.9)	279 (23.5)	121 (10.2)
ILINS-DYAD-M	Ashorn 2015	Malawi	Intervention	1167	≤20 wks	90 (7.71)	970 (83.1)	89 (7.63)	18 (1.54)
IMIP-BRAMAG	de Araújo 2020	Brazil	Intervention	928	12 to 20 wks	32 (3.45)	436 (47.0)	289 (31.1)	171 (18.4)
IMIP-GestDM	do Nascimento 2019	Brazil	Cohort	518	<20 wks	14 (2.70)	261 (50.4)	172 (33.2)	71 (13.7)
INPer-CAR	Unpublished	Mexico	Cohort	408	None; 38.4 ± 2.0	18 (4.41)	309 (75.7)	63 (15.4)	18 (4.41)
INPer-FICA	Sámano 2017	Mexico	Cohort	168	26 to 28 wks	0 (0)	135 (80.4)	26 (15.5)	7 (4.17)
INPer-GDM	Unpublished	Mexico	Cohort	215	None: 12.7 ± 2.8	8 (3.72)	117 (54.4)	57 (26.5)	33 (15.4)
INPer-NeuroObesity	Unpublished	Mexico	Cohort	309	None: 13.5 ± 2.2	4 (1.29)	114 (36.9)	111 (35.9)	80 (25.9)
INPer-Poli	Unpublished	Mexico	Cohort	164	None; 24.8 ± 7.6	1 (0.61)	124 (75.6)	29 (17.7)	10 (6.10)
INPer-REDES	Sámano 2018	Mexico	Cohort	335	None; 38.4 ± 1.8	5 (1.49)	269 (80.3)	54 (16.1)	7 (2.09)
IRD-RECIPAL	Accrombessi 2018	Benin	Cohort	254	Preconception; 6.9 ± 2.5 wks	20 (7.87)	175 (68.9)	40 (15.8)	19 (7.48)
JHU-MothersGift	Tielsch 2015	Nepal	Intervention	3246	17 to 34 wks	428 (13.2)	2538 (78.2)	246 (7.58)	34 (1.05)
JiVitA3	West 2014	Bangladesh	Intervention	24,047	Preconception; 10.7 ± 3.8 wks	7296 (30.3)	16,170 (67.2)	550 (2.29)	31 (0.13)
LAIS	Hallamaa 2019	Malawi	Intervention	1148	28 to 34 wks	129 (11.2)	989 (86.2)	30 (2.61)	0 (0)
LCSS	Espo 2002	Malawi	Cohort	489	None; 35.4 ± 4.5	80 (16.4)	403 (82.4)	6 (1.23)	0 (0)
MRCG-ENID	Moore 2012	The Gambia	Intervention	836	Preconception; 13.7 ± 3.3 wks	153 (18.3)	403 (82.4)	6 (1.23)	0 (0)
MAHE-SCFPPP	Ramachandra 2017	India	Cohort	70	≤12 wks	9 (12.9)	46 (65.7)	14 (20.0)	1 (1.43)
MAL1	Etheredge 2015	Tanzania	Intervention	1402	≤27 wks	99 (7.06)	878 (62.6)	312 (22.3)	113 (8.06)
MAL2	Darling 2017	Tanzania	Intervention	2128	≤13 wks	196 (9.21)	1344 (63.2)	417 (19.6)	171 (8.04)
MDIG	Roth 2018	Bangladesh	Intervention	1283	17 to 24 wks	113 (8.81)	863 (67.3)	265 (20.7)	42 (3.27)
MINA-Brazil	Cardoso 2020	Brazil	Cohort	1326	<20 wks	57 (4.30)	805 (60.7)	343 (25.9)	121 (9.13)
MIRA-Janakpur	Osrin 2005	Nepal	Intervention	1132	≤20 wks	291 (25.7)	819 (72.4)	20 (1.77)	2 (0.18)
MISAME-1	Roberfroid 2008	Burkina Faso	Intervention	1156	None; 16.0 ± 6.2	185 (16.0)	948 (82.0)	21 (1.82)	2 (0.17)
MISAME-2	Huybregts 2009	Burkina Faso	Intervention	1186	None; 15.8 ± 6.1	196 (16.6)	954 (80.6)	34 (2.87)	0 (0)
NNIPS-3	Christian 2003	Nepal	Intervention	2952	Preconception; 10.2 ± 3.6 wks	990 (33.5)	1936 (65.6)	26 (0.88)	0 (0)
NWU-PMPEN	Widen 2019	Kenya	Cohort	101	<30 wks	8 (7.92)	63 (62.4)	19 (18.8)	11 (10.9)
NWU-PreNAPS	Widen 2017	Uganda	Cohort	159	10 to 26 wks	10 (6.29)	130 (81.8)	17 (10.7)	2 (1.26)
PNS	Fawzi 2007	Tanzania	Intervention	7577	12 to 27 wks	482 (6.32)	5085 (67.1)	1577 (20.8)	433 (5.71)
ROSE	Isanaka 2019	Niger	Intervention	742	None: 19.3 ± 2.5 wks	57 (9.68)	473 (80.3)	49 (8.32)	10 (1.70)
SBUMS-GDM	Tehrani 2019	Iran	Cohort	26,199	<14 wks	1269 (4.84)	11,082 (42.3)	9470 (36.2)	4378 (16.7)
SHU-BMIGWG	Soltani 2017	Indonesia	Cohort	562	<14 wks	113 (20.1)	366 (65.1)	73 (13.0)	10 (1.78)
SPAZ-IPTp	Unger 2015	Papua New Guinea	Intervention	1981	<26 wks	196 (9.89)	1622 (81.2)	148 (7.47)	15 (0.76)

St-Johns	Unpublished	India	Cohort	2001	None: 11.9 ± 2.7	336 (16.8)	1292 (64.6)	323 (16.1)	50 (2.50)
TU-Aflatoxin	Lauer 2019	Uganda	Cohort	246	None: 17.8 ± 3.4	9 (3.66)	170 (69.1)	55 (22.4)	12 (4.88)
UC-RDNS	Matias 2016	Bangladesh	Intervention	3819	≤20 wks	784 (20.5)	2850 (74.6)	178 (4.66)	7 (0.18)
UCL-LBWSAT	Saville 2018	Nepal	Intervention	2820	None: 20.6 ± 4.8	539 (19.1)	2220 (78.7)	57 (2.02)	4 (0.14)
UHAS-AHPI	Yeboah 2017	Ghana	Cohort	290	None; 12 ± 0	7 (2.41)	138 (47.6)	100 (34.8)	45 (15.5)
UMan-MatHealth	Ayoola 2012	Nigeria	Cohort	347	<36 wks	33 (9.48)	215 (61.8)	74 (21.3)	26 (7.47)
USM-PregCohort	Loy 2014	Malaysia	Cohort	153	≤24 wks	23 (15.0)	91 (59.5)	31 (20.3)	8 (5.23)
USP-MatStress	Rondó 2003	Brazil	Cohort	924	<16 wks	28 (3.03)	654 (70.8)	188 (20.4)	54 (5.84)
UZ-MatNutri	Friis 2004	Zimbabwe	Intervention	275	22 to 36 wks	7 (2.55)	210 (76.4)	46 (16.7)	12 (4.36)
WomenFirst	Hambidge 2019	Guatemala, India, and Pakistan	Intervention	1984	Preconception; 9.5 ± 5.6 wks	455 (22.9)	1121 (56.5)	305 (15.4)	103 (5.19)
XJU-RuralChina	Zeng 2008	China	Intervention	4578	<28 wks	769 (16.8)	3719 (81.2)	80 (1.75)	10 (0.22)
XJU-Tibet	Kang 2017	China	Intervention	1039	≤24 wks	267 (25.7)	734 (70.6)	37 (3.56)	1 (0.10)

[1] A value of 0 indicates no observation for the given category in a study.

Supplementary Table 3: Study characteristics and maternal pre-pregnancy body mass index and total gestational weight gain adequacy based on the Institute of Medicine recommendations.

Study acronym	Author, publication year	Country	Gestational weight gain adequacy, n (%)			
			Severely inadequate (<70%)	Moderately inadequate (70 to <90%)	Adequate (90% - <125%)	Excessive (≥125%)
AKU-MMN	Bhutta 2009	Pakistan	781 (50.8)	433 (28.1)	211 (13.7)	114 (7.41)
AKU-VITD	Khan 2016	Pakistan	139 (25.6)	123 (22.6)	150 (27.6)	132 (24.3)
Arg GWG Curves	Calvo 2009	Argentina	207 (19.0)	130 (12.0)	294 (27.0)	457 (42.0)
EU-MMN	Ramakrishnan 2003	Mexico	89 (24.7)	61 (16.9)	127 (35.2)	84 (23.3)
FU-GWG	Rodrigues 2010	Brazil	24 (16.0)	15 (10.0)	41 (27.3)	70 (0.26)
FU-LEPTINGWG	Franco-Sena 2016	Brazil	41 (17.2)	24 (10.1)	57 (24.0)	116 (48.7)
HERO-G	Moore 2020	The Gambia	98 (39.4)	54 (21.7)	65 (26.1)	32 (12.9)
HUST-TMCHC	Zhong 2019	China	395 (5.39)	812 (11.1)	2636 (36.0)	3482 (47.5)
ICDDRB-MINIMat	Tofail 2008	Bangladesh	2084 (58.6)	769 (21.6)	566 (15.9)	135 (3.80)
ILINS-DYAD-G	Adu-Afarwuah 2015	Ghana	288 (25.4)	175 (15.4)	326 (28.8)	345 (30.4)
ILINS-DYAD-M	Ashorn 2015	Malawi	422 (36.6)	337 (29.2)	302 (26.2)	93 (8.06)
IMIP-BRAMAG	de Araújo 2020	Brazil	84 (9.50)	118 (13.4)	232 (26.2)	450 (50.9)
IMIP-GestDM	do Nascimento 2019	Brazil	128 (24.7)	135 (26.1)	121 (23.4)	134 (25.9)
INPer-CAR	Unpublished	Mexico	94 (23.0)	58 (14.2)	104 (25.5)	152 (37.3)
INPer-FICA	Sámano 2017	Mexico	25 (14.9)	31 (18.5)	47 (28.0)	65 (38.7)
INPer-GDM	Unpublished	Mexico	51 (23.7)	43 (20.0)	45 (20.9)	76 (35.4)
INPer-NeuroObesity	Unpublished	Mexico	97 (34.6)	24 (8.57)	46 (16.4)	113 (40.4)
INPer-Poli	Unpublished	Mexico	43 (26.2)	26 (15.9)	41 (25.0)	54 (32.9)
INPer-REDES	Sámano 2018	Mexico	59 (17.6)	51 (15.2)	109 (32.5)	116 (34.6)
IRD-RECIPAL	Accrombessi 2018	Benin	103 (40.6)	45 (17.7)	67 (26.4)	39 (15.4)
JHU-MothersGift	Tielsch 2015	Nepal	2317 (72.8)	484 (15.2)	312 (9.81)	69 (2.17)
JiVitA3	West 2014	Bangladesh	15,106 (62.8)	5,276 (21.9)	3,133 (13.0)	532 (2.21)
LAIS	Hallamaa 2019	Malawi	575 (50.1)	350 (30.5)	193 (16.8)	67 (8.12)
LCSS	Espo 2002	Malawi	269 (55.1)	145 (29.7)	67 (13.7)	7 (1.43)
MRCG-ENID	Moore 2012	The Gambia	474 (57.5)	148 (17.9)	136 (16.5)	67 (8.12)
MAHE-SCFPPP	Ramachandra 2017	India	6 (8.57)	9 (12.9)	19 (27.1)	36 (51.4)
MAL1	Etheredge 2015	Tanzania	298 (21.8)	299 (21.9)	360 (26.3)	410 (30.0)
MAL2	Darling 2017	Tanzania	653 (34.8)	272 (14.5)	461 (24.6)	489 (26.1)
MDIG	Roth 2018	Bangladesh	349 (27.2)	303 (23.6)	361 (28.1)	270 (21.0)
MINA-Brazil	Cardoso 2020	Brazil	264 (19.9)	197 (14.9)	385 (29.0)	480 (36.2)
MIRA-Janakpur	Osrin 2005	Nepal	574 (50.7)	297 (26.2)	298 (11.7)	85 (3.33)
MISAME-1	Roberfroid 2008	Burkina Faso	716 (64.3)	255 (22.9)	105 (9.43)	37 (3.32)
MISAME-2	Huybregts 2009	Burkina Faso	733 (64.8)	224 (19.8)	142 (12.5)	33 (2.92)
NNIPS-3	Christian 2003	Nepal	2,013 (68.2)	547 (18.5)	335 (11.4)	57 (1.93)
NWU-PMPEN	Widen 2019	Kenya	24 (23.8)	28 (27.7)	23 (22.8)	26 (25.7)
NWU-PreNAPS	Widen 2017	Uganda	66 (41.5)	46 (28.9)	34 (21.4)	13 (8.18)
PNS	Fawzi 2007	Tanzania	1889 (24.9)	2125 (28.1)	2117 (28.0)	1442 (19.0)
ROSE	Isanaka 2019	Niger	1137 (52.1)	478 (21.9)	408 (18.7)	158 (7.24)
SBUMS-GDM	Tehrani 2019	Iran	3072 (11.7)	2977 (11.4)	6,952 (26.5)	13,198 (50.4)
SHU-BMIGWG	Soltani 2017	Indonesia	178 (32.7)	95 (17.4)	140 (25.7)	132 (24.2)
SPAZ-IPTp	Unger 2015	Papua New Guinea	339 (17.4)	424 (21.8)	746 (38.4)	436 (22.4)
St-Johns	Unpublished	India	281 (17.7)	270 (17.0)	504 (31.7)	534 (33.6)
TU-Aflatoxin	Lauer 2019	Uganda	85 (35.0)	61 (25.1)	61 (25.1)	36 (14.8)
UC-RDNS	Matias 2016	Bangladesh	1,906 (55.9)	840 (24.6)	530 (15.5)	137 (4.01)
UCL-LBWSAT	Saville 2018	Nepal	1372 (53.8)	795 (31.2)	298 (11.7)	85 (3.33)
UHAS-AHPI	Yeboah 2017	Ghana	32 (11.0)	27 (9.31)	90 (31.0)	141 (48.6)
UMan-MatHealth	Ayoola 2012	Nigeria	112 (32.5)	83 (24.1)	83 (24.1)	67 (19.4)

USM-PregCohort	Loy 2014	Malaysia	42 (27.5)	34 (22.2)	32 (20.9)	45 (29.4)
USP-MatStress	Rondó 2003	Brazil	145 (15.8)	171 (18.6)	301 (32.8)	301 (32.8)
UZ-MatNutri	Friis 2004	Zimbabwe	23 (8.36)	152 (55.3)	47 (17.1)	53 (19.3)
WomenFirst	Hambidge 2019	Guatemala, India, and Pakistan	1022 (52.3)	405 (20.7)	336 (17.2)	192 (9.82)
XJU-RuralChina	Zeng 2008	China	1552 (36.6)	788 (18.6)	1145 (27.0)	751 (17.7)
XJU-Tibet	Kang 2017	China	310 (30.0)	250 (24.2)	299 (28.9)	176 (17.0)

Supplementary Table 4: Risk of bias using the Quality In Prognosis Studies (QUIPS) tool.

Study acronym	Author, publication year	Country	Domain of biases for assessment			
			Study participation	Study attrition	Measurement of maternal weight	Measurement of neonatal outcomes
AKU-MMN	Bhutta 2009	Pakistan	Low	Low	Low	Low
AKU-VITD	Khan 2016	Pakistan	Moderate	Moderate	Low	Low
Arg GWG Curves	Calvo 2009	Argentina	Low	Low	Low	Low
EU-MMN	Ramakrishnan 2003	Mexico	Low	Low	Low	Low
FU-GWG	Rodrigues 2010	Brazil	Low	Low	Low	Low
FU-LEPTINGWG	Franco-Sena 2016	Brazil	Low	Moderate	Low	Low
HERO-G	Moore 2020	The Gambia	Low	Low	Low	Low
HUST-TMCHC	Zhong 2019	China	Low	Moderate	Low	Low
ICDDRB-MINIMat	Tofail 2008	Bangladesh	Low	Low	Low	Low
ILINS-DYAD-G	Adu-Afarwuah 2015	Ghana	Low	Low	Low	Low
ILINS-DYAD-M	Ashorn 2015	Malawi	Low	Low	Low	Low
IMIP-BRAMAG	de Araújo 2020	Brazil	Moderate	Low	Low	Low
IMIP-GestDM	do Nascimento 2019	Brazil	Moderate	Moderate	Low	Low
INPer-CAR	Unpublished*	Mexico	-	-	Low	Low
INPer-FICA	Sámano 2017	Mexico	Low	Moderate	Low	Low
INPer-GDM	Unpublished*	Mexico	-	-	Low	Low
INPer-NeuroObesity	Unpublished*	Mexico	-	-	Low	Low
INPer-Poli	Unpublished*	Mexico	-	-	Low	Low
INPer-REDES	Sámano 2018	Mexico	Low	Low	Low	Low
IRD-RECIPAL	Accrombessi 2018	Benin	Low	Low	Low	Low
JHU-MothersGift	Tielsch 2015	Nepal	Low	Low	Low	Low
JiVitA3	West 2014	Bangladesh	Low	Low	Low	Low
LAIS	Hallamaa 2019	Malawi	Moderate	Low	Low	Low
LCSS	Espo 2002	Malawi	Low	Low	Low	Moderate
MRCG-ENID	Moore 2012	The Gambia	Low	Low	Low	Low
MAHE-SCFPPP	Ramachandra 2017	India	Low	Moderate	Low	Low
MAL1	Etheredge 2015	Tanzania	Low	Low	Low	Low
MAL2	Darling 2017	Tanzania	Low	Low	Low	Low
MDIG	Roth 2018	Bangladesh	Low	Moderate	Low	Low
MINA-Brazil	Cardoso 2020	Brazil	Low	Low	Low	Low
MIRA-Janakpur	Osrin 2005	Nepal	Low	Moderate	Low	Low
MISAME-1	Roberfroid 2008	Burkina Faso	Low	Low	Low	Low
MISAME-2	Huybregts 2009	Burkina Faso	Low	Moderate	Low	Low
NNIPS-3	Christian 2003	Nepal	Low	Moderate	Low	Low
NWU-PMPEN	Widen 2019	Kenya	Low	Low	Low	Moderate
NWU-PreNAPS	Widen 2017	Uganda	Low	Low	Low	Low
PNS	Fawzi 2007	Tanzania	Low	Low	Low	Low
ROSE	Isanaka 2019	Niger	Moderate	Moderate	Low	Low
SBUMS-GDM	Tehrani 2019	Iran	Moderate	Low	Low	Low
SHU-BMIGWG	Soltani 2017	Indonesia	Low	Moderate	Low	Low
SPAZ-IPTp	Unger 2015	Papua New Guinea	Moderate	Moderate	Low	Low
St-Johns	Unpublished*	India	-	-	Low	Low
TU-Aflatoxin	Lauer 2019	Uganda	Moderate	Low	Low	Low
UC-RDNS	Matias 2016	Bangladesh	Low	Moderate	Low	Low
UCL-LBWSAT	Saville 2018	Nepal	Low	High	Low	Low
UHAS-AHPI	Yeboah 2017	Ghana	High	Moderate	Low	Moderate
UMan-MatHealth	Ayoola 2012	Nigeria	Moderate	Low	Low	Low
USM-PregCohort	Loy 2014	Malaysia	Moderate	Moderate	Low	Low

USP-MatStress	Rondó 2003	Brazil	Moderate	Low	Low	Low
UZ-MatNutri	Friis 2004	Zimbabwe	Low	Low	Low	Low
WomenFirst	Hambidge 2019	Guatemala, India, and Pakistan	Low	Low	Low	Low
XJU-RuralChina	Zeng 2008	China	Low	Moderate	Low	Low
XJU-Tibet	Kang 2017	China	Moderate	Low	Low	Low
		Overall rating per domain	***Low***	***Moderate***	***Low***	***Low***

*Investigators contributed data from additional unpublished studies when they were contacted to contribute data for the GWG Pooling Project to based on a published study. These additional studies were relevant for the research question for the pooled analysis and met all study inclusion criteria.

Supplementary Table 5: Summary of newborn neonatal outcomes among included studies[1].

Study	Female sex n (%)	Stillbirth, n (%)	Neonatal death n (%)	Preterm birth n (%)	Low birthweight (<2500 grams) n (%)	Macrosomia (>4000 grams) n (%)	Anthropometric outcomes, n (%)			
							Small-for-gestational age (SGA)	Large-for-gestational age (LGA)	Short-for-gestational age	Microcephaly
AKU-MMN	713 (45.7)	52 (3.33)	57 (3.65)	484 (31.0)	276 (17.7)	40 (2.56)	447 (28.7)	309 (19.8)	187 (12.0)	122 (7.82)
AKU-VITD	213 (39.1)	4 (0.73)	0 (0)	127 (23.3)	66 (12.1)	7 (1.28)	79 (14.5)	36 (6.61)	1 (0.18)	-
Arg GWG Curves	562 (51.6)	0 (0)	-	62 (5.69)	55 (5.05)	51 (4.68)	63 (5.78)	148 (13.6)	205 (18.8)	49 (4.50)
EU-MMN	217 (47.5)	4 (0.88)	4 (0.88)	13 (2.84)	30 (6.56)	0 (0)	121 (26.5)	6 (1.31)	20 (4.38)	5 (1.09)
FU-GWG	78 (44.3)	0 (0)	-	18 (10.2)	11 (6.25)	11 (6.25)	8 (4.55)	25 (14.2)	-	-
FU-LEPTINGWG	118 (42.9)	3 (1.09)	-	32 (11.6)	17 (6.18)	15 (5.45)	29 (10.6)	43 (15.6)	19 (6.91)	-
HERO-G	111 (44.6)	7 (2.81)	4 (1.61)	9 (3.61)	11 (4.42)	0 (0)	36 (14.5)	0 (0)	11 (4.42)	45 (18.1)
HUST-TMCHC	3275 (46.8)	0 (0)	-	330 (4.50)	199 (2.72)	350 (4.78)	343 (4.68)	889 (12.1)	26 (0.35)	-
ICDDRB-MINIMat	1734 (48.7)	25 (0.70)	32 (0.90)	188 (5.28)	990 (27.8)	3 (0.08)	2122 (59.6)	6 (0.17)	675 (19.0)	1038 (29.2)
ILINS-DYAD-G	569 (48.0)	27 (2.28)	13 (1.10)	98 (8.26)	86 (7.25)	10 (0.84)	152 (12.8)	92 (7.76)	37 (3.12)	18 (1.52)
ILINS-DYAD-M	541 (46.4)	10 (0.86)	36 (3.08)	86 (7.37)	119 (10.2)	28 (2.40)	263 (22.5)	45 (3.86)	-	30 (2.57)
IMIP-BRAMAG	382 (41.2)	0 (0)	-	68 (7.33)	52 (5.60)	45 (4.85)	52 (5.60)	132 (14.2)	52 (5.60)	-
IMIP-GestDM	189 (36.5)	0 (0)	-	30 (5.79)	29 (5.60)	22 (4.25)	30 (5.79)	59 (11.4)	19 (3.67)	6 (1.16)
INPer-CAR	190 (46.6)	0 (0)	0 (0)	49 (12.0)	57 (14.0)	1 (0.25)	70 (17.2)	17 (4.17)	12 (2.94)	-
INPer-FICA	104 (61.9)	0 (0)	0 (0)	0	10 (5.95)	0	30 (17.9)	9 (5.36)	1 (0.60)	-
INPer-GDM	93 (43.3)	0 (0)	-	41 (19.1)	31 (14.4)	1 (0.47)	25 (11.6)	12 (5.58)	37 (17.2)	-
INPer-NeuroObesity	131 (42.4)	0 (0)	2 (0.65)	34 (11.0)	52 (16.8)	3 (0.97)	73 (23.6)	11 (3.56)	38 (12.3)	8 (2.59)
INPer-Poli	68 (48.6)	0 (0)	0 (0)	18 (11.0)	25 (15.2)	1 (0.61)	21 (12.8)	5 (3.05)	7 (4.27)	-
INPer-REDES	176 (52.5)	0 (0)	0 (0)	36 (10.8)	58 (17.3)	0 (0)	67 (20.0)	4 (1.19)	19 (5.67)	35 (10.5)
IRD-RECIPAL	120 (47.2)	3 (1.18)	-	35 (13.8)	23 (9.06)	1 (0.39)	62 (24.4)	21 (8.27)	27 (10.6)	17 (6.69)
JHU-MothersGift	1516 (46.7)	54 (1.66)	25 (0.77)	421 (13.0)	651 (20.1)	114 (3.51)	1172 (36.1)	238 (7.33)	444 (13.7)	506 (15.6)
JiVitA3	11,752 (48.9)	0 (0)	653 (2.72)	3742 (15.6)	7681 (31.9)	12 (0.05)	11,212 (46.6)	386 (1.61)	5283 (22.0)	3307 (13.8)
LAIS	548 (47.7)	0 (0)	69 (6.01)	158 (13.8)	79 (6.88)	12 (1.05)	163 (14.2)	43 (3.75)	-	18 (1.57)
LCSS	240 (49.1)	0 (0)	-	92 (18.8)	8 (1.64)	0 (0)	21 (4.29)	6 (1.23)	22 (4.50)	-
MRCG-ENID	398 (47.6)	21 (2.51)	12 (1.44)	42 (5.02)	66 (7.89)	3 (0.36)	229 (27.4)	19 (2.27)	25 (2.99)	105 (12.6)
MAHE-SCFPPP	33 (47.1)	0 (0)	0 (0)	0	6 (8.57)	0 (0)	23 (32.9)	3 (4.29)	-	-
MAL1	668 (47.7)	54 (3.85)	3 (0.21)	225 (16.1)	104 (7.42)	40 (2.85)	265 (18.9)	190 (13.6)	-	-
MAL2	1163 (54.7)	6 (0.28)	65 (3.05)	426 (20.0)	127 (5.97)	35 (1.64)	338 (15.9)	265 (12.5)	-	-
MDIG	621 (48.4)	29 (2.26)	15 (1.17)	124 (9.66)	230 (17.9)	0 (0)	397 (30.9)	3 (0.23)	109 (8.50)	68 (5.30)
MINA-Brazil	669 (50.5)	0 (0)	0 (0)	96 (7.24)	86 (6.49)	82 (6.18)	118 (8.90)	158 (11.9)	34 (2.56)	75 (5.66)
MIRA-Janakpur	559 (49.4)	33 (2.92)	28 (2.47)	93 (8.22)	224 (19.8)	5 (0.44)	455 (40.2)	11 (0.97)	87 (7.69)	65 (5.74)
MISAME-1	522 (45.2)	25 (2.16)	-	180 (15.7)	139 (12.0)	3 (0.26)	263 (22.8)	51 (4.41)	95 (8.22)	41 (3.55)
MISAME-2	503 (42.5)	26 (2.20)	-	200 (16.9)	131 (11.1)	5 (0.42)	226 (19.1)	42 (3.55)	123 (10.4)	75 (6.33)
NNIPS-3	1406 (47.6)	93 (3.15)	75 (2.54)	478 (16.2)	936 (31.7)	2 (0.07)	1411 (47.8)	91 (3.08)	539 (18.3)	509 (17.2)
NWU-PMPEN	55 (54.6)	0 (0)	-	29 (28.7)	3 (2.97)	8 (7.92)	4 (3.96)	33 (32.7)	-	-
NWU-PreNAPS	74 (46.5)	0 (0)	-	22 (13.8)	5 (3.14)	13 (8.18)	14 (8.81)	32 (20.1)	5 (3.14)	1 (0.63)
PNS	3593 (47.4)	264 (3.48)	182 (2.40)	1380 (18.2)	473 (6.24)	177 (2.34)	1385 (18.3)	927 (12.2)	1321 (17.4)	504 (6.65)
ROSE	1078 (49.4)	4 (0.71)	1 (0.05)	883 (40.5)	175 (8.02)	58 (2.66)	297 (13.6)	702 (32.7)	167 (7.65)	46 (2.11)
SBUMS-GDM	13,309 (50.8)	181 (0.69)	-	1762 (6.73)	1371 (5.23)	814 (3.11)	2407 (9.19)	2950 (11.3)	657 (2.51)	507 (1.94)
SHU-BMIGWG	-	4 (0.71)	-	50 (8.90)	15 (2.67)	9 (1.60)	-	-	-	-
SPAZ-IPTp	1068 (53.9)	34 (1.72)	22 (1.11)	352 (17.8)	301 (15.2)	19 (0.96)	473 (23.9)	174 (8.78)	412 (20.8)	336 (17.0)
St-Johns	881 (44.0)	51 (2.55)	-	328 (16.4)	323 (16.1)	11 (0.55)	507 (25.3)	40 (2.00)	26 (1.30)	31 (1.55)
TU-Aflatoxin	120 (48.8)	4 (1.63)	3 (1.22)	19 (7.72)	8 (3.25)	7 (2.85)	25 (10.2)	19 (7.72)	26 (10.6)	5 (2.03)
UC-RDNS	1785 (46.7)	97 (2.54)	89 (2.33)	620 (16.2)	1285 (33.7)	3 (0.08)	1996 (52.3)	138 (3.61)	626 (16.4)	575 (15.1)
UCL-LBWSAT	1306 (46.3)	52 (1.84)	74 (2.62)	262 (9.29)	204 (7.23)	34 (1.21)	384 (13.6)	133 (4.72)	151 (5.35)	77 (2.73)

UHAS-AHPI	144 (49.7)	8 (2.76)	-	206 (71.0)	14 (4.83)	9 (3.10)	11 (3.79)	144 (49.7)	-	-
UMan-MatHealth	121 (34.8)	5 (1.44)	5 (1.44)	44 (12.6)	35 (10.1)	2 (0.57)	74 (21.3)	20 (5.75)	19 (5.46)	3 (0.86)
USM-PregCohort	84 (54.9)	0 (0)	0 (0)	0	11 (7.19)	4 (2.61)	25 (16.3)	7 (4.58)	8 (5.23)	7 (4.58)
USP-MatStress	486 (52.6)	2 (0.22)	-	50 (5.41)	58 (6.28)	43 (4.65)	85 (9.20)	91 (9.85)	88 (9.52)	36 (3.90)
UZ-MatNutri	130 (47.3)	4 (1.45)	1 (0.36)	45 (16.4)	21 (7.64)	6 (2.18)	54 (19.6)	28 (10.2)	15 (5.45)	19 (6.91)
WomenFirst	990 (49.9)	56 (2.82)	40 (2.02)	285 (14.4)	465 (23.4)	2 (0.10)	731 (36.8)	85 (4.28)	282 (14.2)	206 (10.4)
XJU-RuralChina	2032 (44.4)	157 (3.43)	65 (1.42)	268 (5.85)	170 (3.69)	62 (1.33)	707 (15.4)	276 (6.03)	531 (11.6)	622 (13.6)
XJU-Tibet	518 (49.9)	13 (1.25)	23 (2.21)	205 (19.7)	84 (8.08)	10 (0.96)	309 (29.7)	126 (12.1)	221 (21.3)	187 (18.0)

[1] A value of 0 indicates no cases of a given neonatal outcome as reported by the investigator, where as a dash indicates no available data for that given outcome in a study.

Supplementary Table 6: Adjusted associations between gestational weight gain (GWG) adequacy and neonatal outcomes among women with last weight measure in the third trimester using two stage meta-analysis.[1]

Characteristic	No. of studies	No. of subjects	Severely inadequate (<70%)			Moderately inadequate (70 to <90%)			Excessive (≥125%)		
			RR[2] (95% CI)	I^2 (%)	Tau^2	RR[2] (95% CI)	I^2 (%)	Tau^2	RR[2] (95% CI)	I^2 (%)	Tau^2
Preterm birth	45	96,308	1.06 (0.95, 1.18)	68	0.059	1.02 (0.94, 1.11)	37	0.018	1.22 (1.13, 1.31)	16	0.008
Low birthweight	47	87,976	1.62 (1.51, 1.73)	15	0.006	1.26 (1.20, 1.33)	0	0	0.92 (0.83, 1.02)	20	0.017
Small-for-gestational age	48	87,687	1.45 (1.36, 1.54)	57	0.016	1.22 (1.18, 1.26)	0	0	0.79 (0.75, 0.83)	0	0
Large-for-gestational age	41	83,678	0.65 (0.57, 0.75)	58	0.070	0.82 (0.76, 0.88)	0	0	1.45 (1.33, 1.59)	24	0.011
Stillbirth	18	52,838	1.08 (0.87, 1.34)	17	0.035	1.04 (0.86, 1.26)	0	0	1.09 (0.87, 1.37)	0	0
Neonatal death	19	52,285	0.90 (0.71, 1.14)	37	0.073	0.84 (0.71, 1.01)	0	0	1.06 (0.77, 1.42)	15	0.030
Short-for-gestational age	40	80,022	1.47 (1.29, 1.69)	68	0.074	1.20 (1.11, 1.31)	20	0.009	0.89 (0.81, 0.99)	11	0.007
Microcephaly	30	76,302	1.57 (1.31, 1.88)	78	0.145	1.26 (1.12, 1.41)	38	0.026	0.88 (0.79, 0.99)	4.25	0.003
Macrosomia	25	61,758	0.59 (0.49, 0.72)	0	0	0.63 (0.51, 0.77)	0	0	1.50 (1.28, 1.76)	7.65	0.010

[1] Multivariable models adjusted for maternal age, maternal pre-pregnancy BMI, and study intervention arm (if applicable) as well as other important confounders as available in each study. Reference category: adequate GWG (90 to <125%). For stillbirth, neonatal death, and macrosomia, measures of association with GWG adequacy were estimated using odds ratio given very low prevalence of these outcomes and convergence issues with modified Poisson regression. Odds ratios approximate relative risk in the context of rare outcomes.

Supplementary Table 7: Adjusted associations between gestational weight gain (GWG) adequacy using the lower rate of weight gain for the second and third trimesters based on Institute of Medicine guidelines and neonatal outcomes using two stage meta-analysis.[1]

Neonatal outcomes	No. of studies	No. of subjects	Severely inadequate GWG (<70%)			Moderately inadequate GWG (70 to <90%)			Excessive GWG (≥125%)		
			RR^2 (95% CI)	I^2 (%)	Tau^2	RR^2 (95% CI)	I^2 (%)	Tau^2	RR^2 (95% CI)	I^2 (%)	Tau^2
Preterm birth	50	104,203	1.08 (0.97, 1.20)	68	0.059	0.91 (0.85, 0.97)	13	0.005	1.14 (1.05, 1.32)	40	0.019
Low birthweight	47	93,188	1.48 (1.40, 1.57)	20	0.005	1.18 (1.13, 1.23)	0	0	0.80 (0.76, 0.85)	0	0
Small-for-gestational age	51	93,531	1.33 (1.26, 1.40)	57	0.011	1.17 (1.12, 1.23)	31	0.004	0.75 (0.72, 0.78)	13	0.003
Large-for-gestational age	42	85,294	0.75 (0.63, 0.89)	68	0.125	0.86 (0.77, 0.97)	29	0.023	1.56 (1.43, 1.71)	27	0.016
Stillbirth	20	62,111	1.19 (0.93, 1.53)	41	0.099	1.00 (0.82, 1.22)	0	0	1.19 (1.01, 1.41)	0	0
Neonatal death	18	56,554	1.21 (1.05, 1.39)	0	0	1.12 (0.96, 1.32)	0	0	1.26 (1.07, 1.49)	0	0
Short-for-gestational age	39	83,610	1.41 (1.26, 1.57)	60	0.037	1.23 (1.15, 1.30)	5.92	0.002	0.81 (0.75, 0.87)	8.30	0.004
Microcephaly	30	79,270	1.51 (1.32, 1.72)	68	0.056	1.17 (1.06, 1.29)	29	0.015	0.76 (0.67, 0.86)	48	0.039
Macrosomia	27	64,505	0.87 (0.59, 1.27)	46	0.175	0.87 (0.68, 1.12)	0	0	1.80 (1.55, 2.08)	0	0

[1] Multivariable models adjusted for maternal age, maternal pre-pregnancy BMI, and study intervention arm (if applicable) as well as other important confounders as available in each study. Reference category: adequate GWG (90 to <125%). For stillbirth, neonatal death, and macrosomia, measures of association with GWG adequacy were estimated using odds ratio given very low prevalence of these outcomes and convergence issues with modified Poisson regression. Odds ratios approximate the relative risk in the context of rare outcomes.

Supplementary Table 8: Adjusted associations between gestational weight gain (GWG) adequacy based on the Institute of Medicine 2009 recommendations and perinatal outcomes using a one stage meta-analysis approach.[1]

Characteristic	n	Severely inadequate (<70%)	Moderately inadequate (70% to <90%)	Adequate (90% to <125%)	Excessive (≥125%)
		RR[2] (95% CI)	RR[2] (95% CI)	Reference	RR[2] (95% CI)
Preterm birth	111,464	1.01 (0.96, 1.06)	1.06 (1.00, 1.11)	1.0	1.12 (1.06, 1.19)
Low birthweight	100,535	1.67 (1.60, 1.74)	1.25 (1.19, 1.31)	1.0	0.95 (0.89, 1.02)
Small-for-gestational age	99,800	1.46 (1.42, 1.50)	1.20 (1.16, 1.24)	1.0	0.79 (0.75, 0.83)
Large-for-gestational age	98,102	0.66 (0.61, 0.71)	0.84 (0.77, 0.90)	1.0	1.58 (1.48, 1.68)
Stillbirth	74,549	1.12 (0.96, 1.32)	1.02 (0.86, 1.22)	1.0	1.04 (0.87, 1.26)
Neonatal death	63,957	0.99 (0.86, 1.14)	0.79 (0.67, 0.94)	1.0	1.06 (0.84, 1.33)
Short-for-gestational age	90,251	1.59 (1.51, 1.67)	1.15 (1.09, 1.22)	1.0	0.92 (0.85, 0.99)
Microcephaly	84,054	1.65 (1.56, 1.75)	1.19 (1.11, 1.28)	1.0	0.91 (0.82, 1.00)
Macrosomia	92,932	0.57 (0.48, 0.67)	0.63 (0.53, 0.75)	1.0	1.69 (1.50, 1.90)

[1] Multivariable modified Poisson models with robust variance adjusted for maternal age, maternal pre-pregnancy BMI, study intervention arm (if applicable), and study fixed effects using a one-stage meta-analysis approach. For stillbirth, neonatal death, and macrosomia, measures of association with GWG adequacy were estimated using odds ratio given very low prevalence of these outcomes and convergence issues with modified Poisson regression. Odds ratios approximate relative risk in the context of rare outcomes.

Supplementary text for interaction analyses:

We used a one stage modelling approach with two-way interaction terms between GWG adequacy and maternal pre-pregnancy BMI or maternal age. To prevent aggregation bias by conflating the within- and between-study heterogeneity in the one stage model, we used centered covariates for the exposure (i.e., GWG adequacy categories) and covariates (i.e., maternal pre-pregnancy BMI or maternal age categories) and used the variable means as additional covariates in the models as outlined in Riley et al., 2020. Notably, the coding approach outlined in Yaremych et al (2021) were used to generate study-specific centered dummy variables of GWG adequacy, maternal pre-pregnancy BMI, and maternal age categories. We used a centered variable for our primary exposure, GWG adequacy, because this IPD analyses included both randomized controlled trials and observational cohorts and, as such, the distribution of participants across strata of GWG adequacy is not randomized. Therefore, we applied a one-stage interaction model using centered variables to account for variability in distribution of exposure and covariate (i.e., effect modifier) distributions between studies, using the following equation:

$$logit\ (p_{ij}) = \alpha_i + \beta_{1i}(x_{ij} - \overline{x}_i)(z_{ij} - \overline{z}_i) + \beta_{2i}(x_{ij} - \overline{x}_i)(\overline{z}_i) + \beta_{3i}(z_{ij} - \overline{z}_i)(\overline{x}_i) + \beta_{4i}(x_{ij}) + \beta_{5i}(z_{ij}) + \varepsilon_{ij}$$

where x_{ij} is the GWG adequacy category; $\overline{x}_i$ is the study-specific mean GWG adequacy; z_{ij} is maternal pre-pregnancy BMI (or maternal age); and $\overline{z}_i$ is the study-specific mean of maternal pre-pregnancy BMI (or maternal age). Post-hoc linear combination of terms from the one stage model were used to estimate the relationship between GWG adequacy and a given neonatal outcome by strata of maternal pre-pregnancy BMI, using the overall proportion of women with underweight, overweight, or obese in the pooled database as values imputed in the one-stage model equation. A similar approach was used for the proportion of women with maternal age in the <20, 20-29, and ≥30 year categories. We opted to continue to use a one stage analysis as opposed to a two-stage approach for interaction analyses to leverage all available data that will allow an examination of effect modifiers of rare outcomes as well.

Supplementary Table 9: Associations between percent adequacy of total gestational weight gain and neonatal outcomes, modified by maternal pre-pregnancy BMI (kg/m^2).[1]

Characteristic	n	Severely Inadequate (<70%)		Moderately inadequate (70 to <90%)		Adequate (90 to <125%)	Excessive (≥125%)	
		RR (95% CI)	*P* for interaction[2]	RR (95% CI)	*P* for interaction[2]	RR (95% CI)	RR[2] (95% CI)	*P* for interaction[2]
**Preterm birth**	111,464							
Underweight (<18.5)		1.06 (0.94, 1.20)	0.906	0.98 (0.86, 1.12)	0.424	1.0	1.23 (1.00, 1.51)	0.642
Normal weight (18.5 – 24.9)		1.06 (1.00, 1.12)	Ref.	1.04 (0.98, 1.11)	Ref.	1.0	1.17 (1.08, 1.27)	Ref.
Overweight/Obese (≥ 25)		1.15 (1.00, 1.33)	0.271	0.99 (0.83, 1.19)	0.636	1.0	1.22 (1.07, 1.38)	0.581
**Low birthweight**	100,535							
Underweight (<18.5)		1.81 (1.62, 2.02)	0.072	1.25 (1.11, 1.42)	0.911	1.0	1.10 (0.88, 1.38)	0.044
Normal weight (18.5 – 24.9)		1.63 (1.53, 1.73)	Ref.	1.24 (1.16, 1.33)	Ref.	1.0	0.86 (0.78, 0.95)	Ref.
Overweight/Obese (≥ 25)		1.54 (1.29, 1.84)	0.58	1.33 (1.07, 1.66)	0.58	1.0	0.99 (0.84, 1.18)	0.142
**Small-for-gestational age**	99,800							
Underweight (<18.5)		1.62 (1.51, 1.75)	0.003	1.27 (1.17, 1.38)	0.224	1.0	0.83 (0.71, 0.98)	0.448
Normal weight (18.5 – 24.9)		1.44 (1.39, 1.50)	Ref.	1.20 (1.15, 1.26)	Ref.	1.0	0.78 (0.73, 0.83)	Ref.
Overweight/Obese (≥ 25)		1.32 (1.18, 1.48)	0.159	1.20 (1.04, 1.38)	0.959	1.0	0.84 (0.75, 0.94)	0.243
**Large-for-gestational age**	98,102							
Underweight (<18.5)		0.49 (0.38, 0.63)	0.278	0.74 (0.58, 0.96)	0.588	1.0	1.74 (1.32, 2.30)	0.988
Normal weight (18.5 – 24.9)		0.57 (0.51, 0.63)	Ref.	0.80 (0.72, 0.89)	Ref.	1.0	1.75 (1.59, 1.93)	Ref.
Overweight/Obese (≥ 25)		0.75 (0.62, 0.90)	0.008	0.67 (0.54, 0.84)	0.154	1.0	1.62 (1.40, 1.86)	0.303
**Stillbirth**	74,549							
Underweight (<18.5)		2.30 (1.25, 4.23)	0.033	1.57 (0.81, 3.07)	0.265	1.0	1.33 (0.49, 3.58)	0.551
Normal weight (18.5 – 24.9)		1.15 (0.93, 1.43)	Ref.	1.06 (0.84, 1.34)	Ref.	1.0	0.98 (0.71, 1.33)	Ref.
Overweight/Obese (≥ 25)		0.94 (0.61, 1.44)	0.396	0.92 (0.55, 1.54)	0.624	1.0	0.76 (0.51, 1.14)	0.244
**Neonatal death**	63,957							
Underweight (<18.5)		1.41 (0.87, 2.28)	0.088	1.13 (0.66, 1.93)	0.063	1.0	0.99 (0.40, 2.48)	0.587
Normal weight (18.5 – 24.9)		0.94 (0.73, 1.22)	Ref.	0.69 (0.52, 0.93)	Ref.	1.0	0.77 (0.51, 1.15)	Ref.
Overweight/Obese (≥ 25)		1.19 (0.70, 2.04)	0.433	1.06 (0.54, 2.06)	0.254	1.0	1.06 (0.65, 1.71)	0.302
**Short-for-gestational age**	90,251							
Underweight (<18.5)		1.83 (1.58, 2.12)	0.012	1.33 (1.14, 1.56)	0.252	1.0	1.24 (0.96, 1.60)	0.005
Normal weight (18.5 – 24.9)		1.51 (1.40, 1.62)	Ref.	1.21 (1.12, 1.30)	Ref.	1.0	0.83 (0.74, 0.93)	Ref.
Overweight/Obese (≥ 25)		1.42 (1.19, 1.70)	0.568	1.29 (1.05, 1.59)	0.56	1.0	0.89 (0.75, 1.05)	0.518
**Microcephaly**	84,054							
Underweight (<18.5)		2.09 (1.76, 2.49)	0.005	1.37 (1.13, 1.66)	0.514	1.0	1.01 (0.72, 1.43)	0.423
Normal weight (18.5 – 24.9)		1.63 (1.49, 1.79)	Ref.	1.28 (1.16, 1.42)	Ref.	1.0	0.88 (0.76, 1.00)	Ref.
Overweight/Obese (≥ 25)		1.44 (1.14, 1.83)	0.362	1.08 (0.79, 1.47)	0.294	1.0	0.82 (0.65, 1.03)	0.607
**Macrosomia**	92,932							
Underweight (<18.5)		0.45 (0.22, 0.93)	0.811	0.88 (0.45, 1.70)	0.263	1.0	2.95 (1.69, 5.14)	0.162
Normal weight (18.5 – 24.9)		0.49 (0.37, 0.65)	Ref.	0.59 (0.45, 0.78)	Ref.	1.0	1.95 (1.62, 2.35)	Ref.
Overweight/Obese (≥ 25)		0.59 (0.41, 0.86)	0.36	0.63 (0.41, 0.98)	0.766	1.0	1.49 (1.15, 1.92)	0.041

[1]Multivariable modified Poisson models with robust variance adjusted for maternal age, study intervention arm (if applicable), and study fixed effects using a 1-stage meta-analysis approach. For stillbirth, neonatal death, and macrosomia, measures of association with GWG adequacy were estimated using odds ratio given very low prevalence of these outcomes and convergence issues with modified Poisson regression. Odds ratios approximate relative risk in the context of rare outcomes. [2] P-value for interaction between gestational weight gain adequacy ratio and maternal pre-pregnancy BMI.

Supplementary Table 10: Associations between percent adequacy of total gestational weight gain and neonatal outcomes among women with last measure in the third trimester, modified by maternal pre-pregnancy BMI (kg/m^2).[1]

Characteristic	n	Severely inadequate (<70%)		Moderately inadequate (70 to <90%)		Adequate (90 to <125%)	Excessive (≥125%)	
		RR[2] (95% CI)	*P* for interaction[2]	RR[2] (95% CI)	*P* for interaction[2]	RR (95% CI)	RR[2] (95% CI)	*P* for interaction[2]
**Preterm birth**	103,702							
Underweight (<18.5)		1.05 (0.92, 1.19)	0.565	0.99 (0.86, 1.14)	0.516	1.0	1.23 (0.98, 1.53)	0.998
Normal weight (18.5 – 24.9)		1.09 (1.02, 1.16)	Ref.	1.04 (0.97, 1.12)	Ref.	1.0	1.23 (1.13, 1.34)	Ref.
Overweight/Obese (≥ 25)		1.16 (0.99, 1.35)	0.498	1.03 (0.84, 1.25)	0.895	1.0	1.15 (1.00, 1.32)	0.409
**Low birthweight**	94,785							
Underweight (<18.5)		1.84 (1.64, 2.07)	0.077	1.26 (1.11, 1.44)	0.900	1.0	1.12 (0.89, 1.41)	0.049
Normal weight (18.5 – 24.9)		1.65 (1.55, 1.76)	Ref.	1.25 (1.17, 1.35)	Ref.	1.0	0.87 (0.79, 0.96)	Ref.
Overweight/Obese (≥ 25)		1.56 (1.29, 1.88)	0.570	1.35 (1.07, 1.69)	0.568	1.0	0.97 (0.82, 1.16)	0.267
**Small-for-gestational age**	94,167							
Underweight (<18.5)		1.62 (1.51, 1.75)	0.005	1.26 (1.16, 1.37)	0.311	1.0	0.86 (0.72, 1.01)	0.243
Normal weight (18.5 – 24.9)		1.45 (1.39, 1.51)	Ref.	1.21 (1.15, 1.26)	Ref.	1.0	0.77 (0.72, 0.82)	Ref.
Overweight/Obese (≥ 25)		1.36 (1.21, 1.52)	0.306	1.19 (1.03, 1.37)	0.862	1.0	0.86 (0.77, 0.96)	0.1
**Large-for-gestational age**	92,498							
Underweight (<18.5)		0.44 (0.33, 0.58)	0.08	0.73 (0.56, 0.96)	0.719	1.0	1.82 (1.36, 2.44)	0.947
Normal weight (18.5 – 24.9)		0.57 (0.51, 0.64)	Ref.	0.77 (0.69, 0.86)	Ref.	1.0	1.80 (1.63, 2.00)	Ref.
Overweight/Obese (≥ 25)		0.74 (0.61, 0.90)	0.018	0.70 (0.55, 0.88)	0.418	1.0	1.59 (1.37, 1.84)	0.1
**Stillbirth**	66,078							
Underweight (<18.5)		1.77 (0.87, 3.57)	0.286	1.59 (0.75, 3.38)	0.323	1.0	1.39 (0.46, 4.20)	0.432
Normal weight (18.5 – 24.9)		1.18 (0.92, 1.52)	Ref.	1.07 (0.81, 1.40)	Ref.	1.0	0.88 (0.59, 1.31)	Ref.
Overweight/Obese (≥ 25)		0.78 (0.48, 1.28)	0.138	0.96 (0.55, 1.65)	0.713	1.0	0.58 (0.35, 0.95)	0.088
**Neonatal death**	59,237							
Underweight (<18.5)		1.34 (0.81, 2.22)	0.178	1.19 (0.68, 2.09)	0.075	1.0	0.98 (0.36, 2.63)	0.609
Normal weight (18.5 – 24.9)		0.96 (0.73, 1.27)	Ref.	0.73 (0.53, 1.00)	Ref.	1.0	0.75 (0.48, 1.20)	Ref.
Overweight/Obese (≥ 25)		1.14 (0.65, 2.00)	0.575	1.13 (0.57, 2.21)	0.249	1.0	1.00 (0.60, 1.66)	0.407
**Short-for-gestational age**	86,158							
Underweight (<18.5)		1.83 (1.58, 2.12)	0.006	1.34 (1.14, 1.58)	0.184	1.0	1.29 (0.99, 1.67)	0.002
Normal weight (18.5 – 24.9)		1.47 (1.37, 1.59)	Ref.	1.20 (1.11, 1.29)	Ref.	1.0	0.81 (0.72, 0.92)	Ref.
Overweight/Obese (≥ 25)		1.46 (1.22, 1.75)	0.911	1.28 (1.04, 1.59)	0.544	1.0	0.88 (0.74, 1.04)	0.472
**Microcephaly**	80,054							
Underweight (<18.5)		2.10 (1.76, 2.50)	0.003	1.39 (1.14, 1.69)	0.345	1.0	1.09 (0.77, 1.56)	0.227
Normal weight (18.5 – 24.9)		1.60 (1.46, 1.76)	Ref.	1.26 (1.14, 1.39)	Ref.	1.0	0.87 (0.76, 1.00)	Ref.
Overweight/Obese (≥ 25)		1.48 (1.16, 1.88)	0.551	1.08 (0.79, 1.47)	0.350	1.0	0.84 (0.66, 1.06)	0.796
**Macrosomia**	87,525							
Underweight (<18.5)		0.38 (0.17, 0.84)	0.639	0.74 (0.37, 1.50)	0.554	1.0	3.24 (1.84, 5.70)	0.092
Normal weight (18.5 – 24.9)		0.46 (0.34, 0.62)	Ref.	0.59 (0.44, 0.80)	Ref.	1.0	1.95 (1.61, 2.36)	Ref.
Overweight/Obese (≥ 25)		0.62 (0.42, 0.92)	0.156	0.63 (0.40, 1.00)	0.794	1.0	1.56 (1.20, 2.02)	0.093

[1] Multivariable modified Poisson models with robust variance adjusted for maternal age, study intervention arm (if applicable), and study fixed effects using a one-stage meta-analysis approach. For stillbirth, neonatal death, and macrosomia, measures of association with GWG adequacy were estimated using odds ratio given very low prevalence of these outcomes and convergence issues with modified Poisson regression. Odds ratios approximate relative risk in the context of rare outcomes. [2] P-value for interaction between gestational weight gain adequacy ratio and maternal pre-pregnancy BMI.

Supplementary Table 11: Associations between percent adequacy of total gestational weight gain and neonatal outcomes, modified by maternal age (years).[1]

Characteristic	n	Severely inadequate (<70%)		Moderately inadequate (70 to <90%)		Adequate (90 to <125%)	Excessive (≥125%)	
		RR^2 (95% CI)	*P* for interaction[2]	RR^2 (95% CI)	*P* for interaction[2]	RR (95% CI)	RR^2 (95% CI)	*P* for interaction[2]
**Preterm birth**	111,464							
<20		1.14 (1.03, 1.27)	0.032	1.14 (1.02, 1.29)	0.228	1.0	1.16 (0.99, 1.36)	0.963
20 – 29		1.00 (0.94, 1.06)	Ref.	1.05 (0.98, 1.12)	Ref.	1.0	1.16 (1.07, 1.25)	Ref.
≥30		0.98 (0.89, 1.09)	0.776	0.97 (0.87, 1.09)	0.248	1.0	1.21 (1.07, 1.36)	0.5
**Low birthweight**	100,535							
<20		1.64 (1.50, 1.80)	0.964	1.34 (1.21, 1.48)	0.163	1.0	0.86 (0.72, 1.02)	0.263
20 – 29		1.64 (1.54, 1.75)	Ref.	1.23 (1.14, 1.32)	Ref.	1.0	0.96 (0.87, 1.06)	Ref.
≥30		1.63 (1.46, 1.83)	0.949	1.24 (1.09, 1.41)	0.891	1.0	0.99 (0.85, 1.16)	0.714
**Small-for-gestational age**	99,800							
<20		1.31 (1.23, 1.38)	<0.001	1.17 (1.10, 1.25)	0.323	1.0	0.74 (0.66, 0.84)	0.106
20 – 29		1.47 (1.42, 1.53)	Ref.	1.22 (1.17, 1.27)	Ref.	1.0	0.84 (0.78, 0.89)	Ref.
≥30		1.55 (1.43, 1.67)	0.292	1.24 (1.14, 1.36)	0.719	1.0	0.81 (0.73, 0.91)	0.669
**Large-for-gestational age**	98,102							
<20		0.78 (0.62, 0.98)	0.042	0.98 (0.77, 1.26)	0.093	1.0	2.04 (1.59, 2.60)	0.004
20 – 29		0.60 (0.54, 0.66)	Ref.	0.78 (0.70, 0.87)	Ref.	1.0	1.36 (1.23, 1.51)	Ref.
≥30		0.61 (0.52, 0.71)	0.88	0.75 (0.64, 0.88)	0.68	1.0	1.41 (1.23, 1.62)	0.595
**Stillbirth**	74,549							
<20		1.11 (0.72, 1.71)	0.998	1.02 (0.63, 1.66)	0.808	1.0	0.75 (0.37, 1.52)	0.503
20 – 29		1.11 (0.89, 1.39)	Ref.	0.96 (0.75, 1.23)	Ref.	1.0	0.97 (0.69, 1.35)	Ref.
≥30		1.16 (0.84, 1.61)	0.818	1.18 (0.82, 1.68)	0.336	1.0	0.70 (0.46, 1.07)	0.123
**Neonatal death**	63,957							
<20		1.01 (0.74, 1.39)	0.259	0.63 (0.43, 0.91)	0.409	1.0	0.90 (0.51, 1.59)	0.504
20 – 29		0.83 (0.67, 1.02)	Ref.	0.75 (0.59, 0.95)	Ref.	1.0	1.11 (0.82, 1.51)	Ref.
≥30		0.78 (0.52, 1.15)	0.784	0.69 (0.44, 1.08)	0.76	1.0	1.03 (0.61, 1.75)	0.798
**Short-for-gestational age**	90,251							
<20		1.44 (1.29, 1.60)	0.22	1.24 (1.10, 1.40)	0.742	1.0	0.76 (0.61, 0.93)	0.021
20 – 29		1.56 (1.46, 1.67)	Ref.	1.21 (1.12, 1.30)	Ref.	1.0	1.00 (0.90, 1.11)	Ref.
≥30		1.49 (1.32, 1.68)	0.545	0.97 (0.84, 1.13)	0.009	1.0	0.85 (0.72, 1.01)	0.099
**Microcephaly**	84,054							
<20		1.39 (1.23, 1.58)	0.105	1.27 (1.10, 1.46)	0.518	1.0	0.83 (0.65, 1.05)	0.437
20 – 29		1.58 (1.45, 1.71)	Ref.	1.20 (1.09, 1.31)	Ref.	1.0	0.93 (0.81, 1.06)	Ref.
≥30		1.72 (1.47, 2.01)	0.338	1.16 (0.97, 1.40)	0.772	1.0	0.90 (0.71, 1.12)	0.791
**Macrosomia**	92,932							
<20		1.15 (0.63, 2.09)	0.009	0.88 (0.44, 1.77)	0.241	1.0	2.99 (1.66, 5.40)	0.027
20 – 29		0.49 (0.39, 0.61)	Ref.	0.57 (0.44, 0.72)	Ref.	1.0	1.47 (1.21, 1.80)	Ref.
≥30		0.59 (0.43, 0.83)	0.309	0.69 (0.49, 0.98)	0.319	1.0	1.57 (1.22, 2.02)	0.61

[1] Multivariable modified Poisson models with robust variance adjusted for maternal pre-pregnancy BMI, study intervention arm (if applicable), and study fixed effects using a one-stage meta-analysis approach. For stillbirth, neonatal death, and macrosomia, measures of association with GWG adequacy were estimated using odds ratio given very low prevalence of these outcomes and convergence issues with modified Poisson regression. Odds ratios approximate relative risk in the context of rare outcomes. [2] P-value for interaction between gestational weight gain adequacy ratio and maternal age.

Supplementary Table 12: Associations between percent adequacy of gestational weight gain and neonatal outcomes among women with last measure in the third trimester, modified by maternal age (years).[1]

Characteristic	n	Severely inadequate (<70%)		Moderately inadequate (70 to <90%)		Adequate (70 to <125%)	Excessive (≥125%)	
		RR (95% CI)	*P* for interaction[2]	RR (95% CI)	*P* for interaction[2]	RR (95% CI)	RR (95% CI)	*P* for interaction[2]
**Preterm birth**	103,702							
<20		1.18 (1.04, 1.33)	0.038	1.11 (0.97, 1.27)	0.595	1.0	1.24 (1.03, 1.49)	0.419
20 – 29		1.02 (0.95, 1.09)	Ref.	1.06 (0.98, 1.14)	Ref.	1.0	1.14 (1.04, 1.25)	Ref.
≥30		1.01 (0.90, 1.13)	0.912	0.98 (0.87, 1.12)	0.317	1.0	1.21 (1.06, 1.38)	0.397
**Low birthweight**	94,785							
<20		1.66 (1.51, 1.82)	0.717	1.31 (1.18, 1.46)	0.484	1.0	0.84 (0.70, 1.01)	0.156
20 – 29		1.69 (1.58, 1.81)	Ref.	1.25 (1.16, 1.35)	Ref.	1.0	0.98 (0.88, 1.09)	Ref.
≥30		1.63 (1.45, 1.83)	0.604	1.25 (1.09, 1.42)	0.951	1.0	0.94 (0.80, 1.11)	0.697
**Small-for-gestational age**	94,167							
<20		1.32 (1.24, 1.40)	0.001	1.18 (1.11, 1.26)	0.451	1.0	0.72 (0.63, 0.82)	0.046
20 – 29		1.48 (1.42, 1.54)	Ref.	1.22 (1.16, 1.27)	Ref.	1.0	0.84 (0.78, 0.90)	Ref.
≥30		1.55 (1.43, 1.68)	0.301	1.24 (1.14, 1.36)	0.673	1.0	0.81 (0.72, 0.91)	0.556
**Large-for-gestational age**	92,498							
<20		0.76 (0.58, 1.00)	0.073	0.86 (0.63, 1.17)	0.477	1.0	2.40 (1.81, 3.19)	0.001
20 – 29		0.58 (0.52, 0.65)	Ref.	0.76 (0.68, 0.86)	Ref.	1.0	1.38 (1.23, 1.54)	Ref.
≥30		0.61 (0.52, 0.72)	0.525	0.77 (0.65, 0.92)	0.878	1.0	1.47 (1.27, 1.70)	0.37
**Stillbirth**	66,078							
<20		1.29 (0.76, 2.19)	0.585	1.08 (0.60, 1.97)	0.866	1.0	0.99 (0.42, 2.30)	0.868
20 – 29		1.09 (0.84, 1.41)	Ref.	1.02 (0.77, 1.36)	Ref.	1.0	0.92 (0.61, 1.38)	Ref.
≥30		1.04 (0.71, 1.52)	0.813	1.19 (0.79, 1.79)	0.540	1.0	0.59 (0.35, 0.99)	0.076
**Neonatal death**	59,237							
<20		1.11 (0.79, 1.55)	0.153	0.61 (0.40, 0.92)	0.395	1.0	0.98 (0.54, 1.79)	0.941
20 – 29		0.84 (0.67, 1.05)	Ref.	0.74 (0.57, 0.95)	Ref.	1.0	1.01 (0.71, 1.41)	Ref.
≥30		0.91 (0.59, 1.41)	0.748	0.81 (0.50, 1.33)	0.727	1.0	1.10 (0.61, 1.99)	0.790
**Short-for-gestational age**	86,158							
<20		1.40 (1.25, 1.56)	0.094	1.20 (1.06, 1.36)	0.867	1.0	0.70 (0.56, 0.87)	0.006
20 – 29		1.56 (1.45, 1.67)	Ref.	1.22 (1.13, 1.32)	Ref.	1.0	1.00 (0.90, 1.11)	Ref.
≥30		1.48 (1.30, 1.67)	0.445	0.95 (0.82, 1.10)	0.004	1.0	0.84 (0.70, 1.00)	0.07
**Microcephaly**	80,054							
<20		1.41 (1.23, 1.61)	0.181	1.27 (1.10, 1.47)	0.472	1.0	0.82 (0.64, 1.06)	0.445
20 – 29		1.56 (1.44, 1.70)	Ref.	1.19 (1.08, 1.31)	Ref.	1.0	0.93 (0.80, 1.06)	Ref.
≥30		1.71 (1.45, 2.01)	0.336	1.17 (0.97, 1.41)	0.852	1.0	0.87 (0.69, 1.11)	0.676
**Macrosomia**	87,525							
<20		1.00 (0.51, 1.94)	0.061	0.87 (0.40, 1.90)	0.321	1.0	3.33 (1.77, 6.28)	0.025
20 – 29		0.50 (0.40, 0.64)	Ref.	0.57 (0.43, 0.74)	Ref.	1.0	1.54 (1.24, 1.91)	Ref.
≥30		0.58 (0.41, 0.82)	0.503	0.73 (0.50, 1.05)	0.221	1.0	1.67 (1.28, 2.18)	0.521

[1] Multivariable modified Poisson models with robust variance adjusted for maternal pre-pregnancy BMI, study intervention arm (if applicable), and study fixed effects using a one-stage meta-analysis approach. For stillbirth, neonatal death, and macrosomia, measures of association with GWG adequacy were estimated using odds ratio given very low prevalence of these outcomes and convergence issues with modified Poisson regression. Odds ratios approximate relative risk in the context of rare outcomes. [2] P-value for interaction between gestational weight gain adequacy ratio and maternal age.

Supplemental Figure 2a: Forest plots of the association between **preterm birth** and severely inadequate gestational weight gain (<70%).

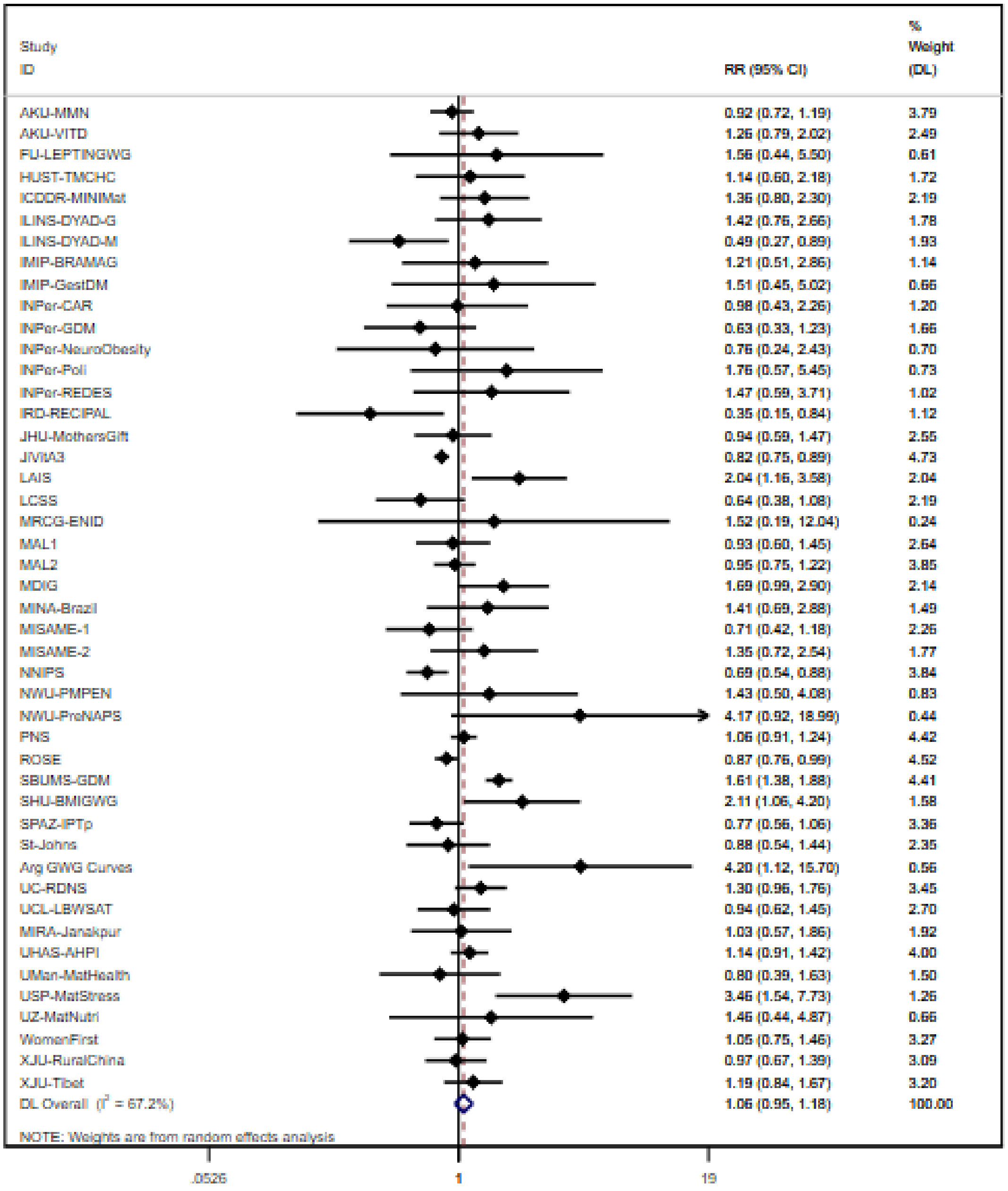

Supplemental Figure 2b: Forest plots of the association between **preterm birth** and moderately inadequate gestational weight gain (70 to <90%).

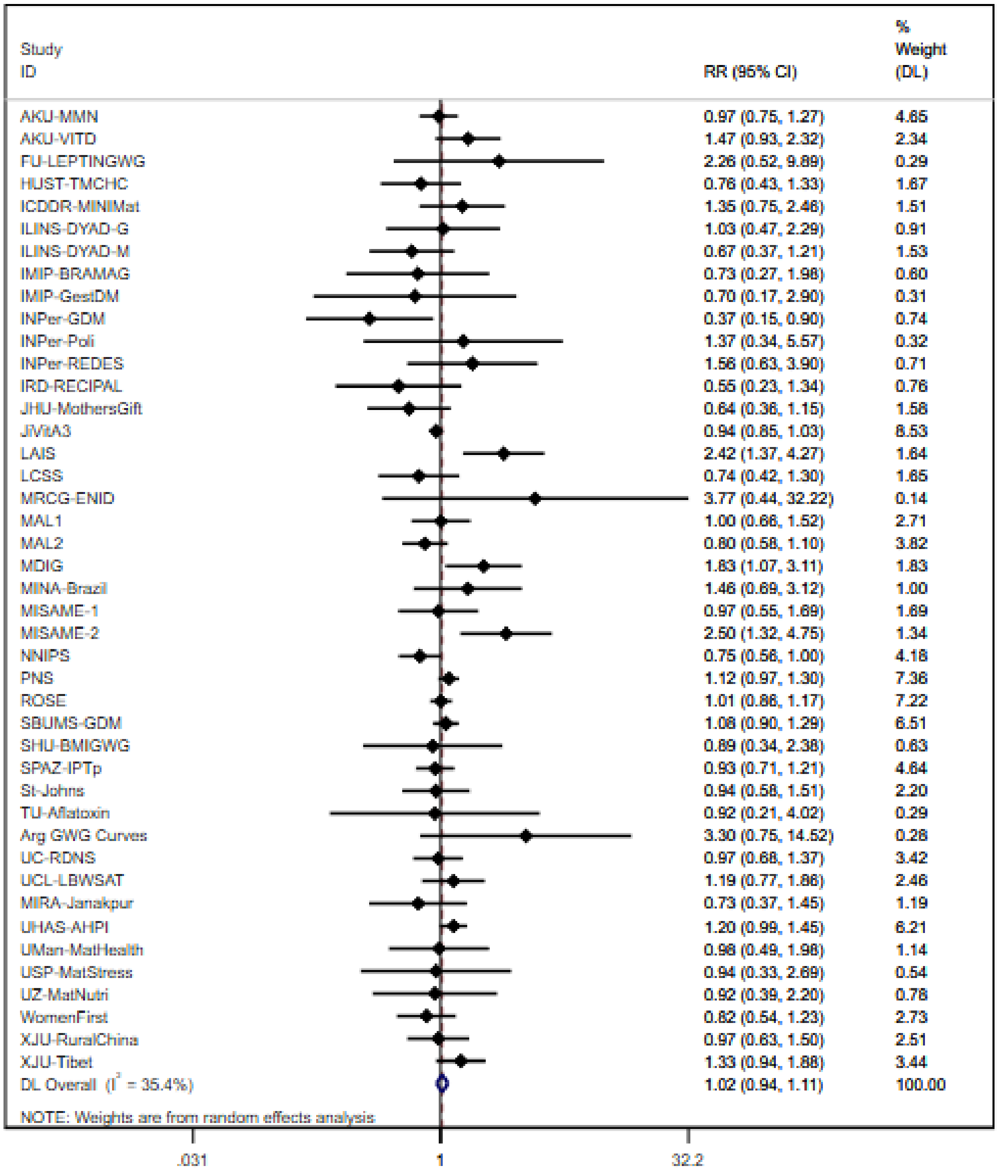

Supplemental Figure 2c: Forest plots of the association between **preterm birth** and excessive gestational weight gain (≥125%).

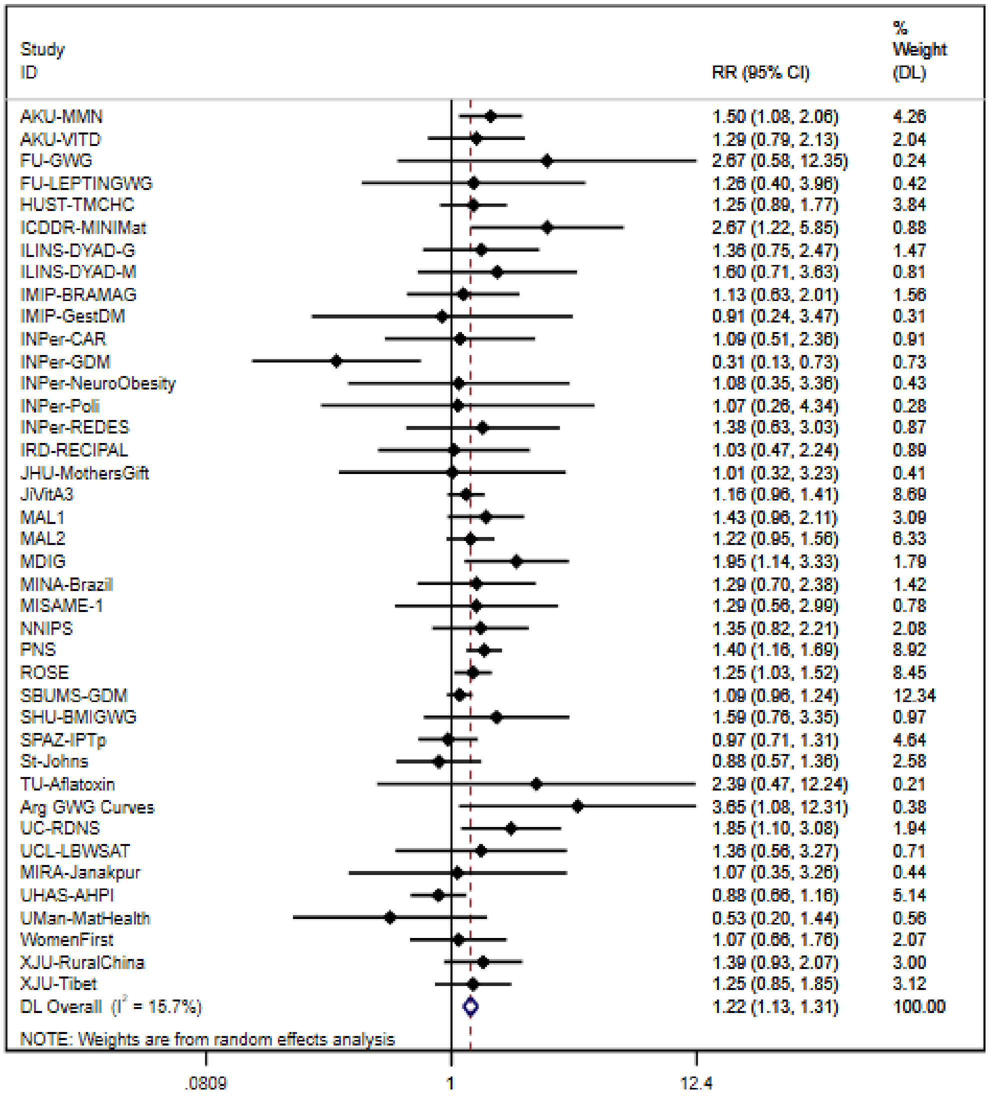

Supplemental Figure 3a: Forest plots of the association between **low birthweight** and severely inadequate gestational weight gain (<70%).

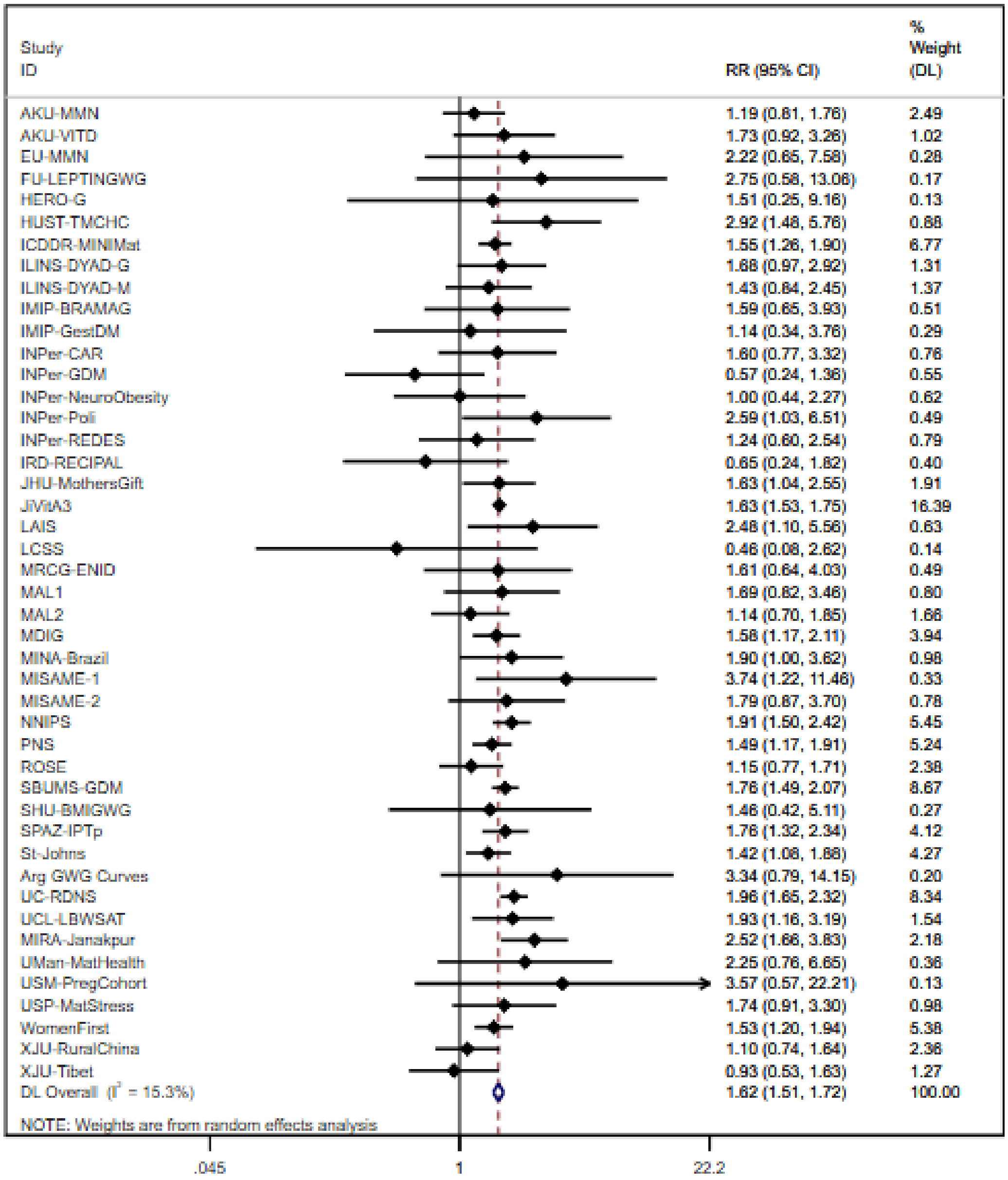

Supplemental Figure 3b: Forest plots of the association between **low birthweight** and moderately inadequate gestational weight gain (70 to <90%).

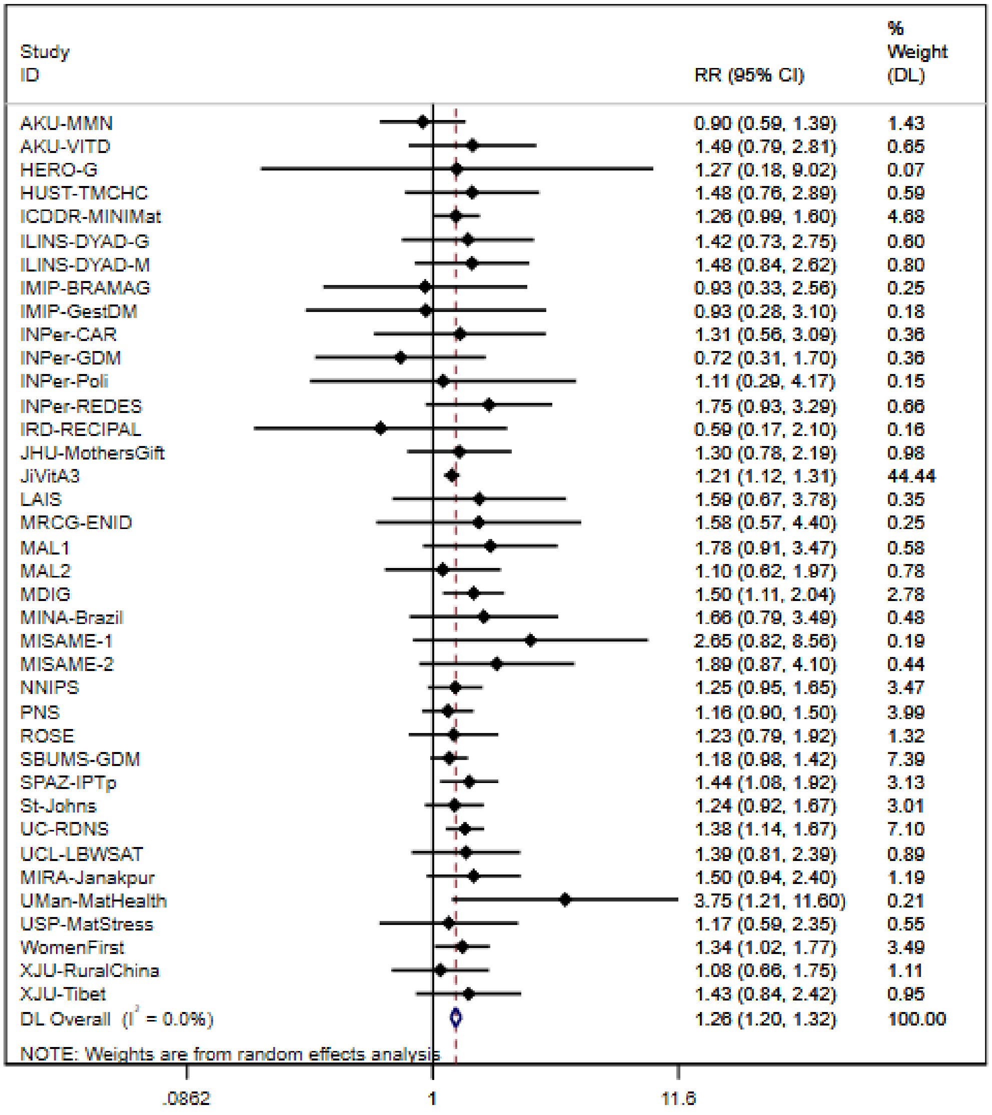

Supplemental Figure 3c: Forest plots of the association between **low birthweight** and excessive gestational weight gain (≥125%).

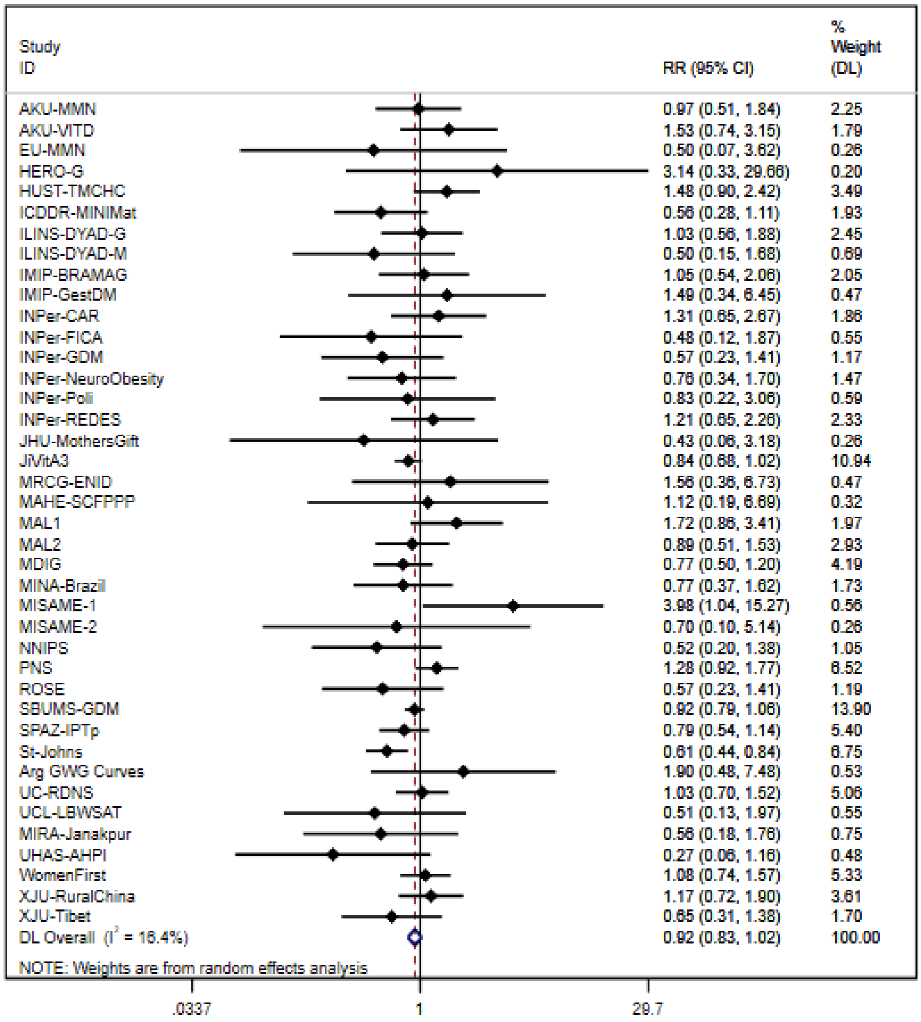

Supplemental Figure 4a: Forest plots of the association between **small-for-gestational age** and severely inadequate gestational weight gain (<70%).

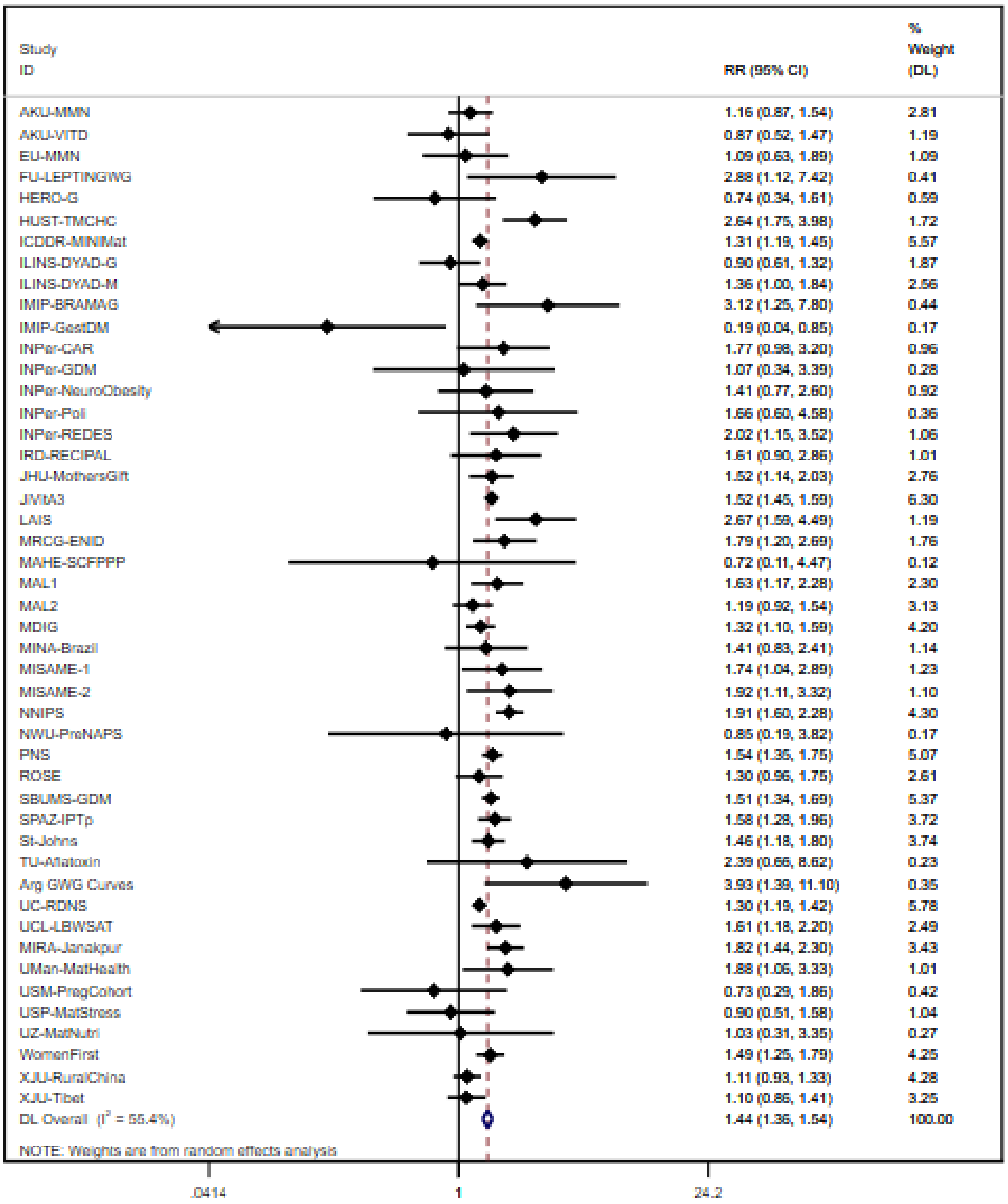

Supplemental Figure 4b: Forest plots of the association between **small-for-gestational age** and moderately inadequate gestational weight gain (70 to <90%).

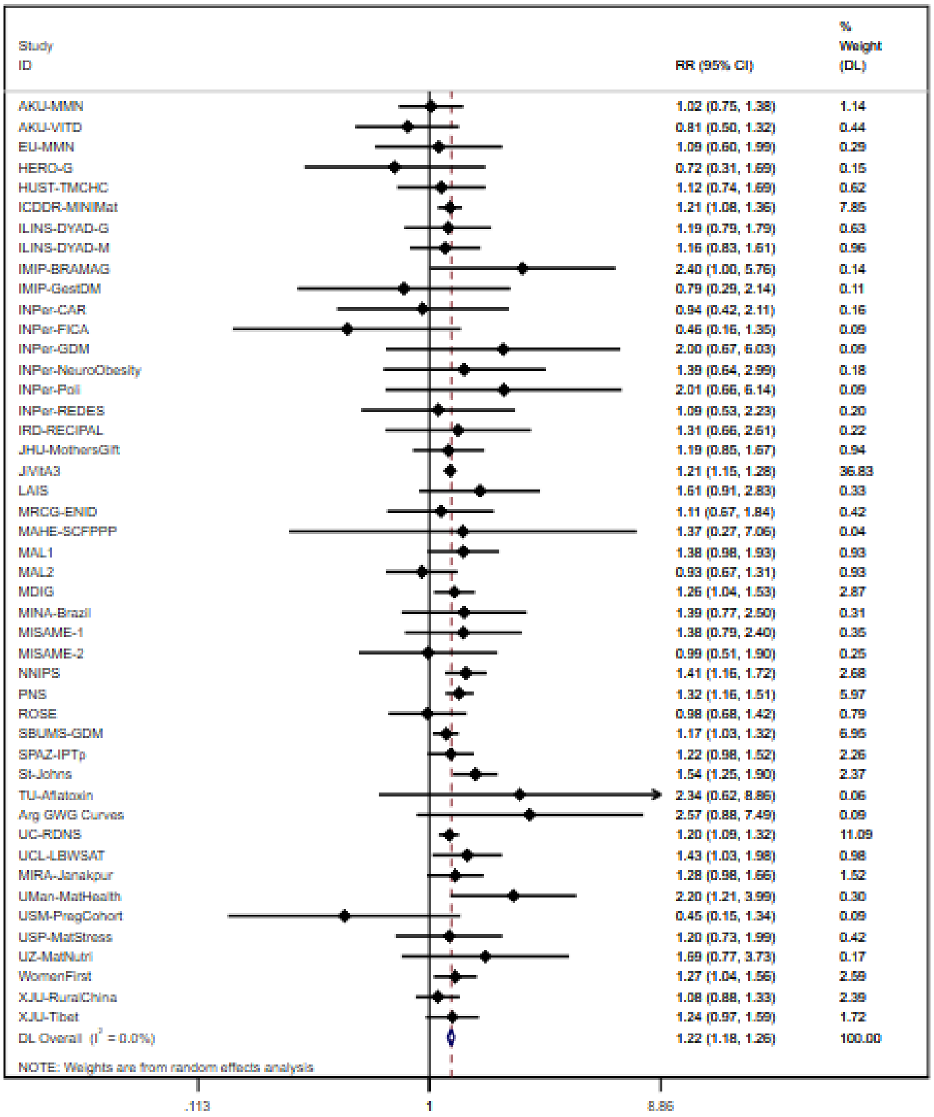

Supplemental Figure 4c: Forest plots of the association between **small-for-gestational age** and excessive gestational weight gain (≥125%).

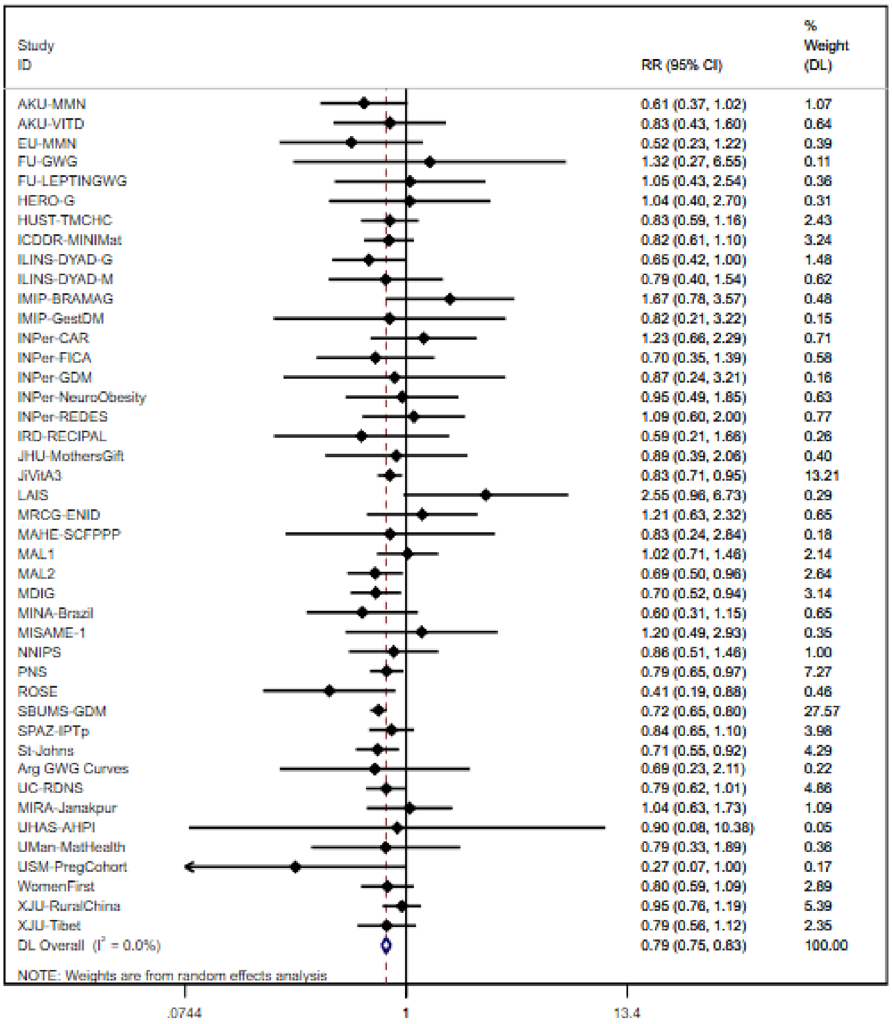

Supplemental Figure 5a: Forest plots of the association between **large-for-gestational age** and severely inadequate gestational weight gain (<70%).

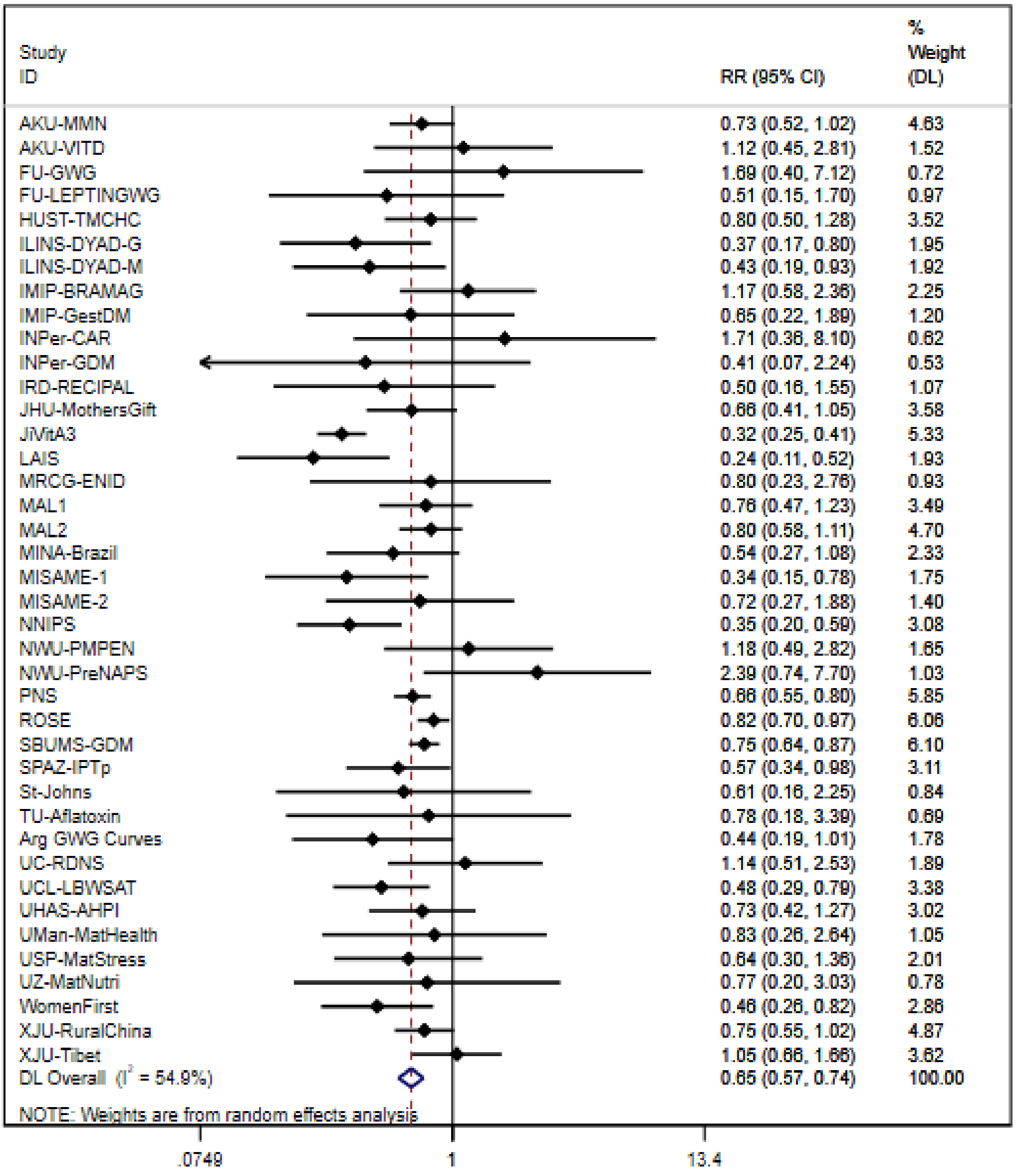

Supplemental Figure 5b: Forest plots of the association between **large-for-gestational age** and moderately inadequate gestational weight gain (70 to <90%).

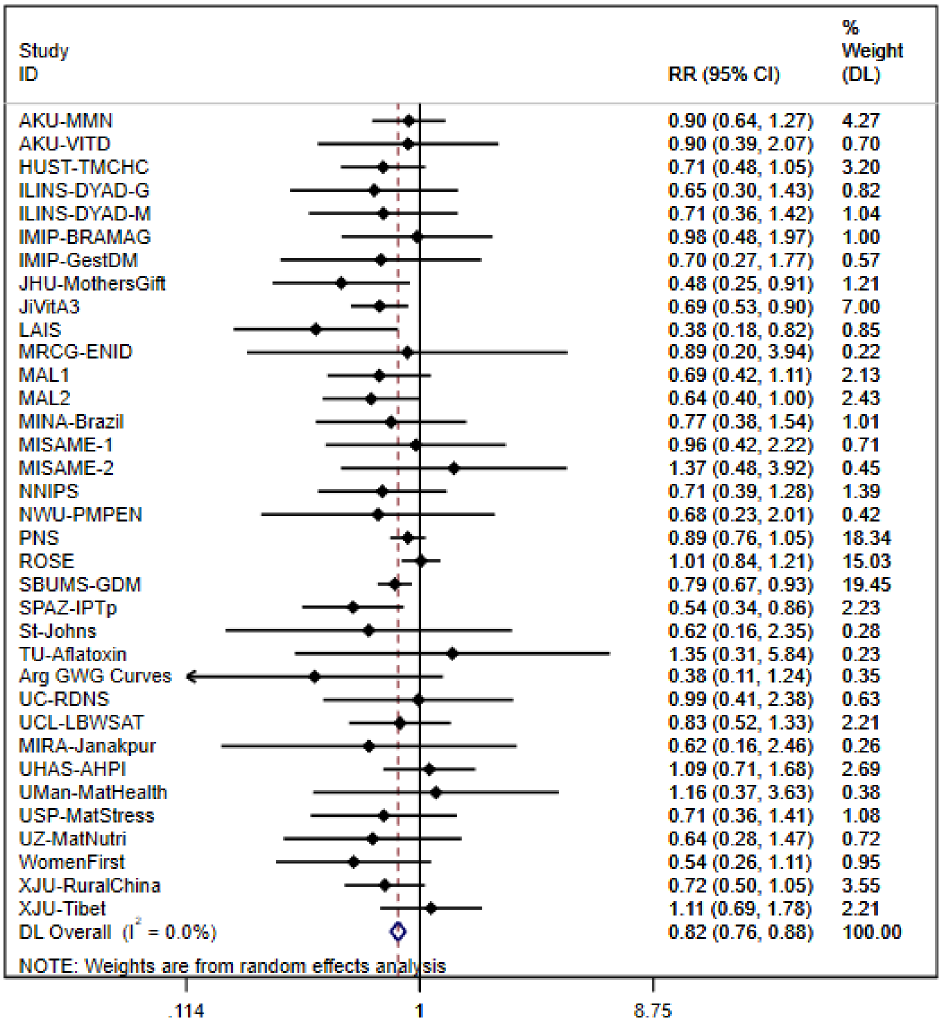

Supplemental Figure 5c: Forest plots of the association between **large-for-gestational age** and excessive gestational weight gain (≥125%).

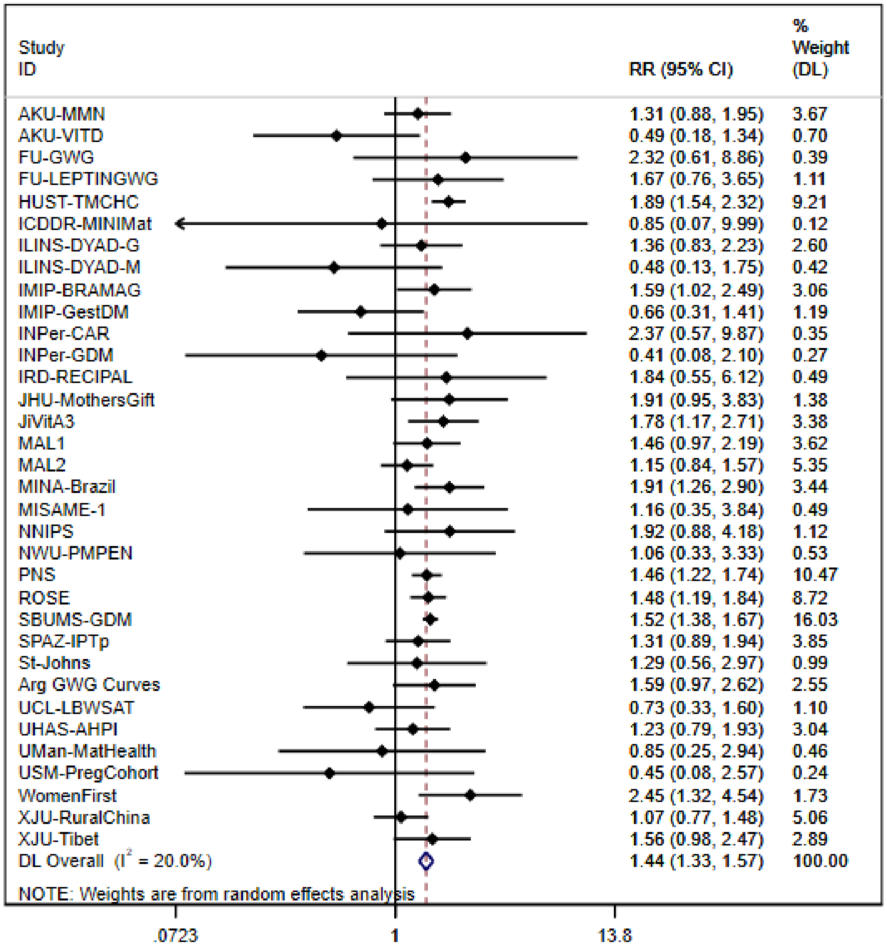

Supplemental Figure 6a: Forest plots of the association between **stillbirth** and severely inadequate gestational weight gain (<70%).

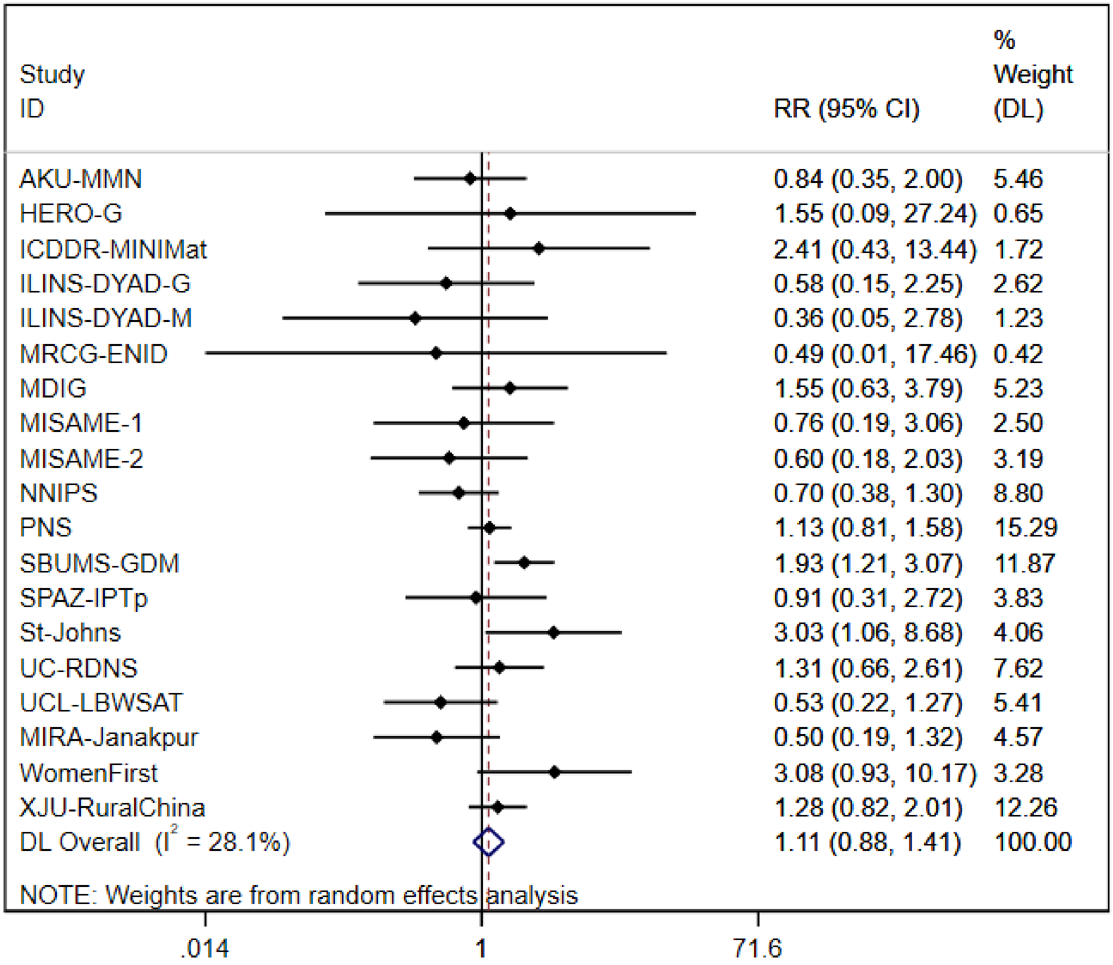

Supplemental Figure 6b: Forest plots of the association between **stillbirth** and moderately inadequate gestational weight gain (70 to <90%).

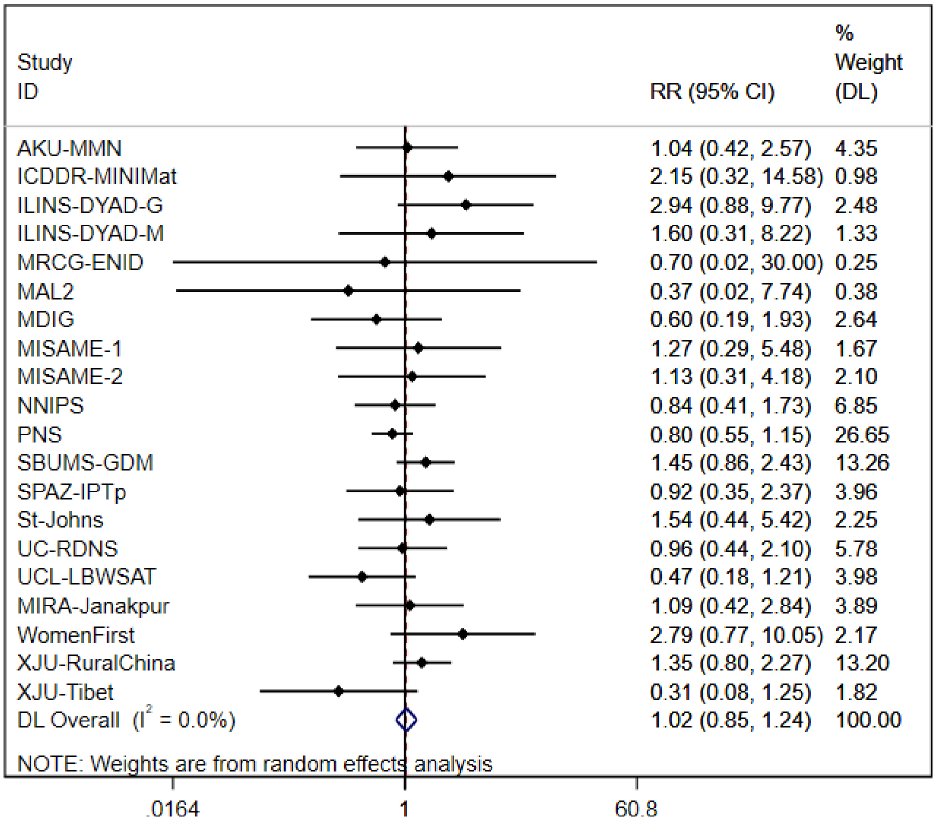

Supplemental Figure 6c: Forest plots of the association between **stillbirth** and excessive gestational weight gain (≥125%).

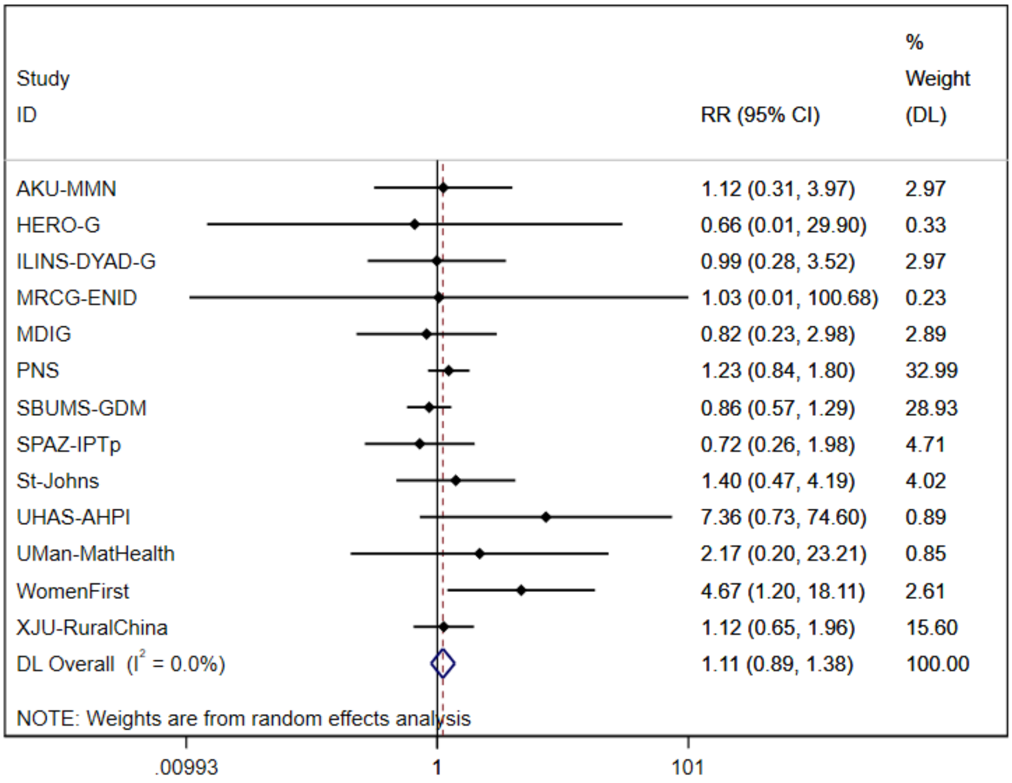

Supplemental Figure 7a: Forest plots of the association between **neonatal death** and severely inadequate gestational weight gain (<70%).

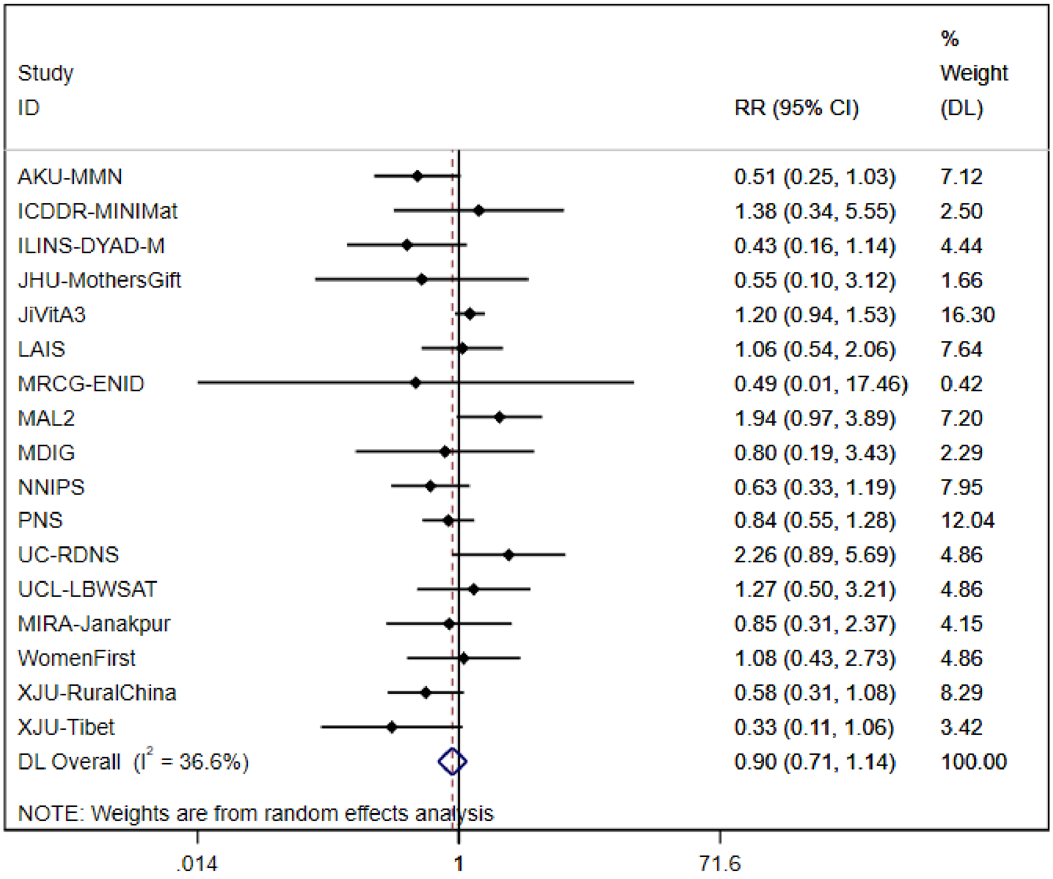

Supplemental Figure 7b: Forest plots of the association between **neonatal death** and moderately inadequate gestational weight gain (70 to <90%).

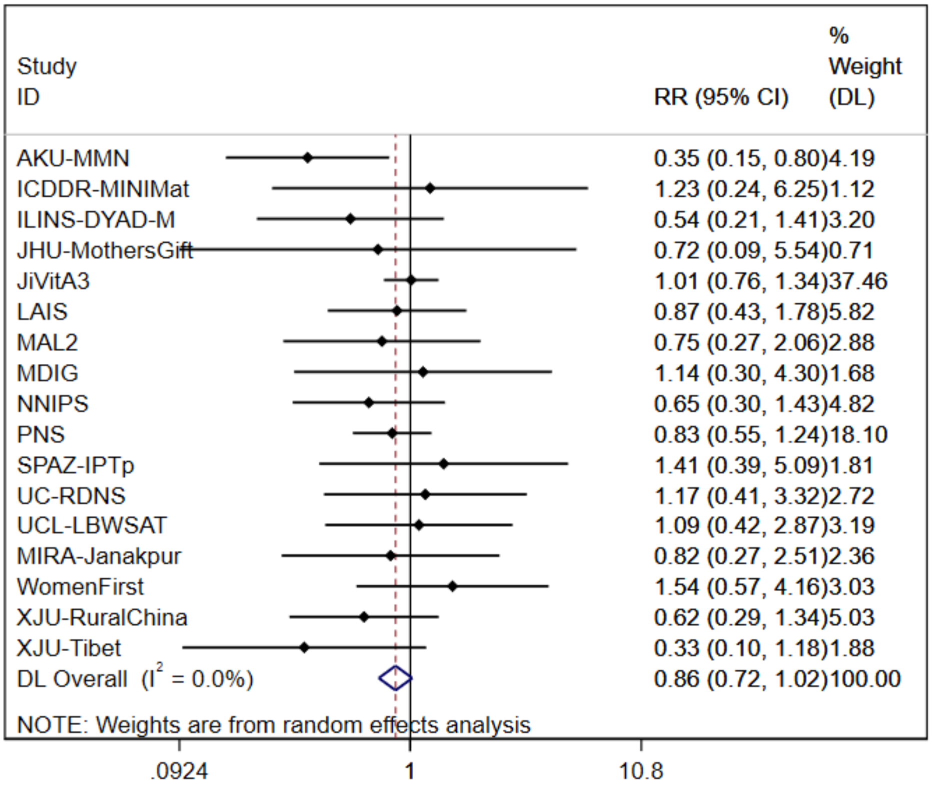

Supplemental Figure 7c: Forest plots of the association between **neonatal death** and excessive gestational weight gain (≥125%).

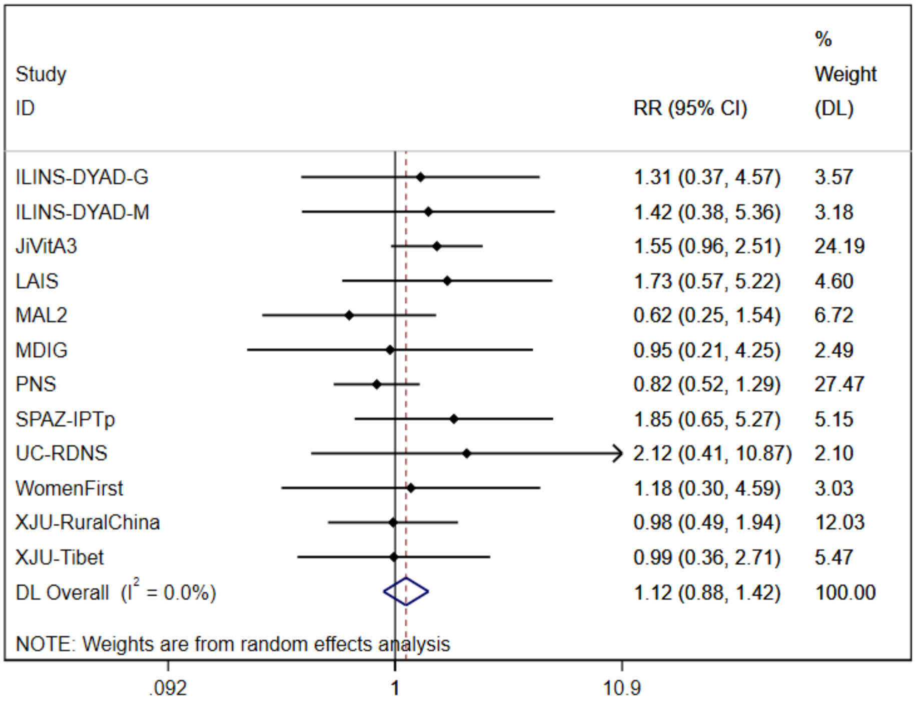

Supplemental Figure 8a: Forest plots of the association between **short-for-gestational age** and severely inadequate gestational weight gain (<70%).

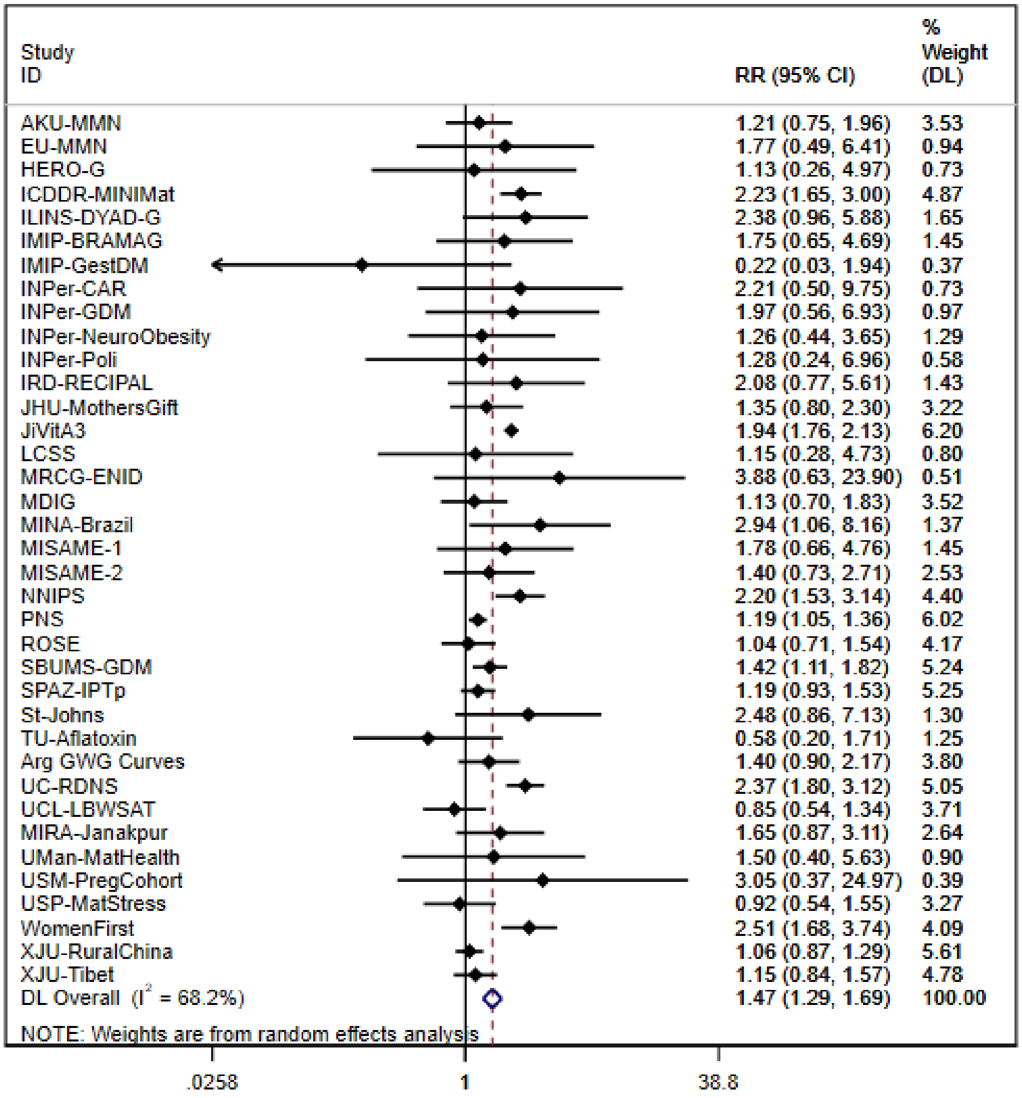

Supplemental Figure 8b: Forest plots of the association between **short-for-gestational age** and moderately inadequate gestational weight gain (70 to <90%).

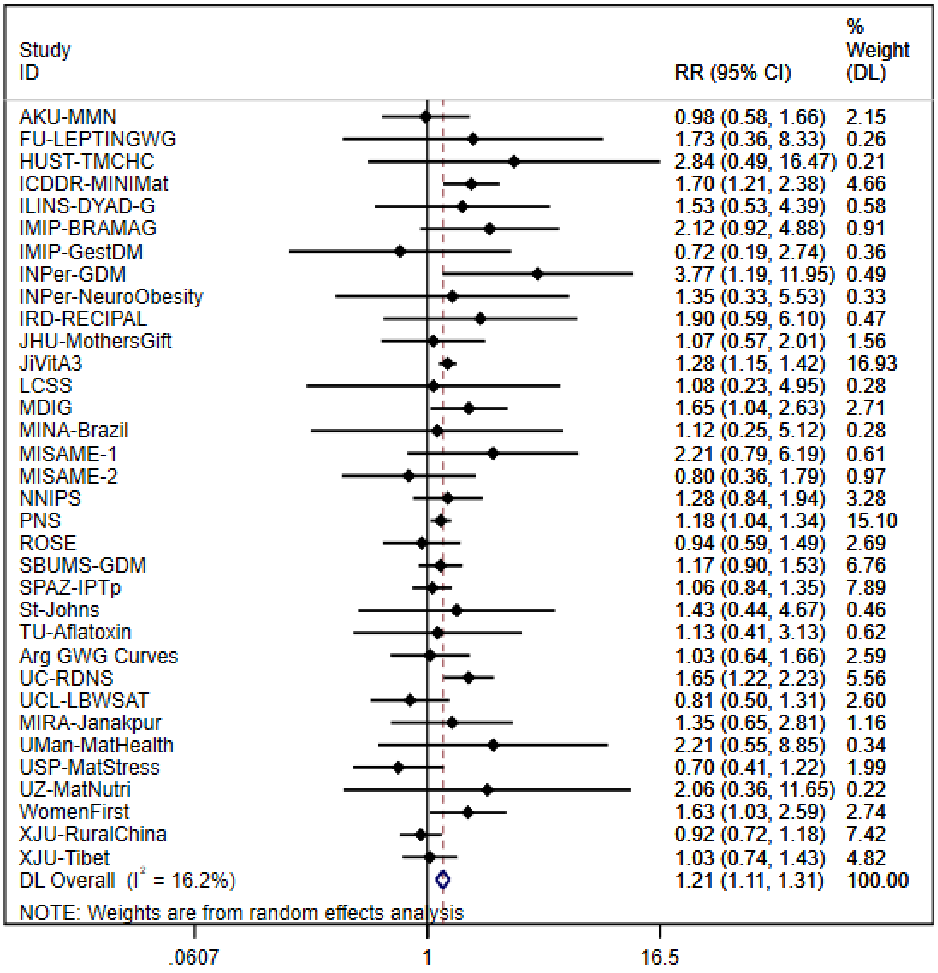

Supplemental Figure 8c: Forest plots of the association between **short-for-gestational age** and excessive gestational weight gain (≥125%).

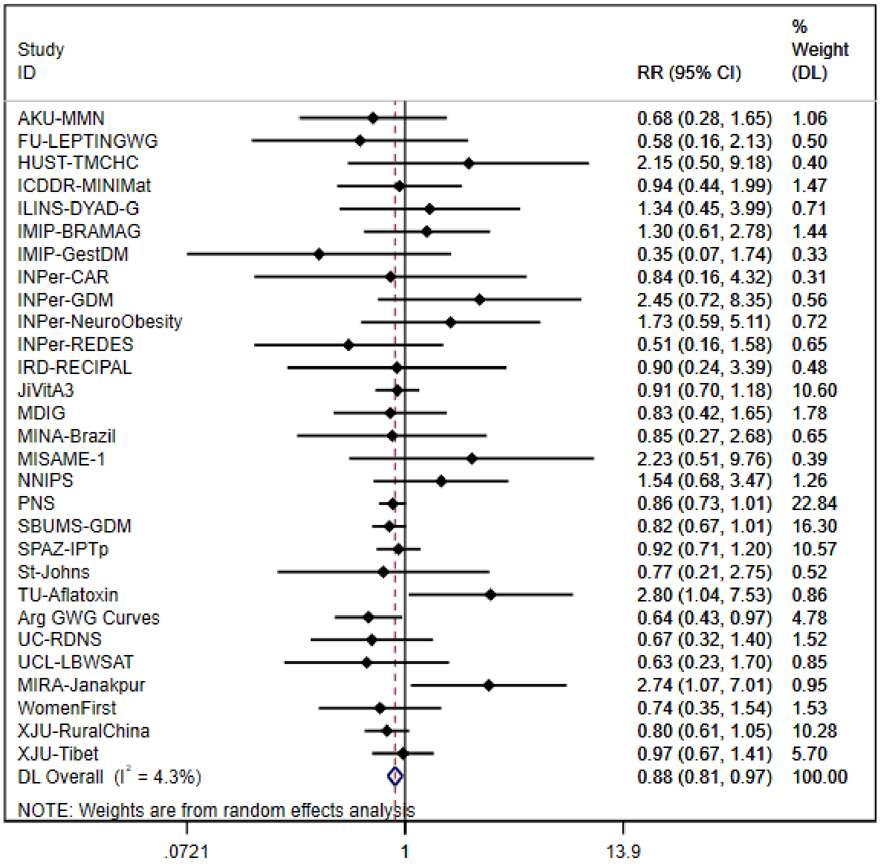

Supplemental Figure 9a: Forest plots of the association between **microcephaly** and severely inadequate gestational weight gain (<70%).

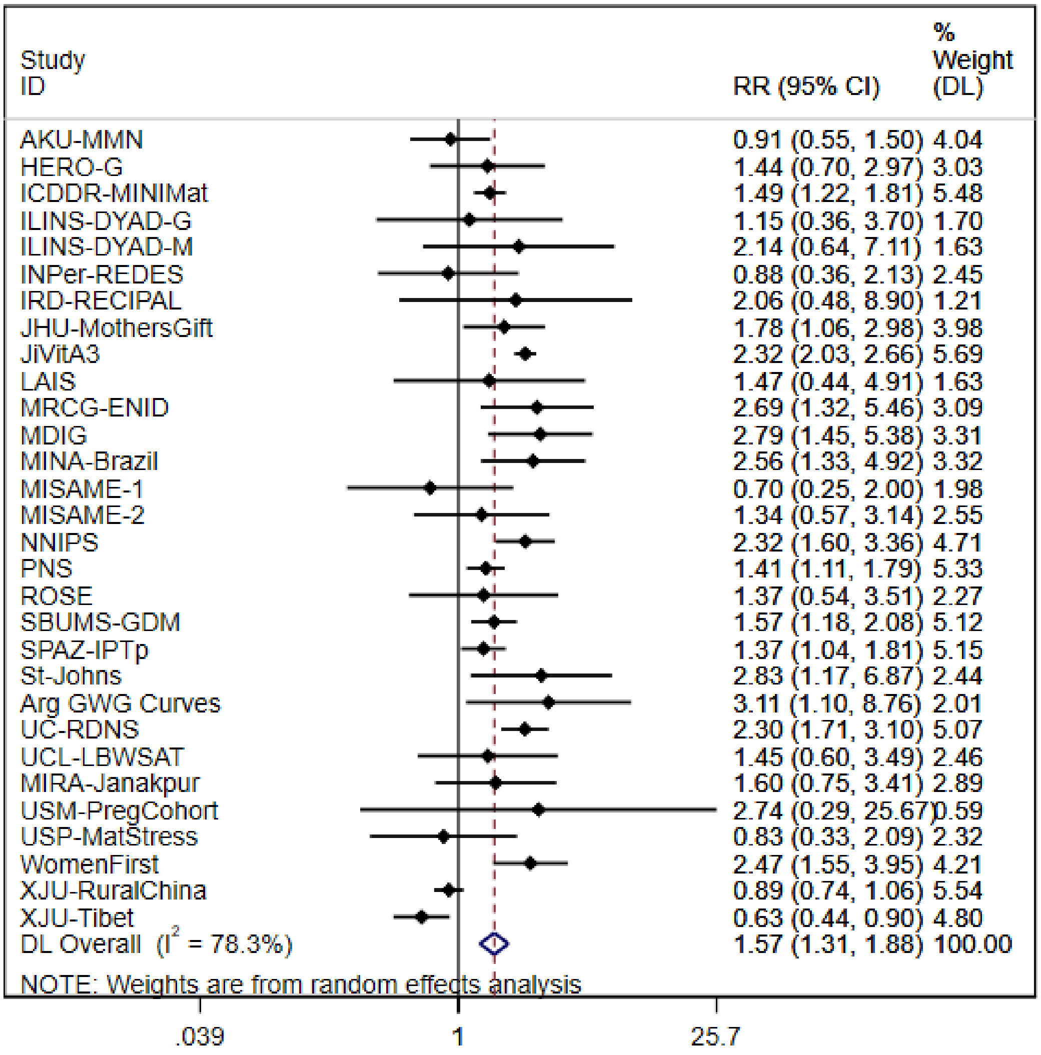

Supplemental Figure 9b: Forest plots of the association between **microcephaly** and moderately inadequate gestational weight gain (70 to <90%).

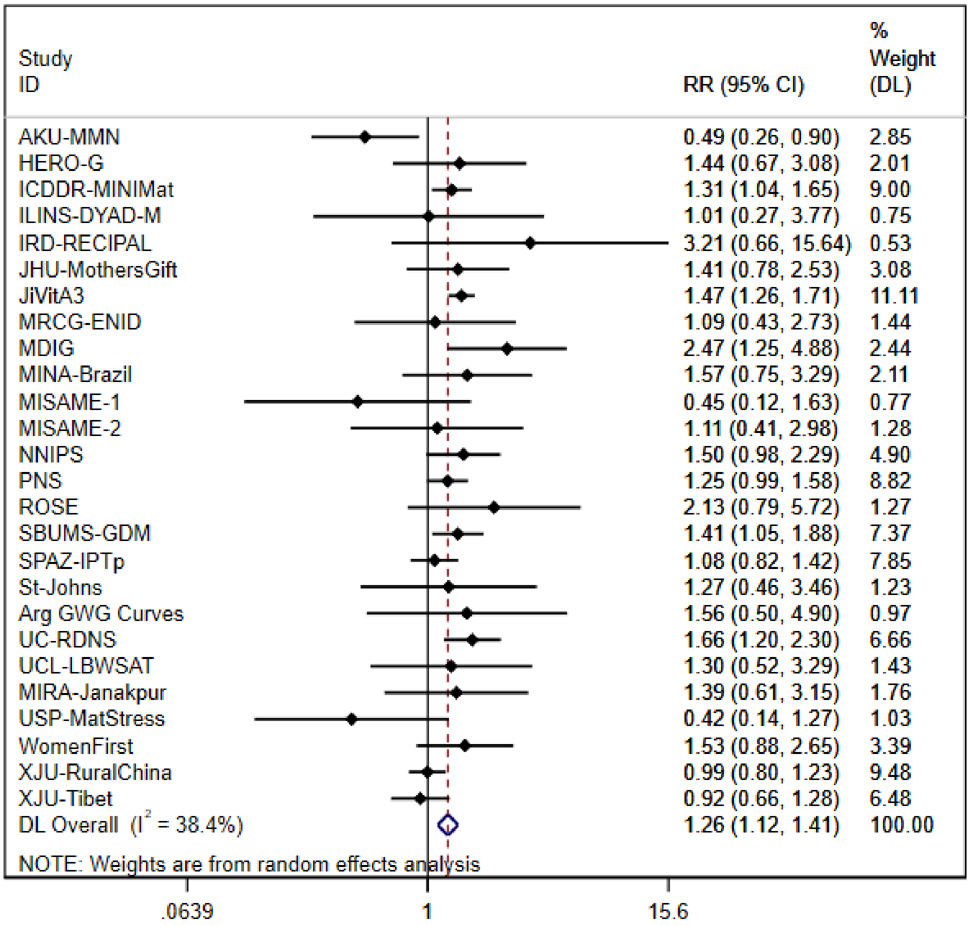

Supplemental Figure 9c: Forest plots of the association between **microcephaly** and excessive gestational weight gain (≥125%).

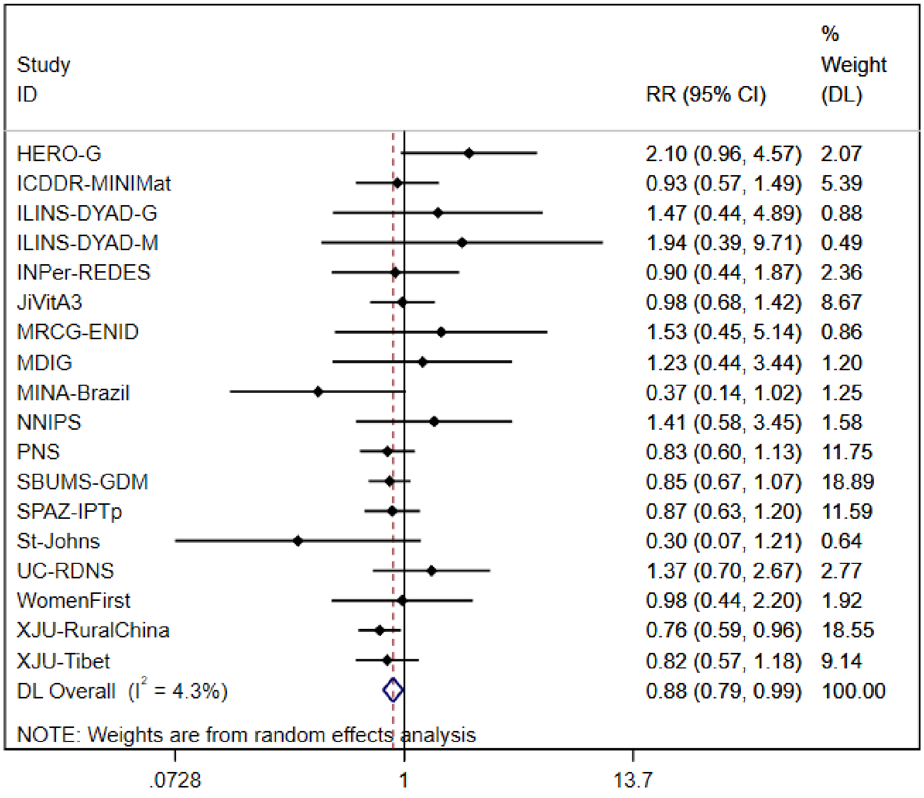

Supplemental Figure 10a: Forest plots of the association between **macrosomia** and severely inadequate gestational weight gain (<70%).

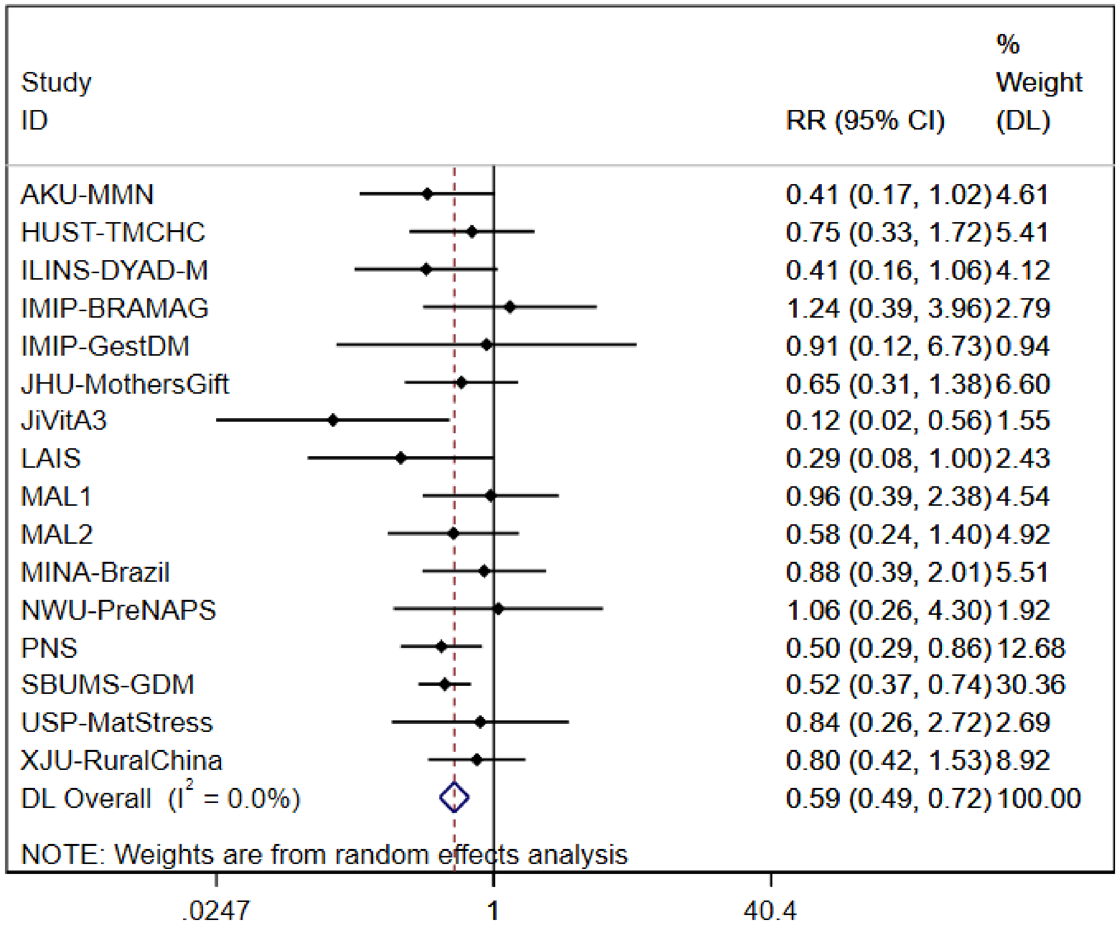

Supplemental Figure 10b: Forest plots of the association between **macrosomia** and moderately inadequate gestational weight gain (70 to <90%).

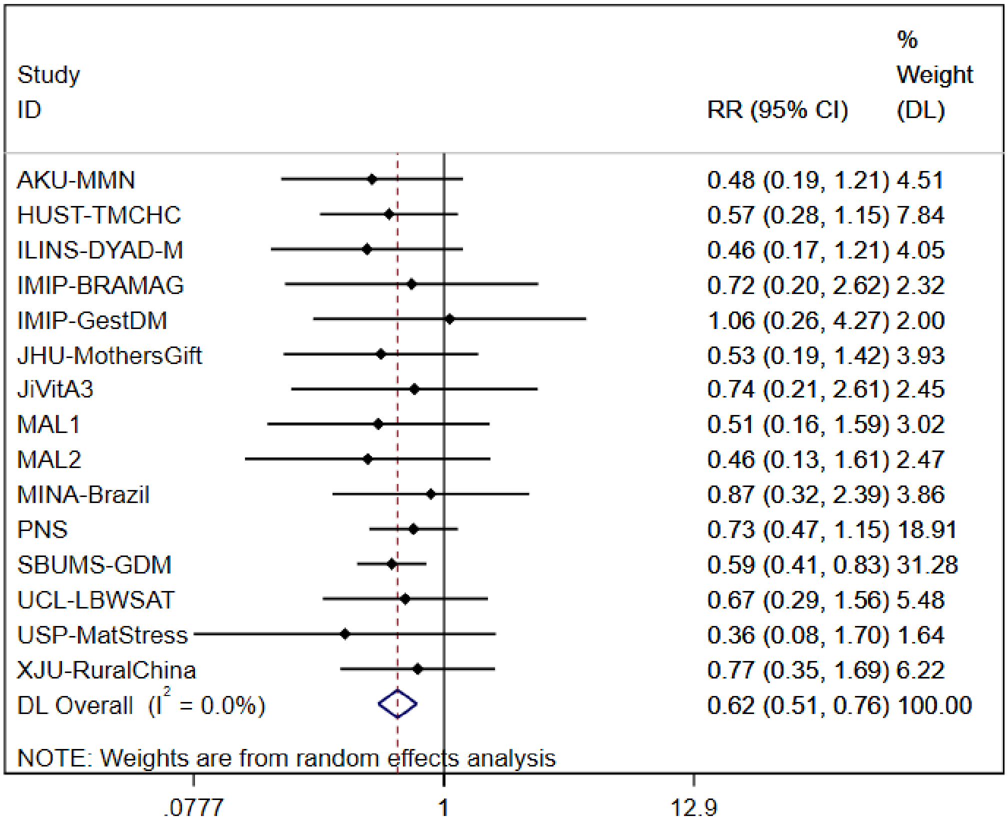

Supplemental Figure 10c: Forest plots of the association between **macrosomia** and excessive gestational weight gain (≥125%).

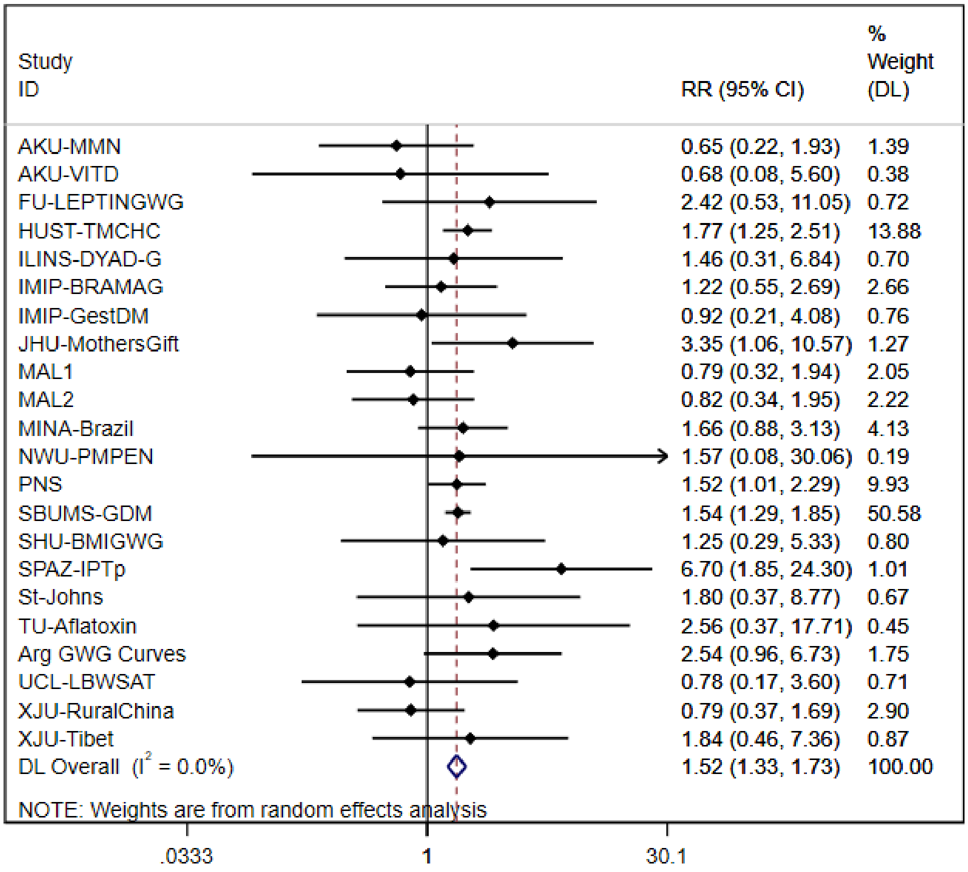

Supplemental Figure 11a: Forest plots of the association between **preterm birth** and severely inadequate gestational weight gain z-scores (<-2 SD).

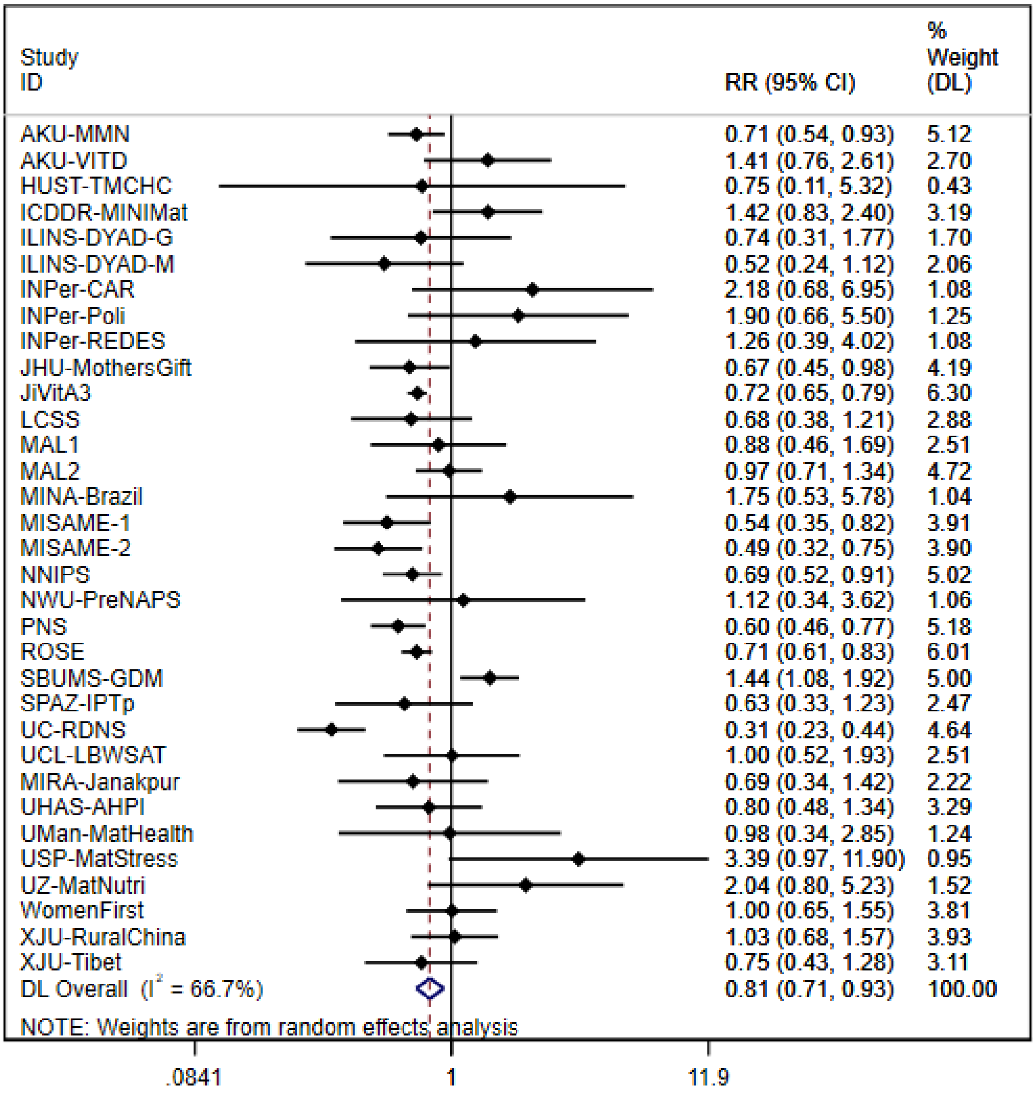

Supplemental Figure 11b: Forest plots of the association between **preterm birth** and inadequate gestational weight gain z-scores (-2 SD to <-1 SD).

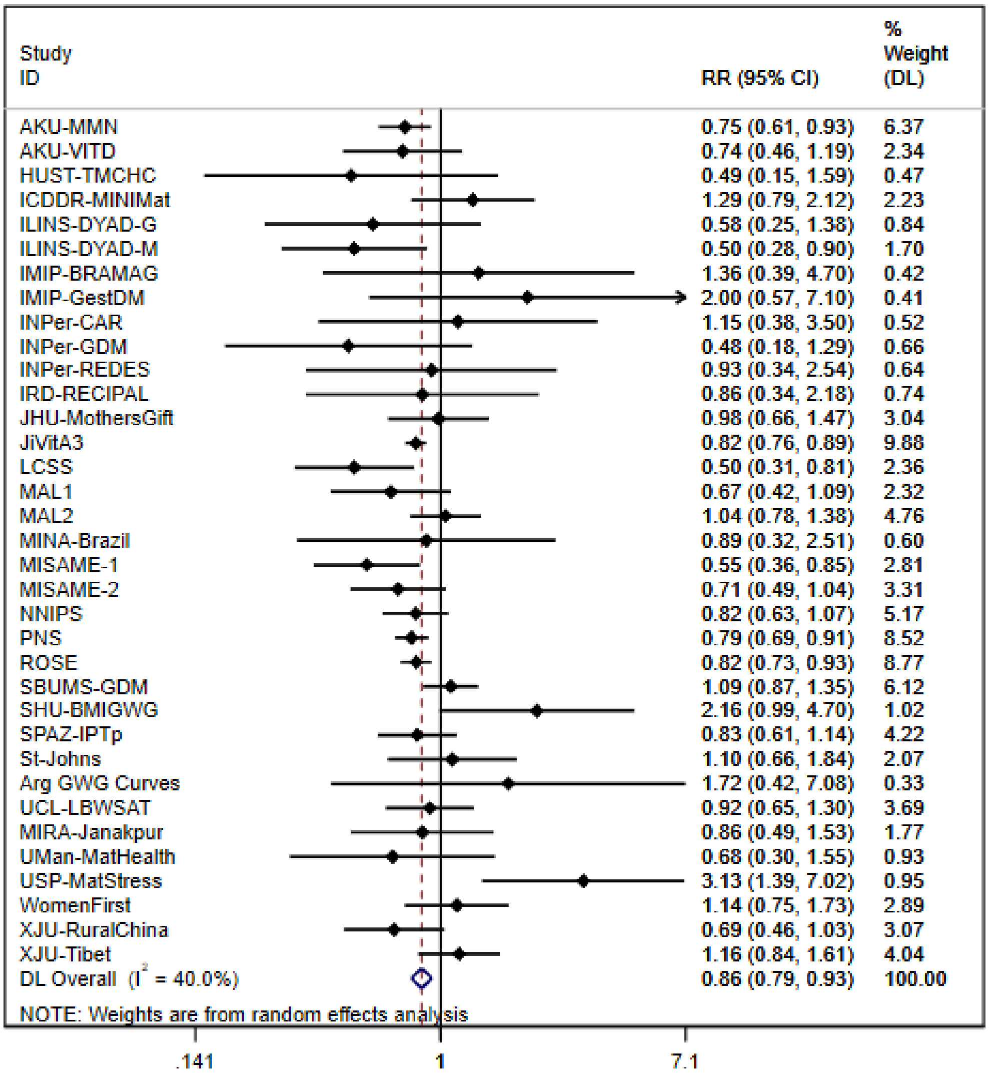

Supplemental Figure 11c: Forest plots of the association between **preterm birth** and excessive gestational weight gain z-scores (≥1 SD).

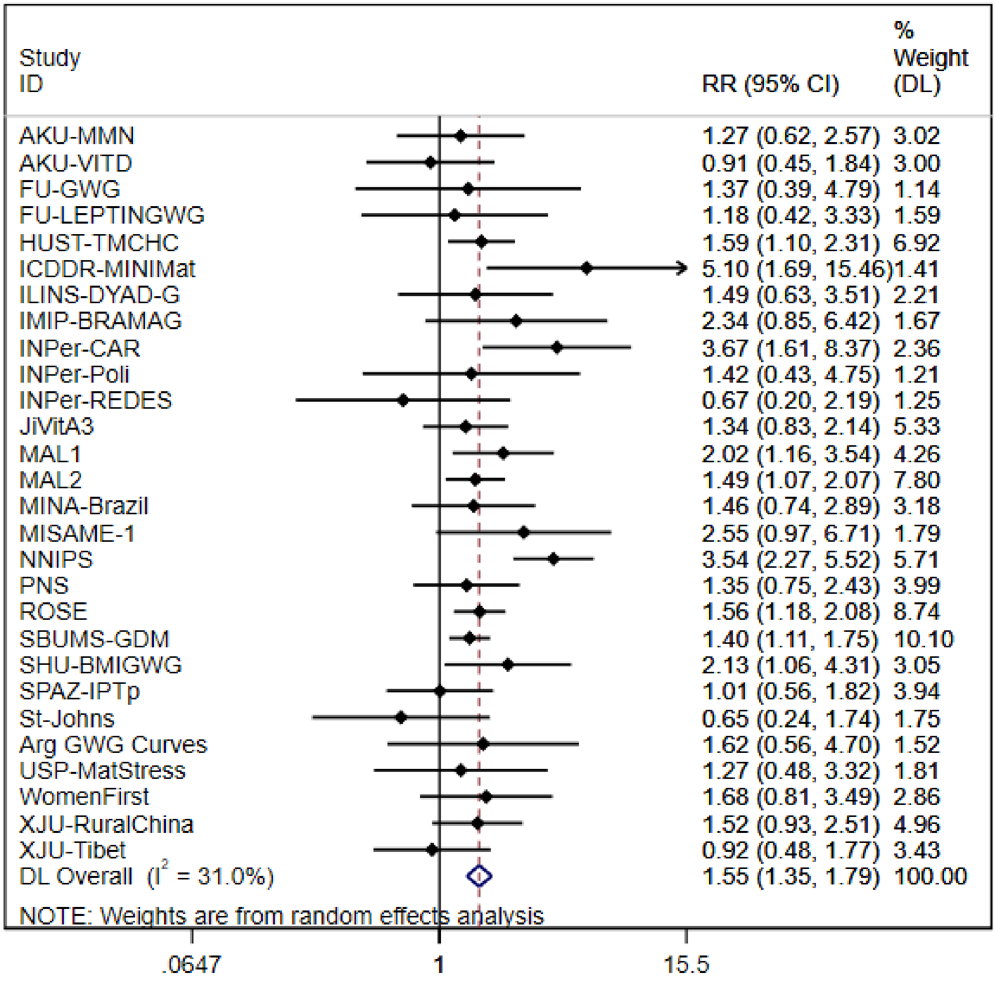

Supplemental Figure 12a: Forest plots of the association between **low birthweight** and severely inadequate gestational weight gain z-scores (<-2 SD).

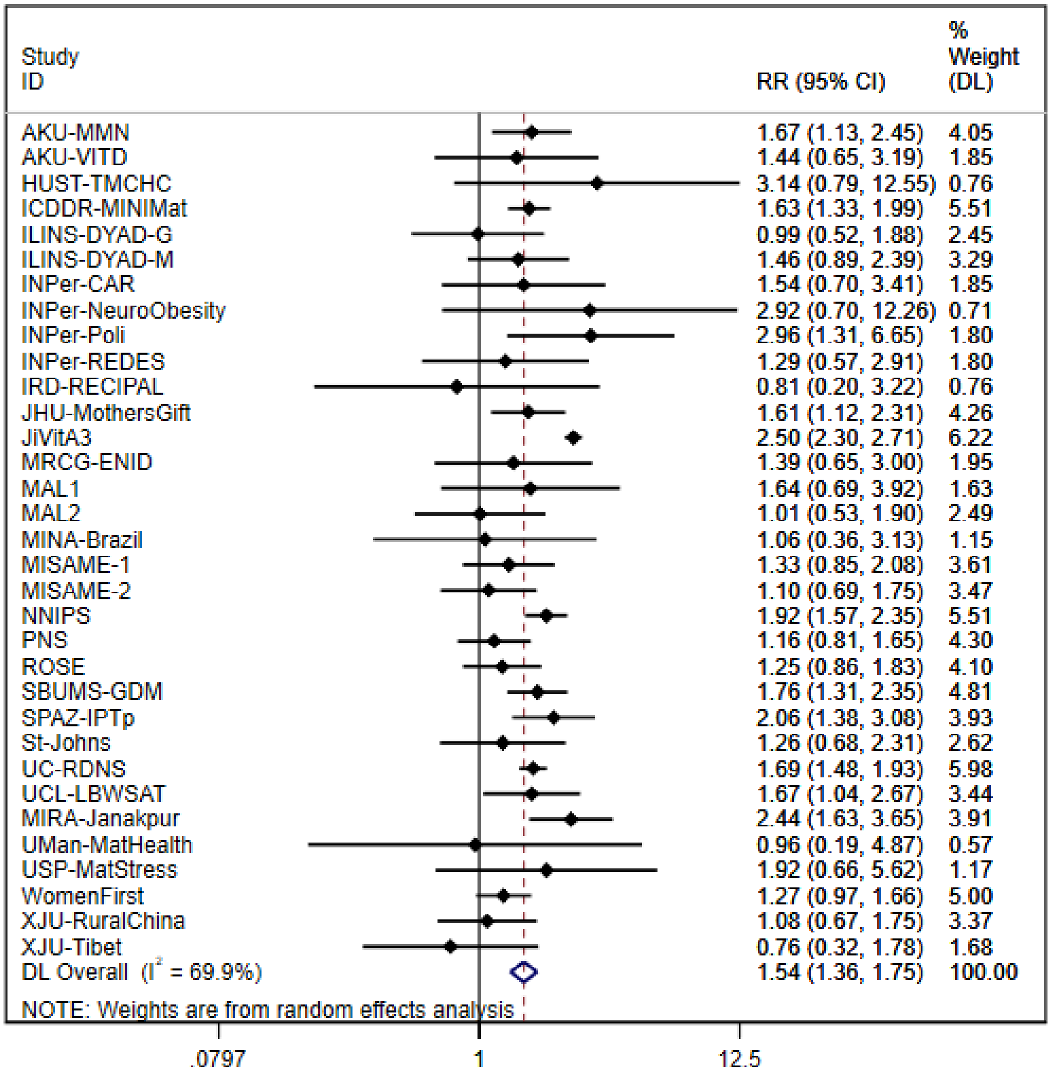

Supplemental Figure 12b: Forest plots of the association between **low birthweight** and inadequate gestational weight gain z-scores (-2 SD to <-1 SD).

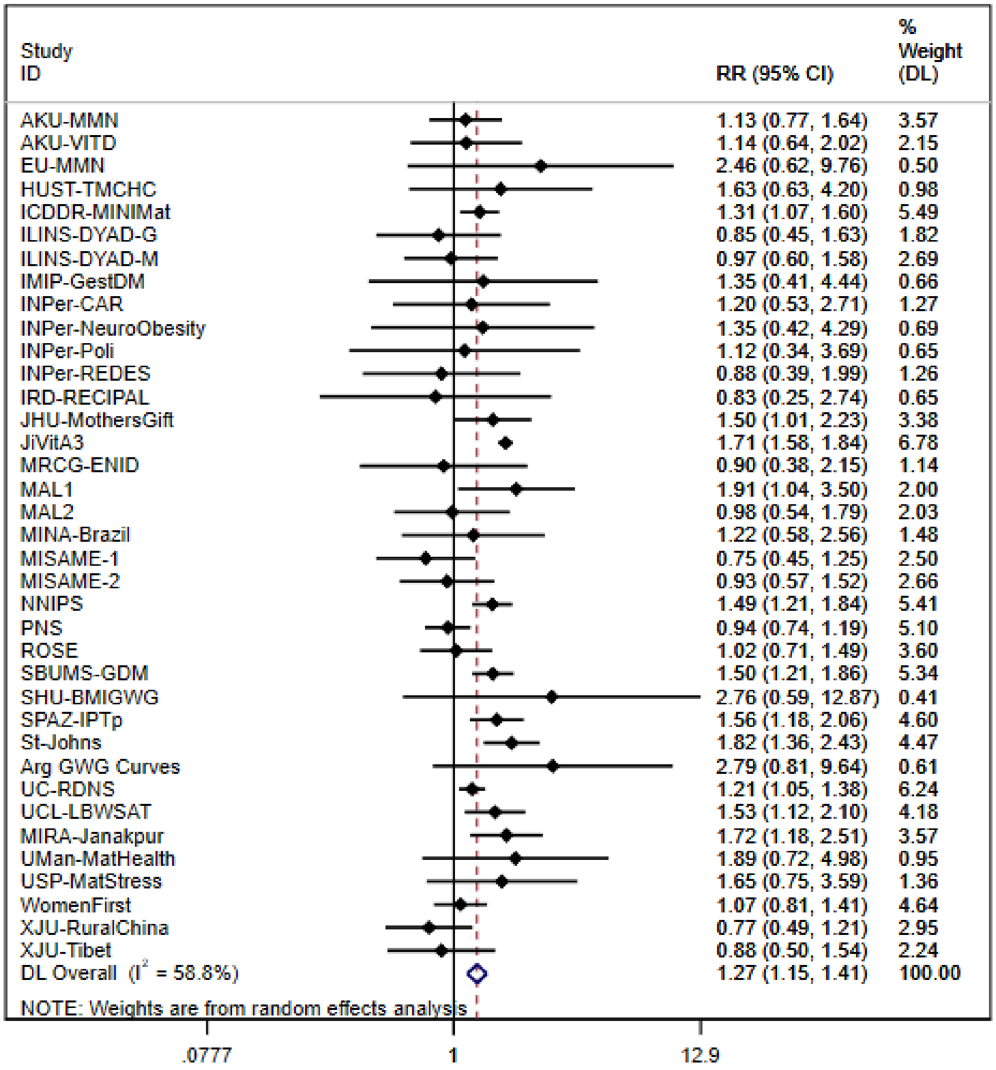

Supplemental Figure 12c: Forest plots of the association between **low birthweight** and excessive gestational weight gain z-scores (≥1 SD).

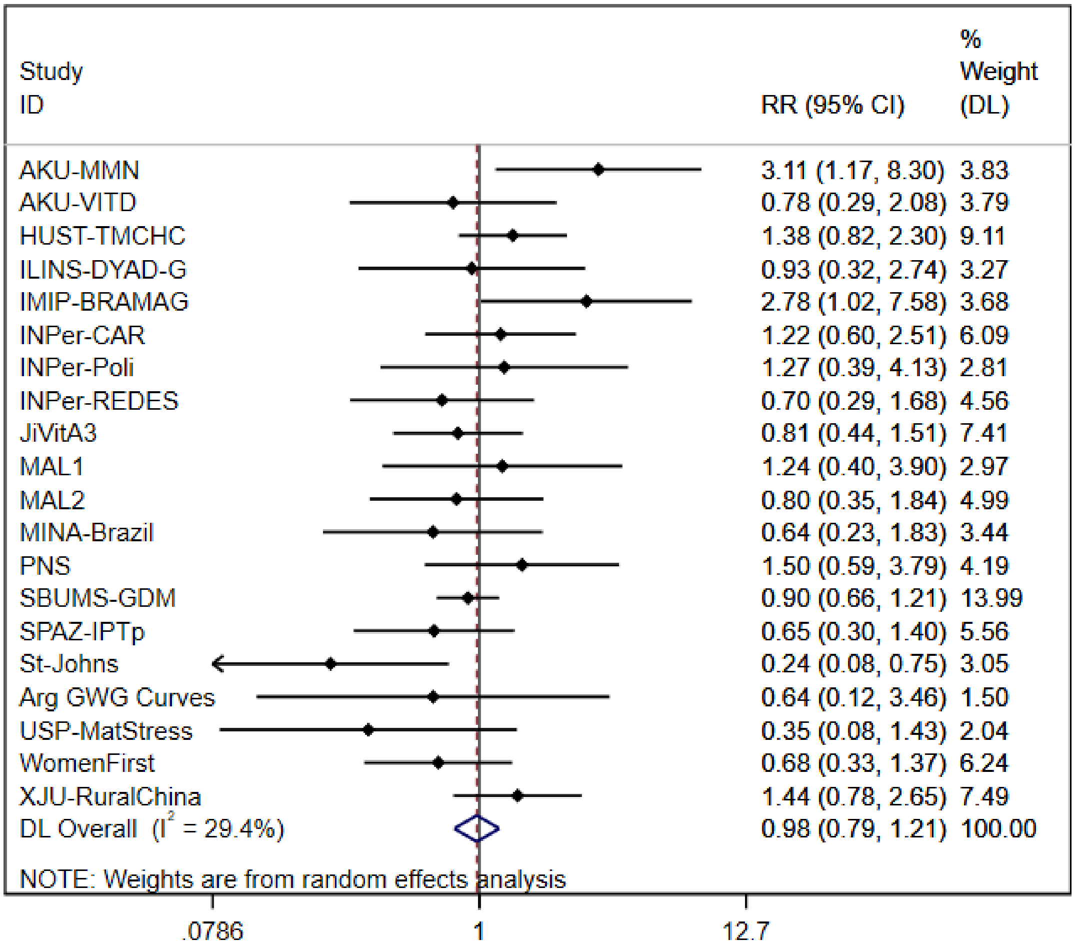

Supplemental Figure 13a: Forest plots of the association between **small-for-gestational age** and severely inadequate gestational weight gain z-scores (<-2 SD).

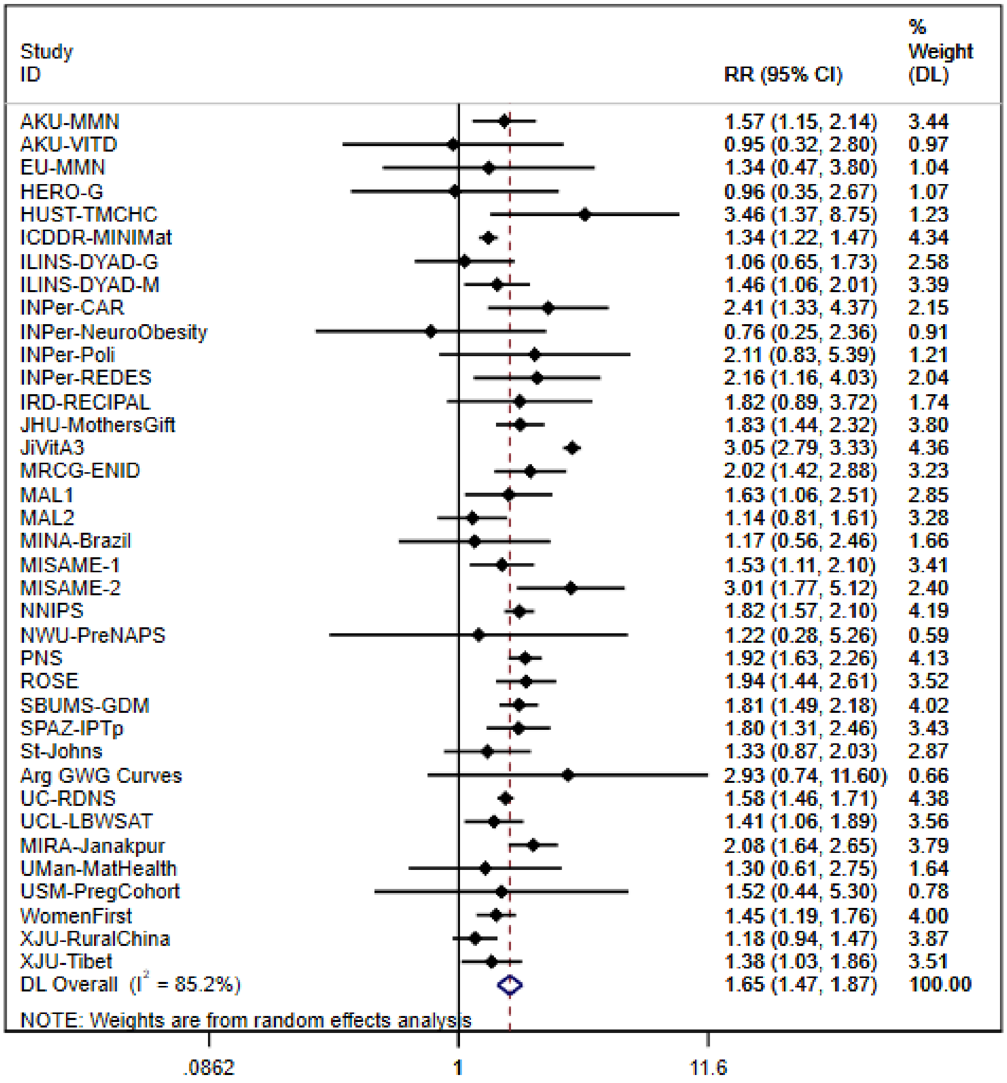

Supplemental Figure 13b: Forest plots of the association between **small-for-gestational age** and inadequate gestational weight gain z-scores (-2 SD to <-1 SD).

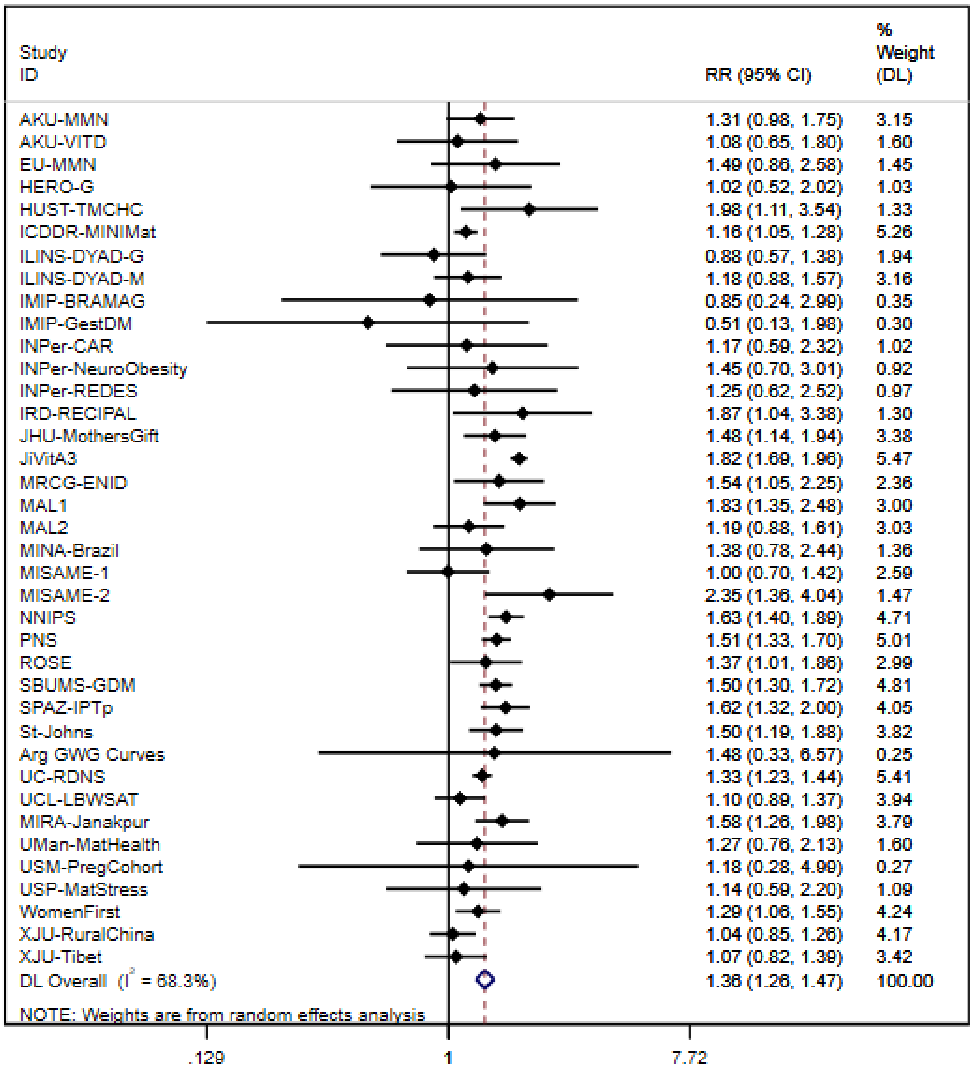

Supplemental Figure 13c: Forest plots of the association between **small-for-gestational age** and excessive gestational weight gain z-scores (≥1 SD).

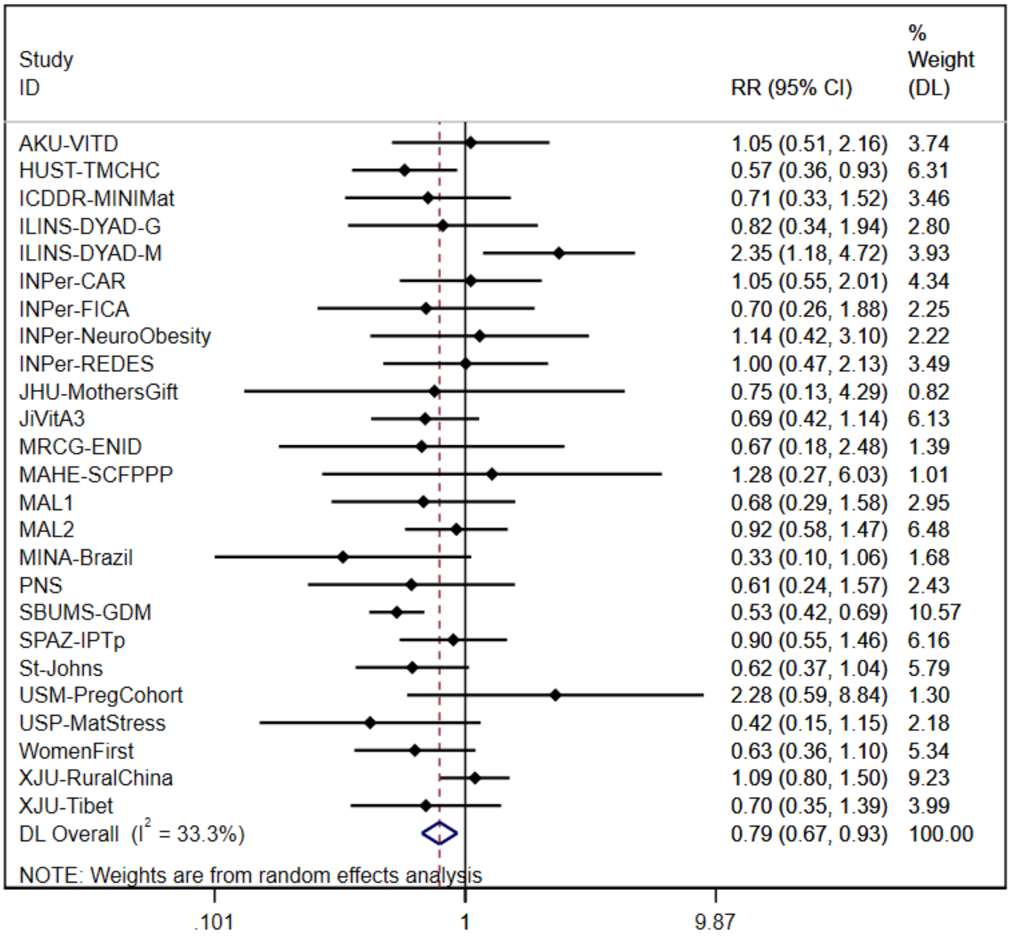

Supplemental Figure 14a: Forest plots of the association between **large-for-gestational age** and severely inadequate gestational weight gain z-scores (<-2 SD).

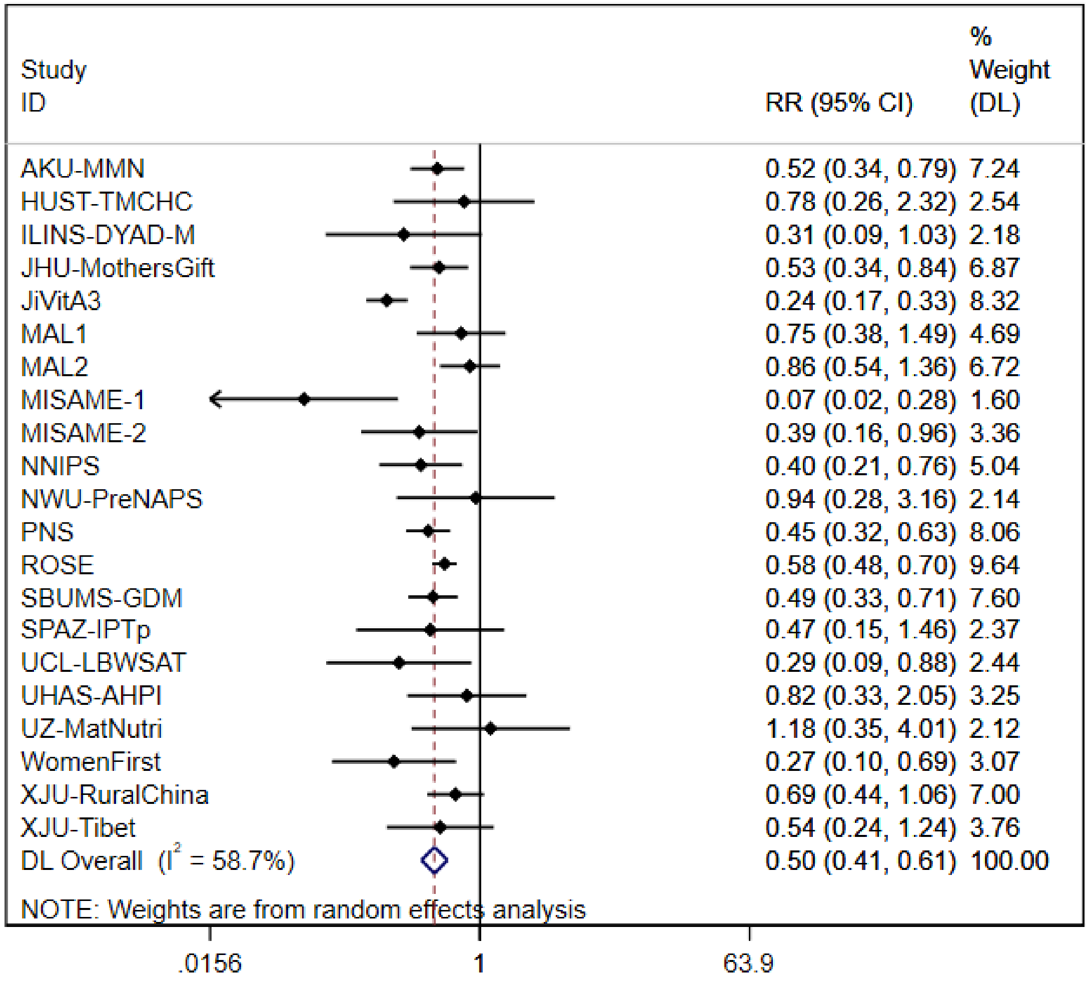

Supplemental Figure 14b: Forest plots of the association between **large-for-gestational age** and inadequate gestational weight gain z-scores (-2 SD to <-1 SD).

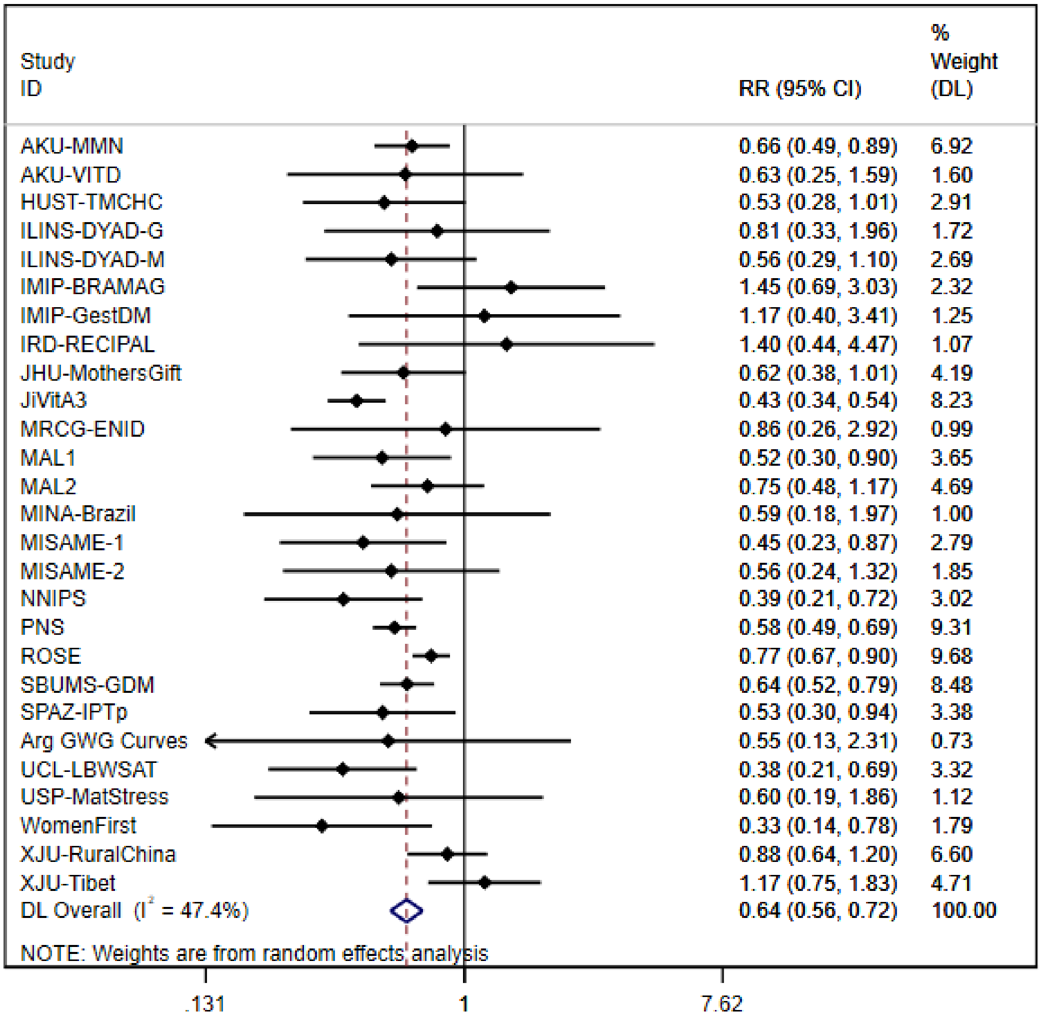

Supplemental Figure 14c: Forest plots of the association between **large-for-gestational age** and excessive gestational weight gain z-scores (≥1 SD).

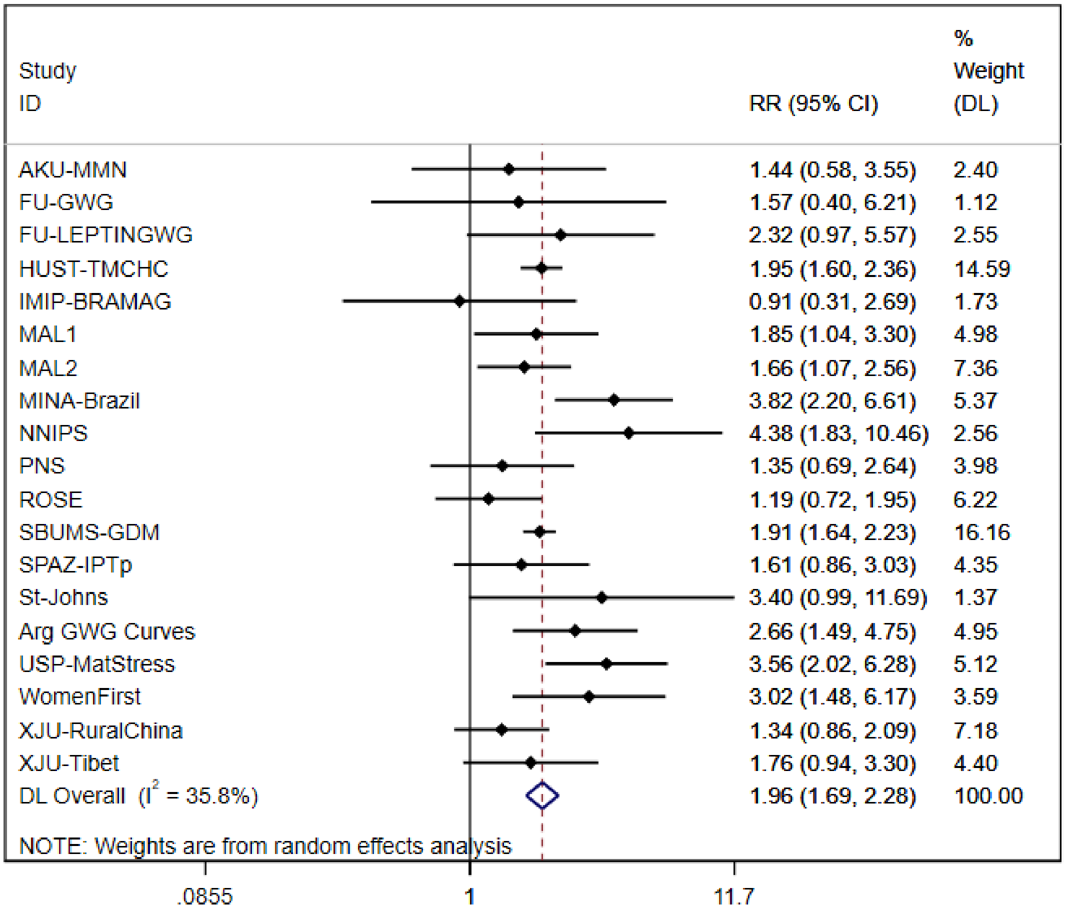

Supplemental Figure 15a: Forest plots of the association between **stillbirth** and severely inadequate gestational weight gain z-scores (<-2 SD).

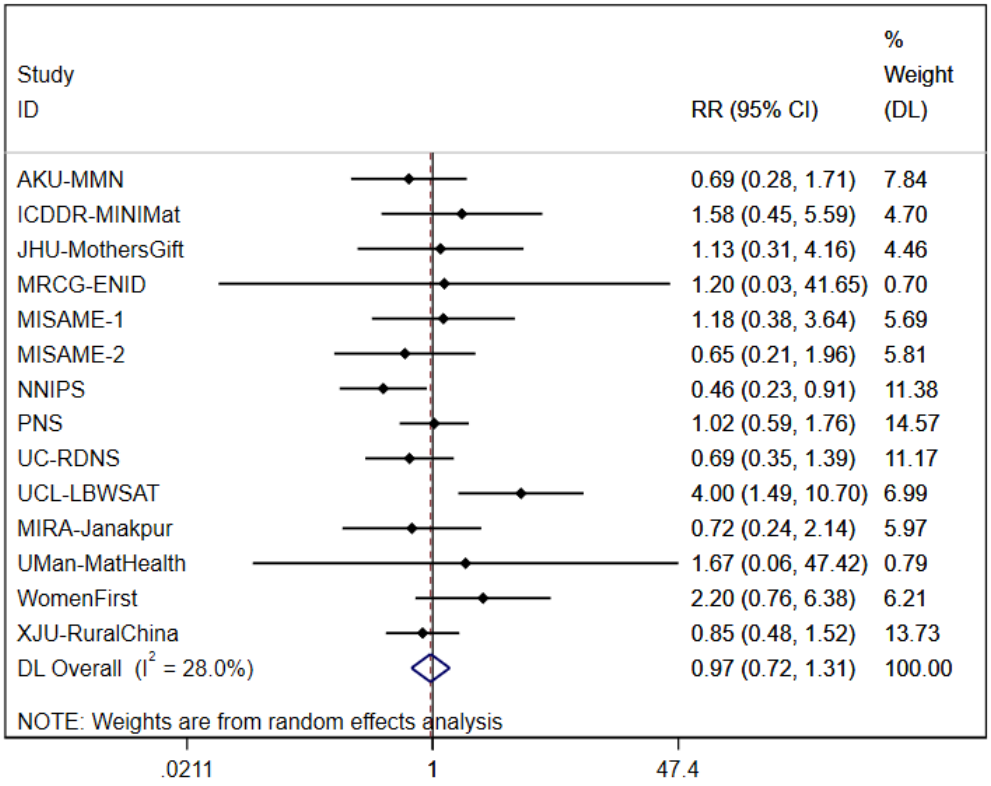

Supplemental Figure 15b: Forest plots of the association between **stillbirth** and inadequate gestational weight gain z-scores (-2 SD to <-1 SD).

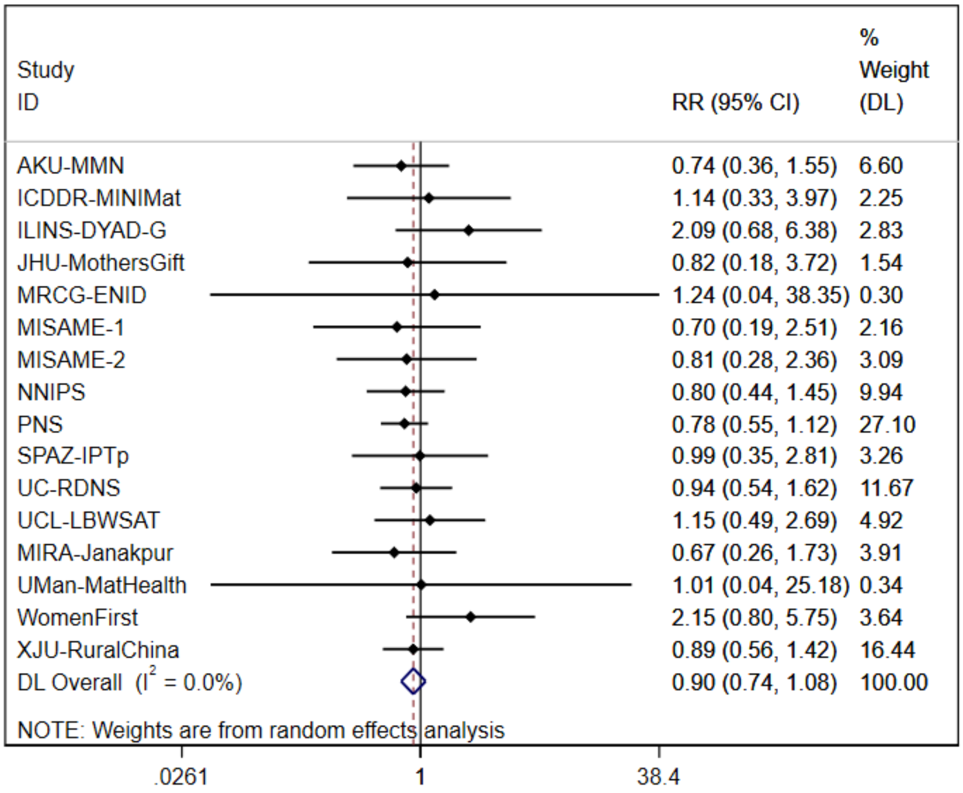

Supplemental Figure 15c: Forest plots of the association between **stillbirth** and excessive gestational weight gain z-scores (≥1 SD).

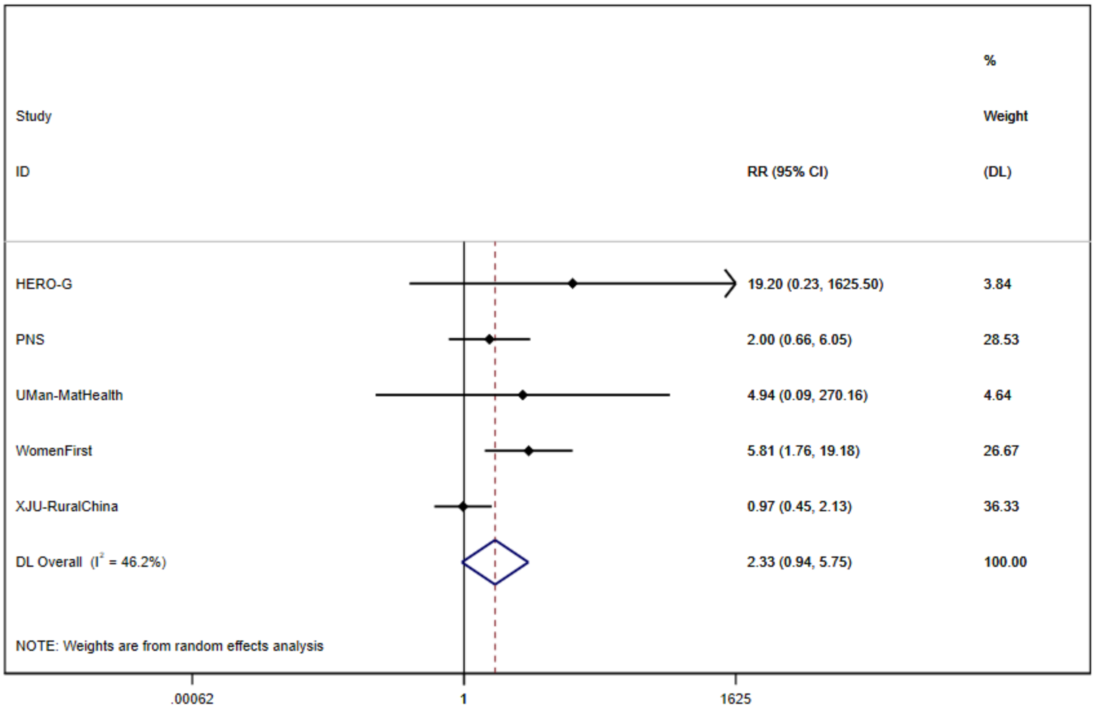

Supplemental Figure 16a: Forest plots of the association between **neonatal death** and severely inadequate gestational weight gain z-scores (<-2 SD).

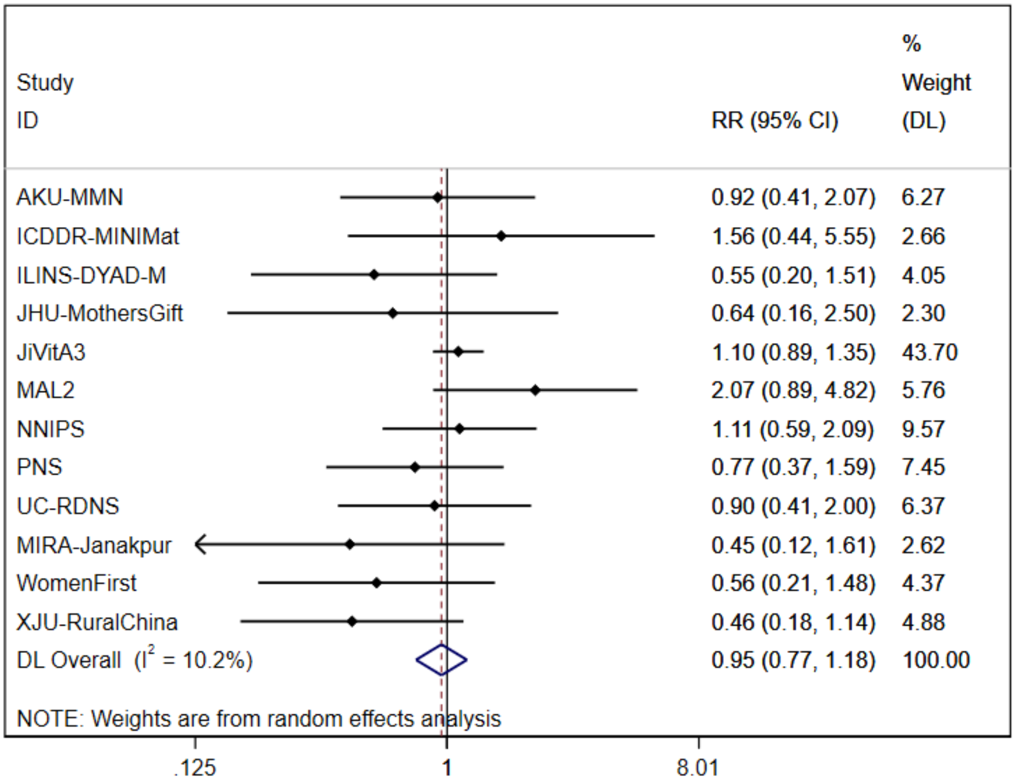

Supplemental Figure 16b: Forest plots of the association between **neonatal death** and inadequate gestational weight gain z-scores (-2 SD to <-1 SD).

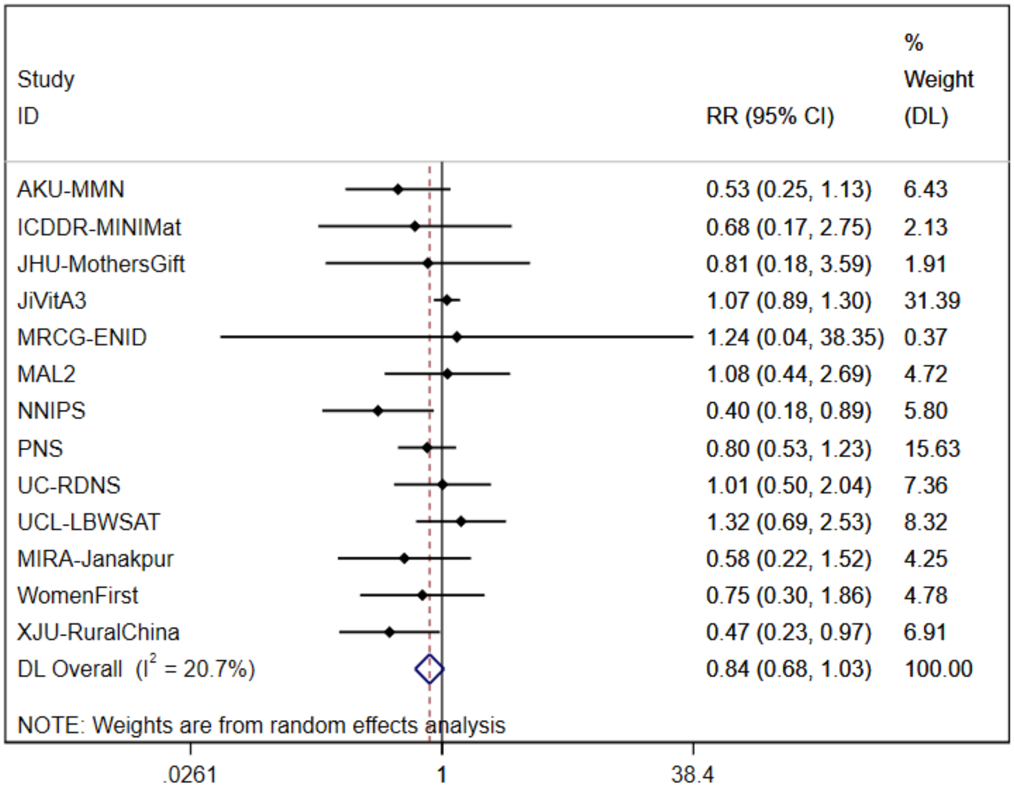

Supplemental Figure 16c: Forest plots of the association between **neonatal death** and excessive gestational weight gain z-scores (≥1 SD).

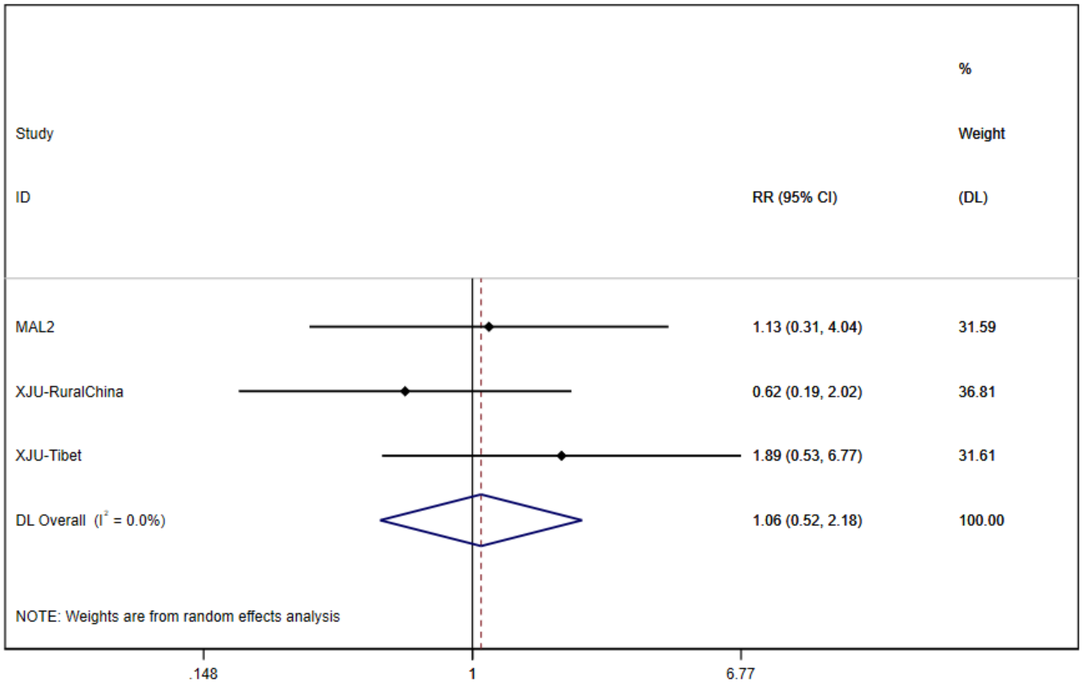

Supplemental Figure 17a: Forest plots of the association between **short-for-gestational age** and severely inadequate gestational weight gain z-scores (<-2 SD).

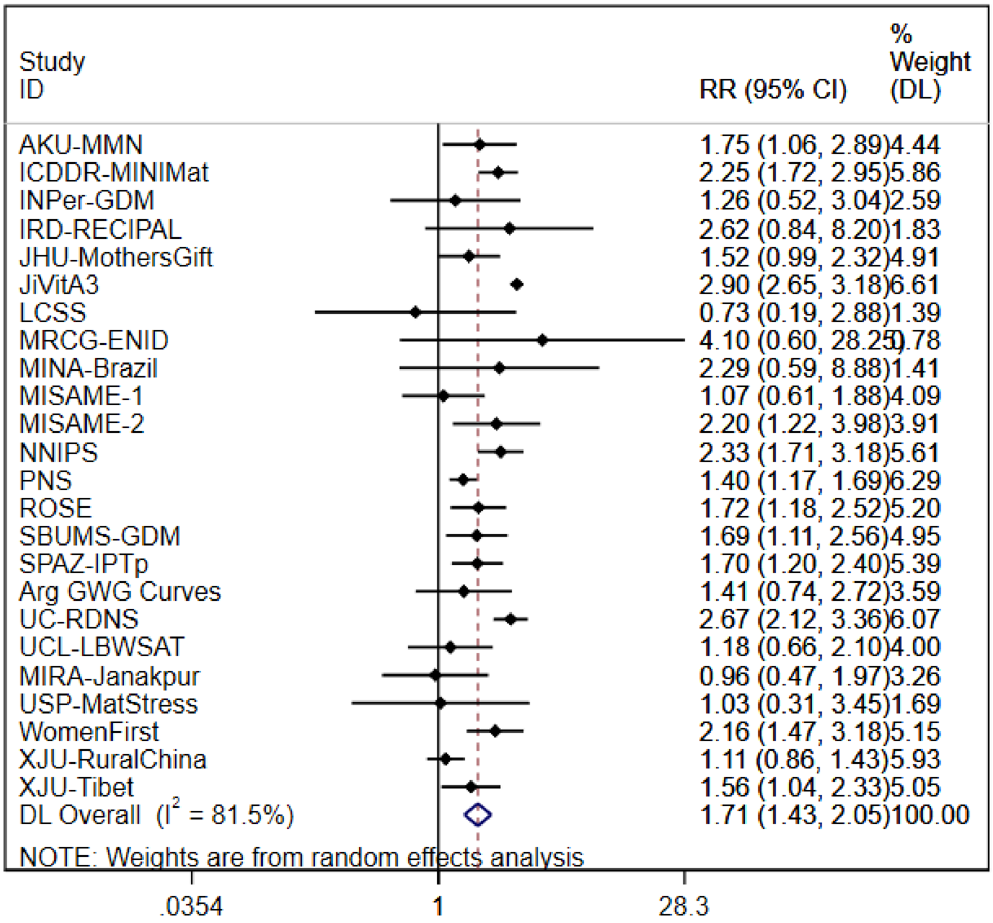

Supplemental Figure 17b: Forest plots of the association between **short-for-gestational age** and inadequate gestational weight gain z-scores (-2 SD to <-1 SD).

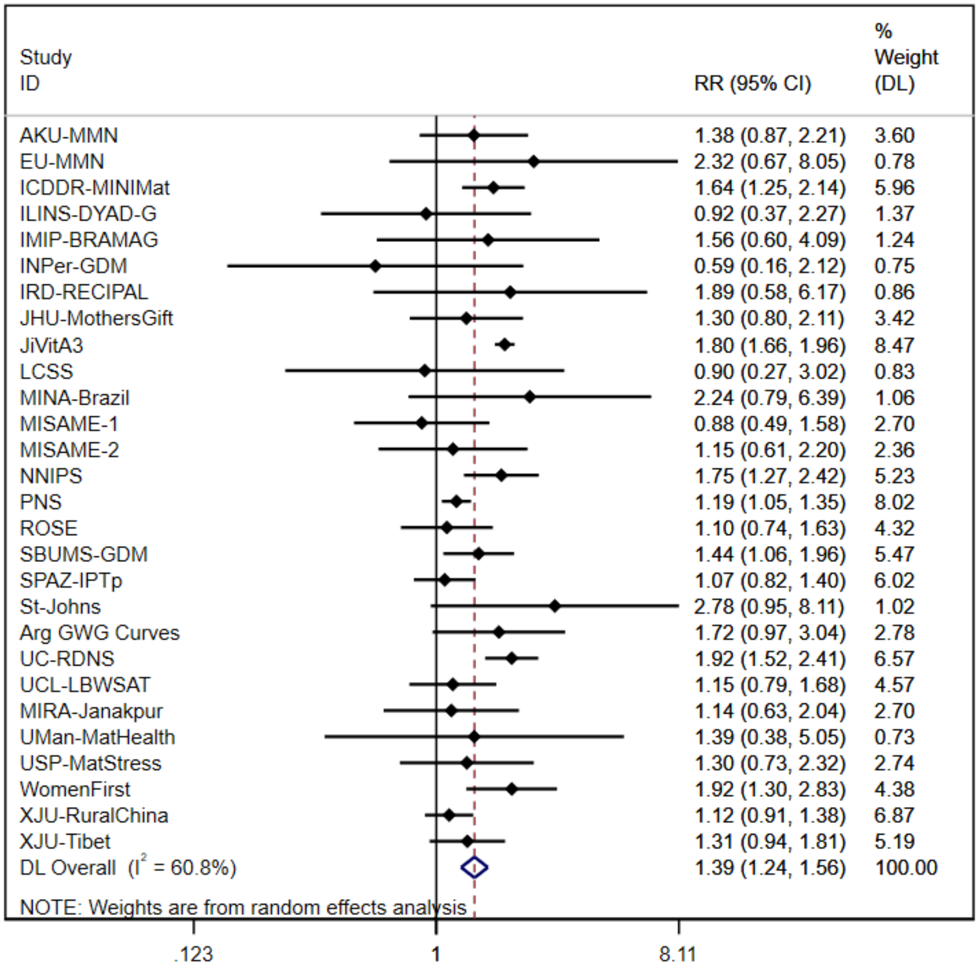

Supplemental Figure 17c: Forest plots of the association between **short-for-gestational age** and excessive gestational weight gain z-scores (≥1 SD).

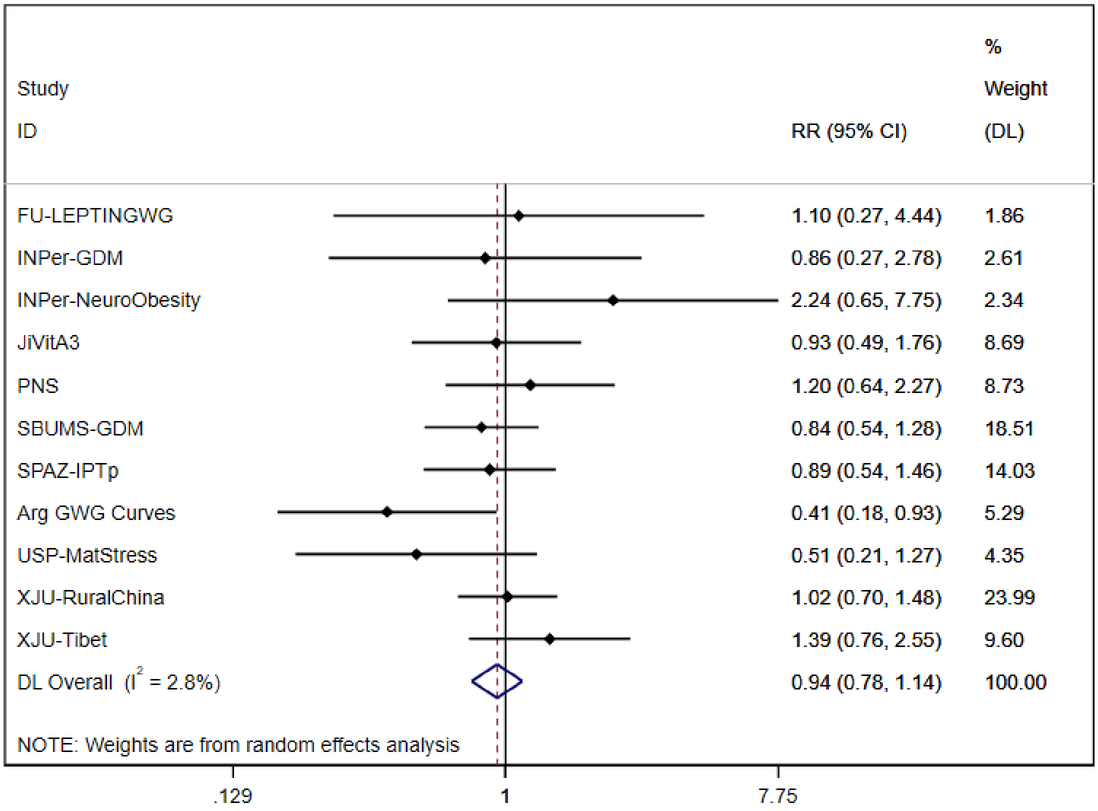

Supplemental Figure 18a: Forest plots of the association between **microcephaly** and severely inadequate gestational weight gain z-scores (<-2 SD).

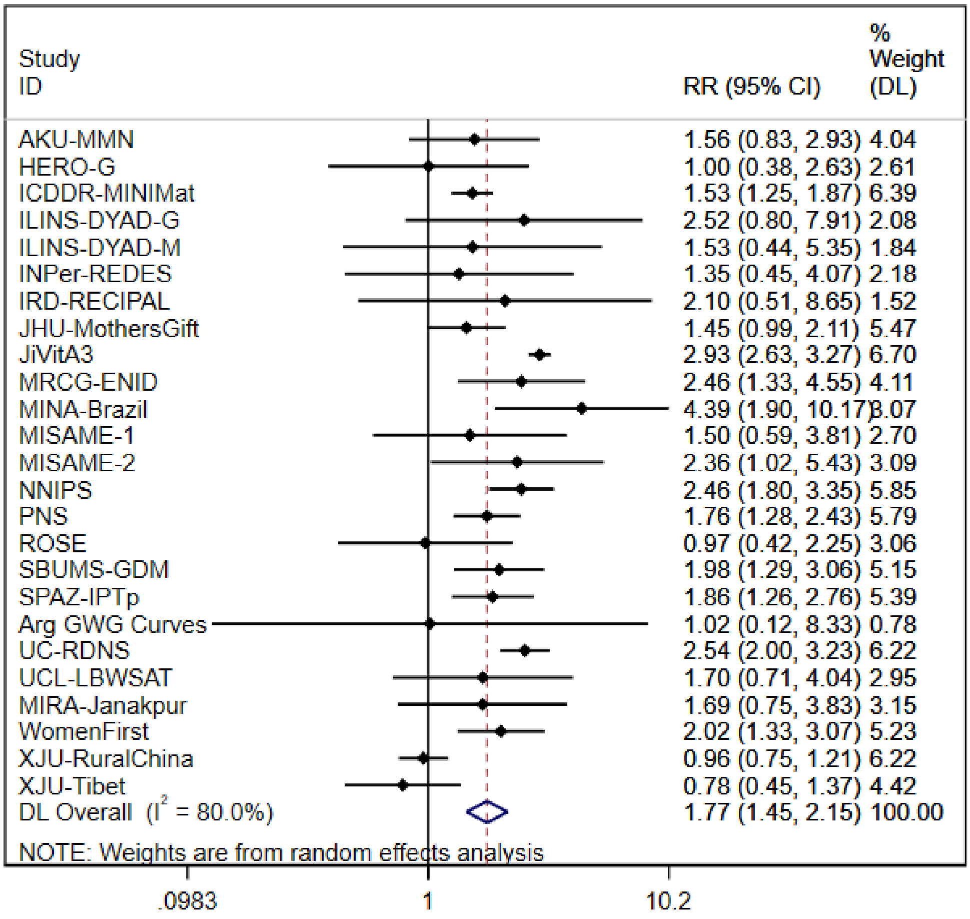

Supplemental Figure 18b: Forest plots of the association between **microcephaly** and inadequate gestational weight gain z-scores (-2 SD to <-1 SD).

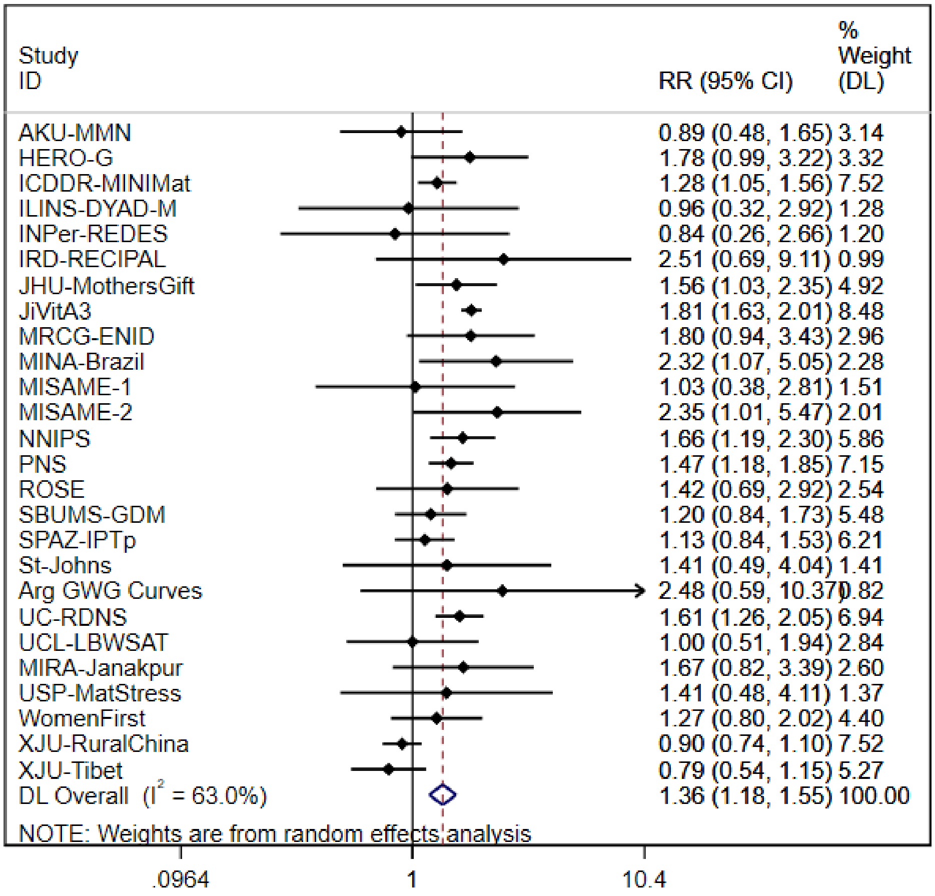

Supplemental Figure 18c: Forest plots of the association between **microcephaly** and excessive gestational weight gain z-scores (≥1 SD).

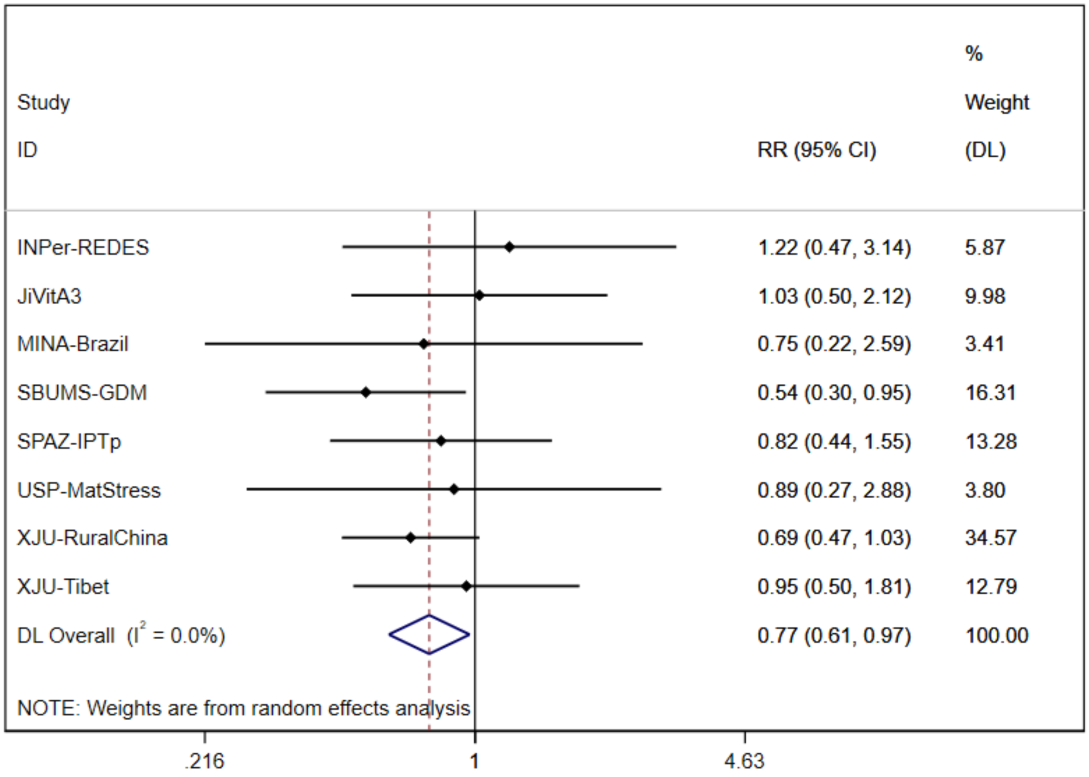

Supplemental Figure 19a: Forest plots of the association between **macrosomia** and severely inadequate gestational weight gain z-scores (<-2 SD).

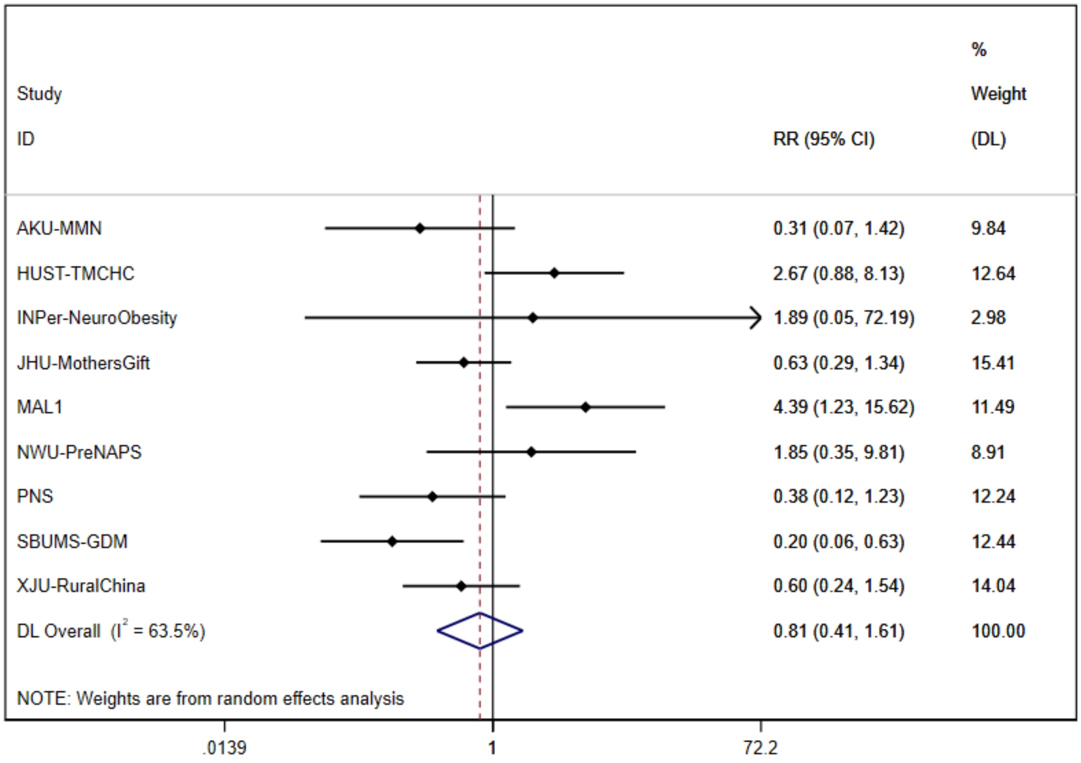

Supplemental Figure 19b: Forest plots of the association between **macrosomia** and inadequate gestational weight gain z-scores (-2 SD to <-1 SD).

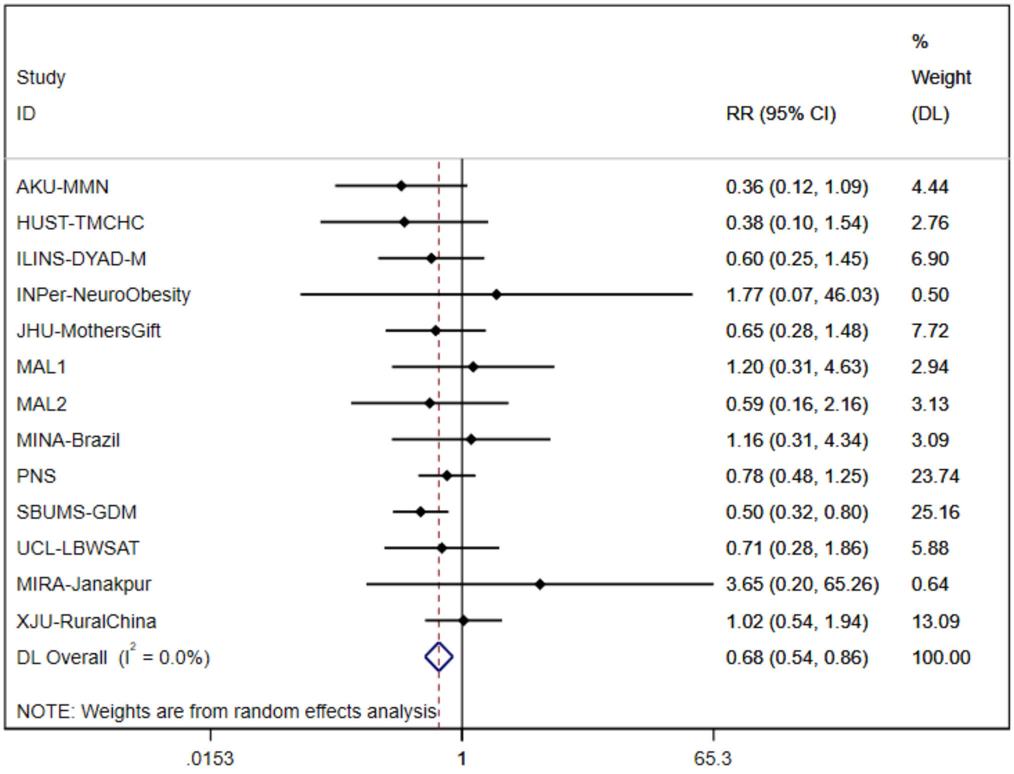

Supplemental Figure 19c: Forest plots of the association between **macrosomia** and excessive gestational weight gain z-scores (≥1 SD).

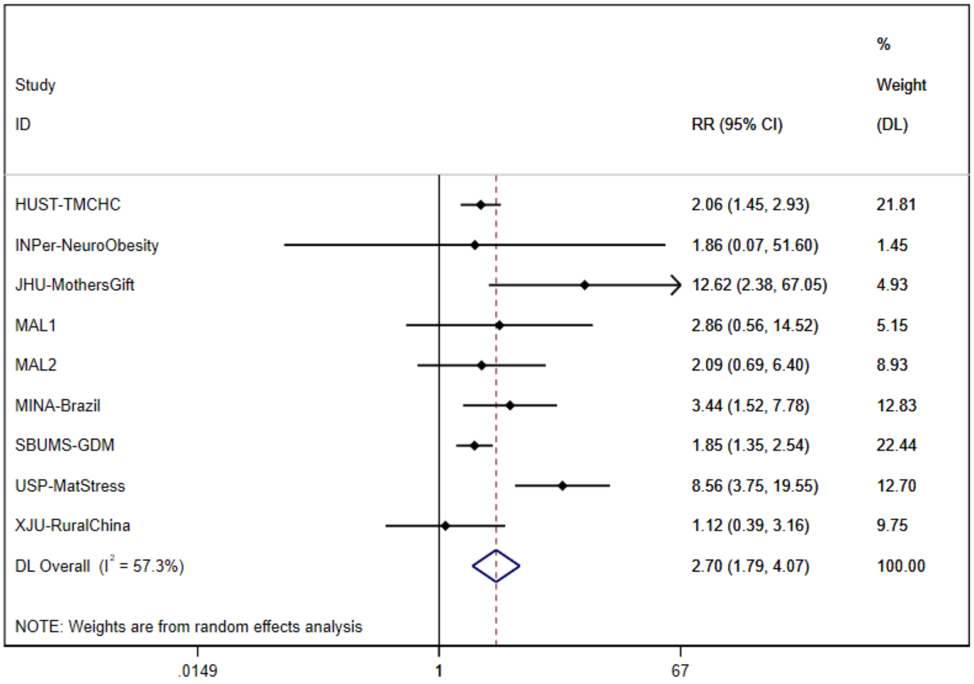

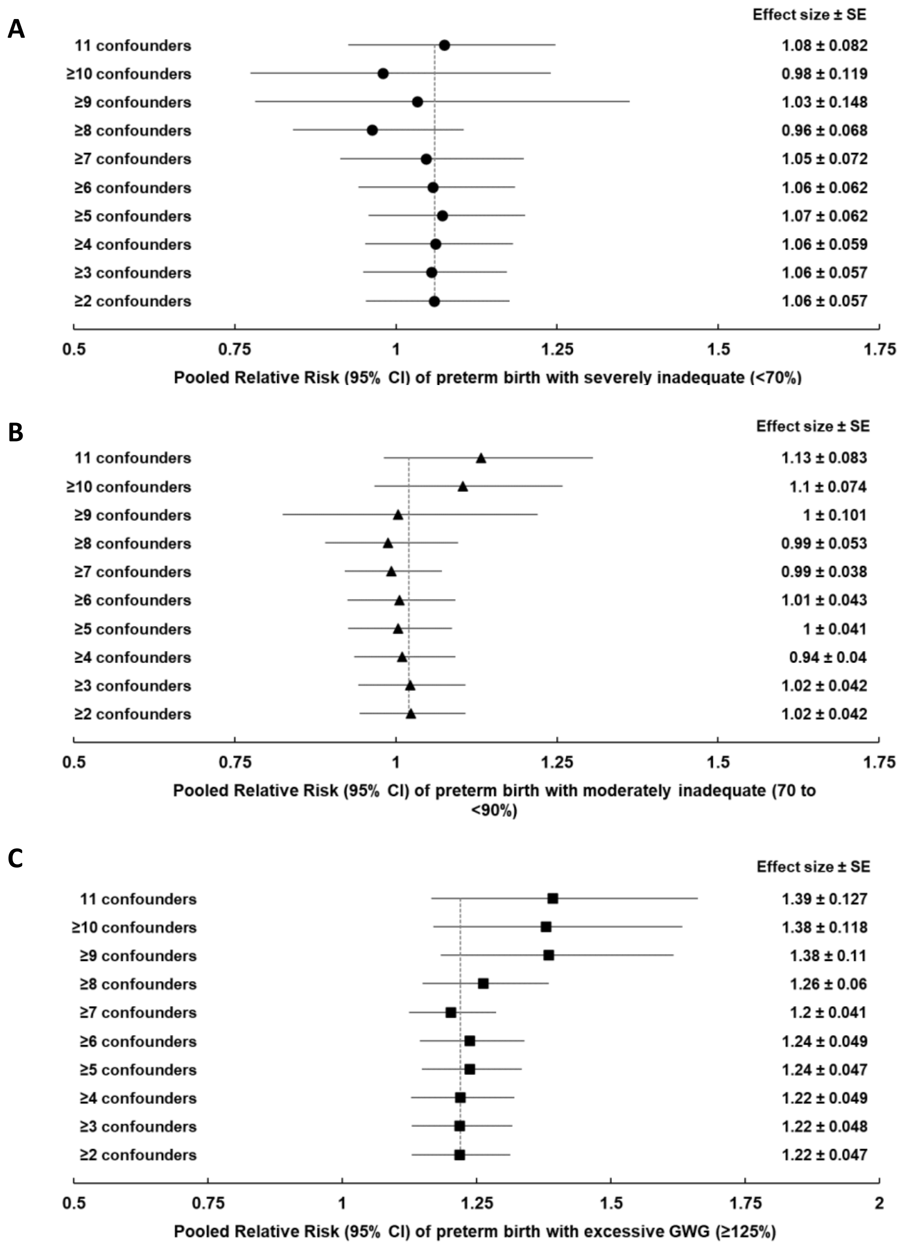

Supplementary Figure 20: Pooled relative risk (RR) and 95% CI of the associations between severely inadequate gestational weight gain (GWG) (A), moderately inadequate GWG (B), and excessive GWG (C) compared to adequate GWG (90-<125%) with preterm birth using two-stage meta-analysis when studies with fewer confounders available for adjustment are included sequentially. The dashed vertical line in each panel is the pooled effect size in primary analyses using all studies and adjusting for all available confounders.

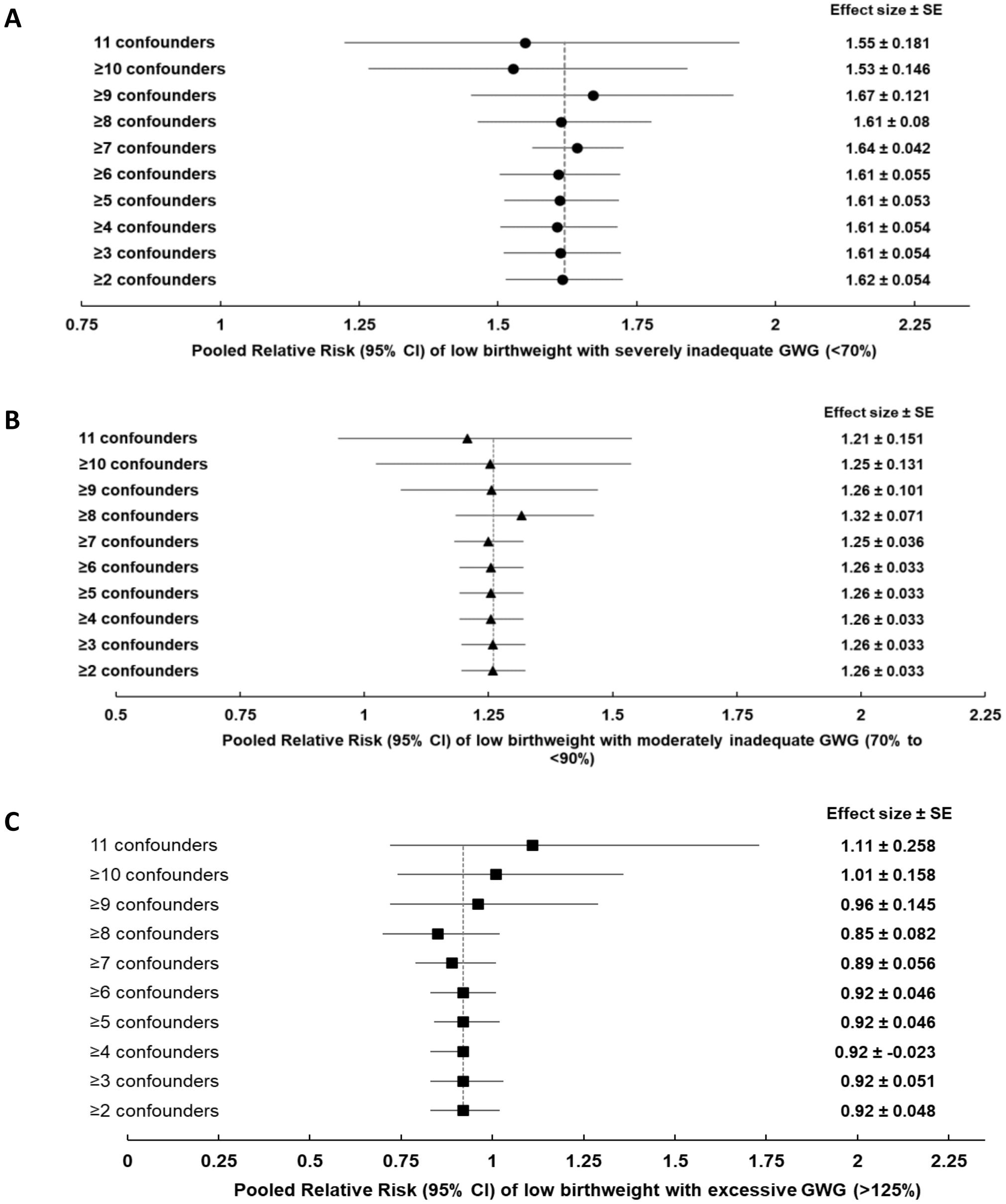

Supplementary Figure 21: Pooled relative risk (RR) and 95% CI of the associations between severely inadequate gestational weight gain (GWG) (A), moderately inadequate GWG (B), and excessive GWG (C) compared to adequate GWG (90-<125%) with low birthweight using two-stage meta-analysis when studies with fewer confounders available for adjustment are included sequentially. The dashed vertical line in each panel is the pooled effect size in primary analyses using all studies and adjusting for all available confounders.

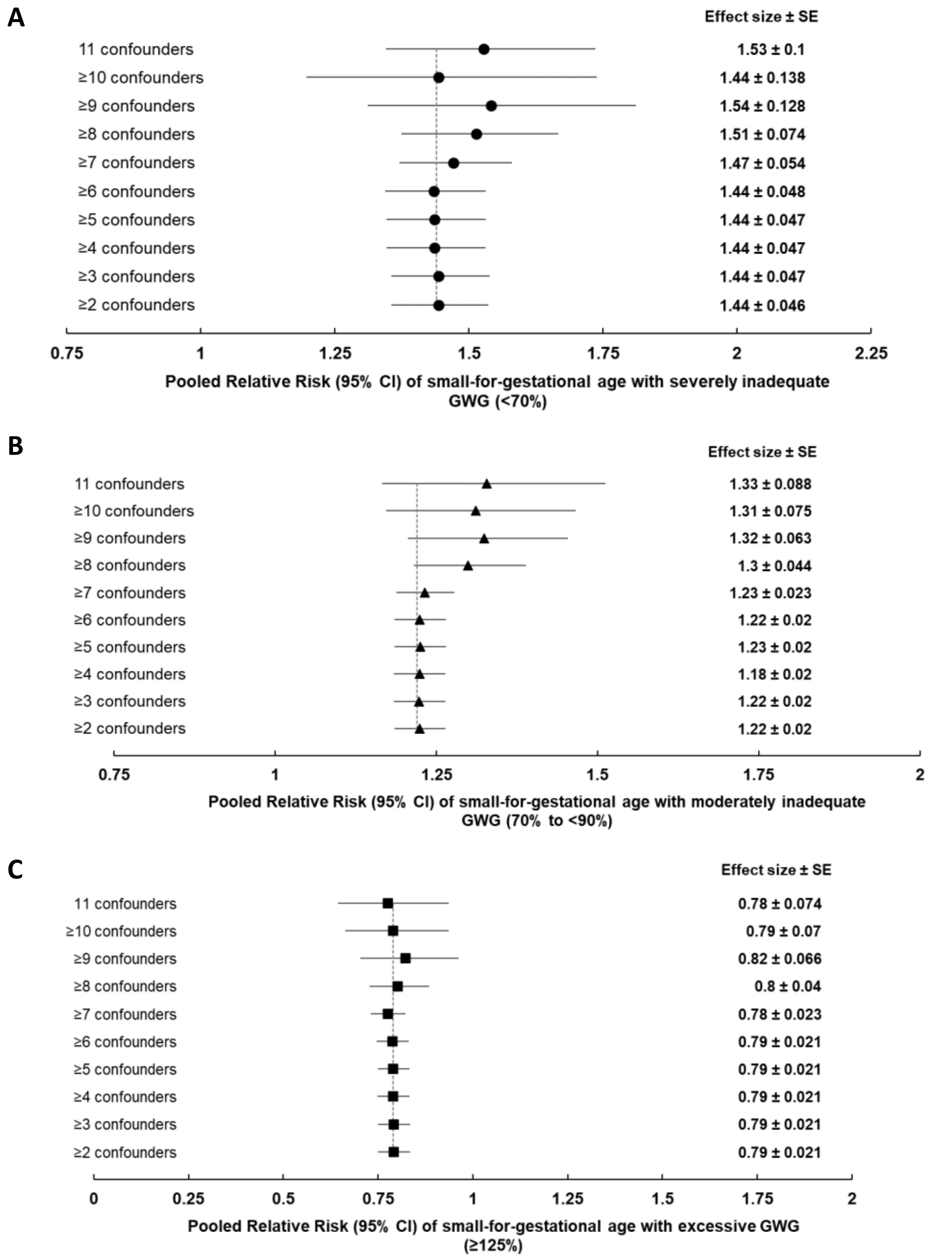

Supplementary Figure 22: Pooled relative risk (RR) and 95% CI of the associations between severely inadequate gestational weight gain (GWG) (A), moderately inadequate GWG (B), and excessive GWG (C) compared to adequate GWG (90-<125%) with small-for-gestational age using two-stage meta-analysis when studies with fewer confounders available for adjustment are included sequentially. The dashed vertical line in each panel is the pooled effect size in primary analyses using all studies and adjusting for all available confounders.

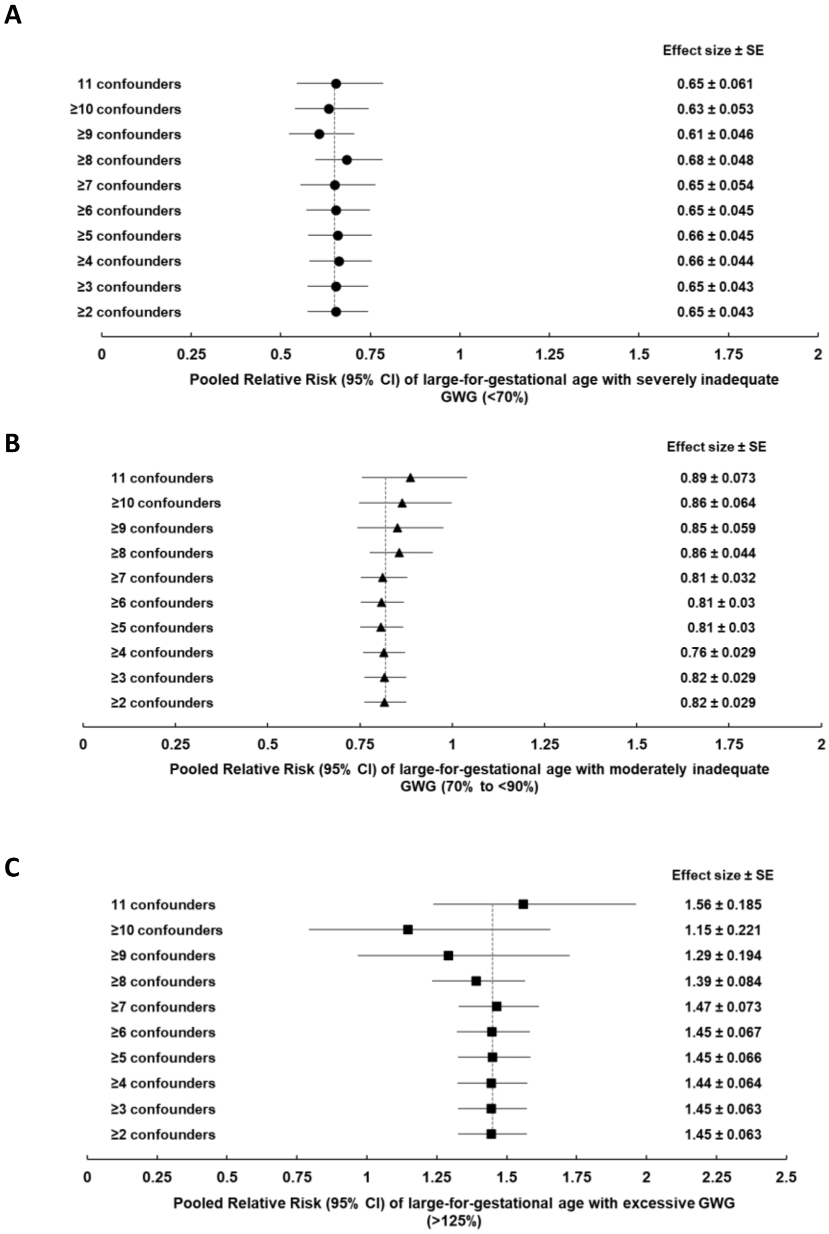

Supplementary Figure 23: Pooled relative risk (RR) and 95% CI of the associations between severely inadequate gestational weight gain (GWG) (A), moderately inadequate GWG (B), and excessive GWG (C) compared to adequate GWG (90-<125%) with large-for-gestational age using two-stage meta-analysis when studies with fewer confounders available for adjustment are included sequentially. The dashed vertical line in each panel is the pooled effect size in primary analyses using all studies and adjusting for all available confounders.

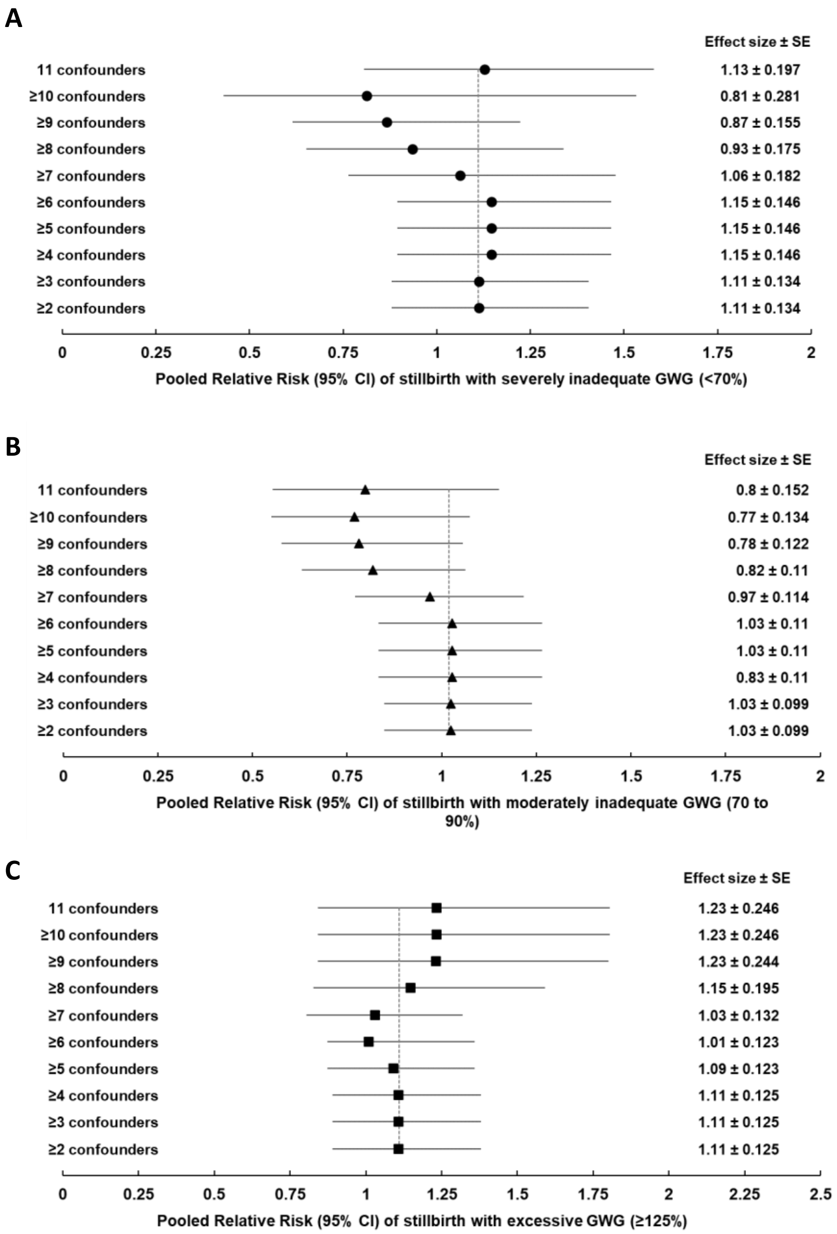

Supplementary Figure 24: Pooled relative risk (RR) and 95% CI of the associations between severely inadequate gestational weight gain (GWG) (A), moderately inadequate GWG (B), and excessive GWG (C) compared to adequate GWG (90-<125%) with stillbirth using two-stage meta-analysis when studies with fewer confounders available for adjustment are included sequentially. The dashed vertical line in each panel is the pooled effect size in primary analyses using all studies and adjusting for all available confounders.

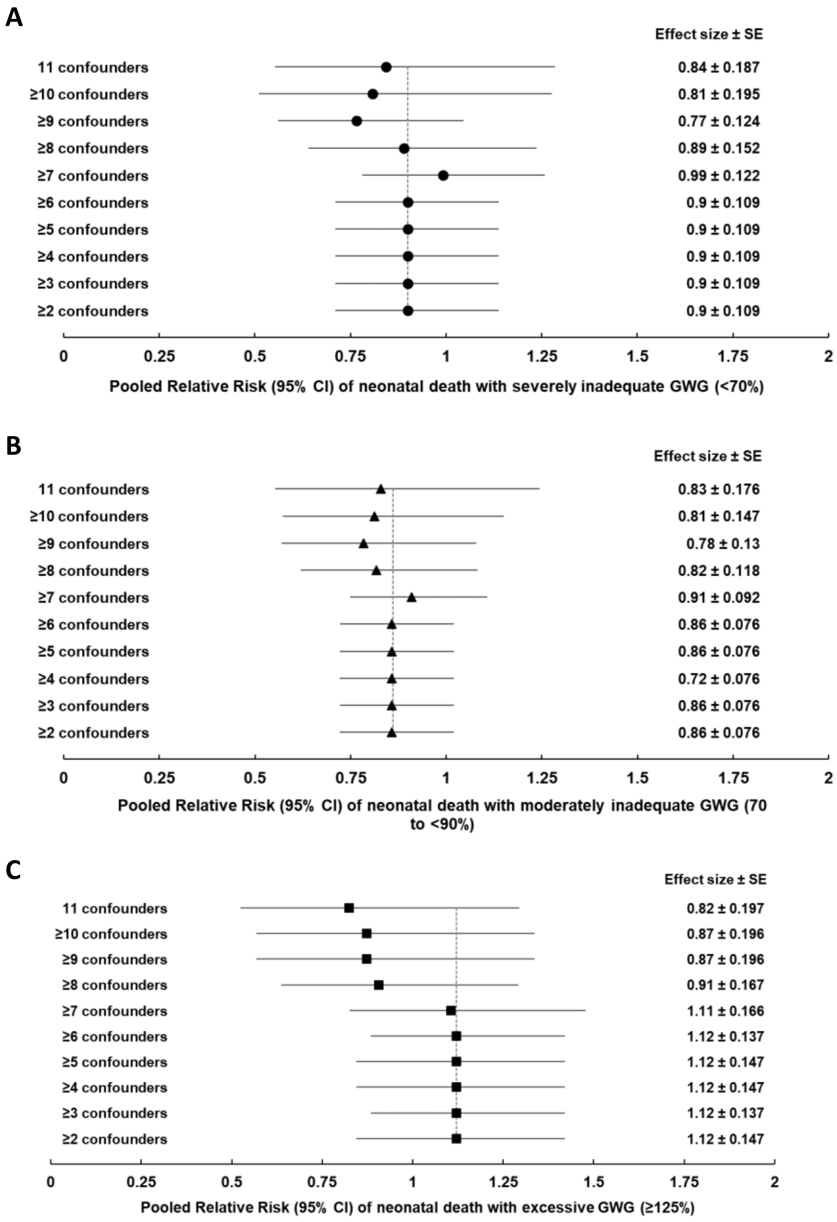

Supplementary Figure 25: Pooled relative risk (RR) and 95% CI of the associations between severely inadequate gestational weight gain (GWG) (A), moderately inadequate GWG (B), and excessive GWG (C) compared to adequate GWG (90-<125%) with neonatal death using two-stage meta-analysis when studies with fewer confounders available for adjustment are included sequentially. The dashed vertical line in each panel is the pooled effect size in primary analyses using all studies and adjusting for all available confounders.

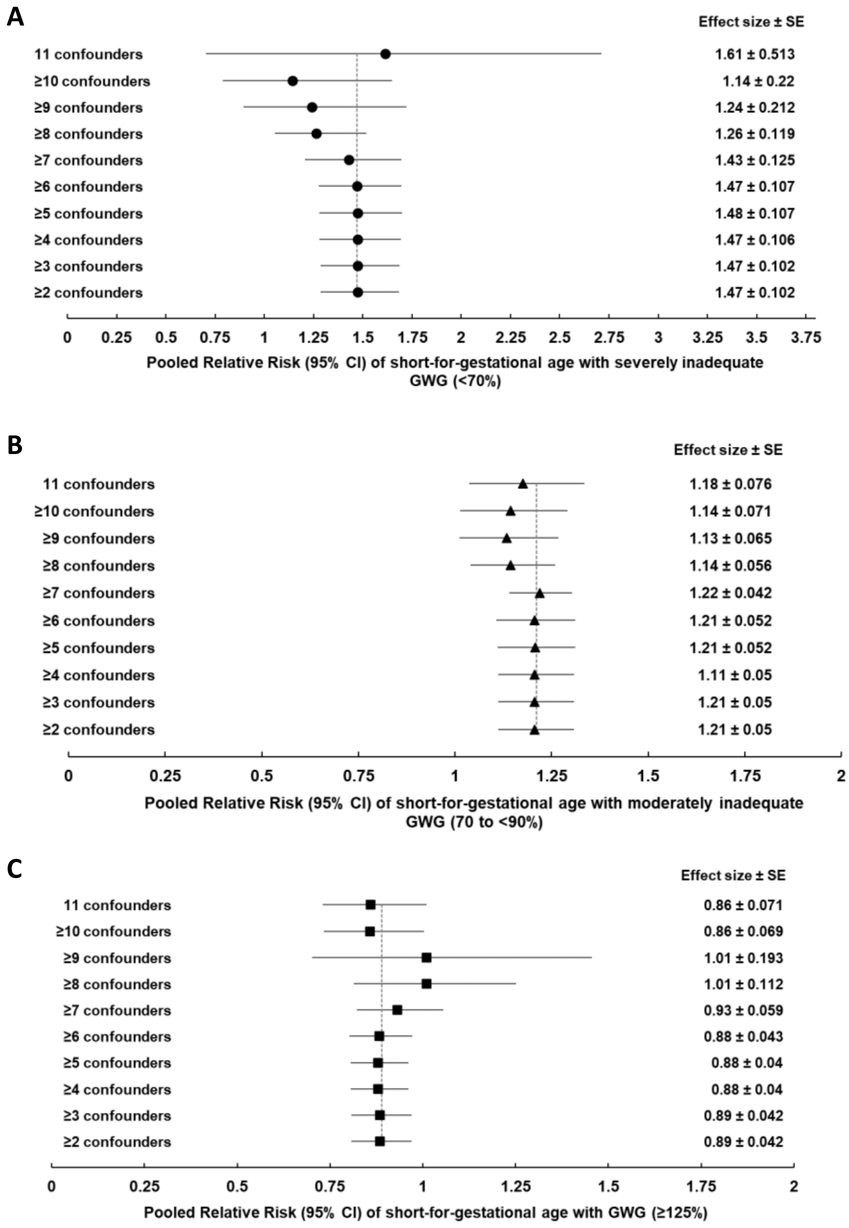

Supplementary Figure 26: Pooled relative risk (RR) and 95% CI of the associations between severely inadequate gestational weight gain (GWG) (A), moderately inadequate GWG (B), and excessive GWG (C) compared to adequate GWG (90-<125%) with **short-for-gestational age** using two-stage meta-analysis when studies with fewer confounders available for adjustment are included sequentially. The dashed vertical line in each panel is the pooled effect size in primary analyses using all studies and adjusting for all available confounders.

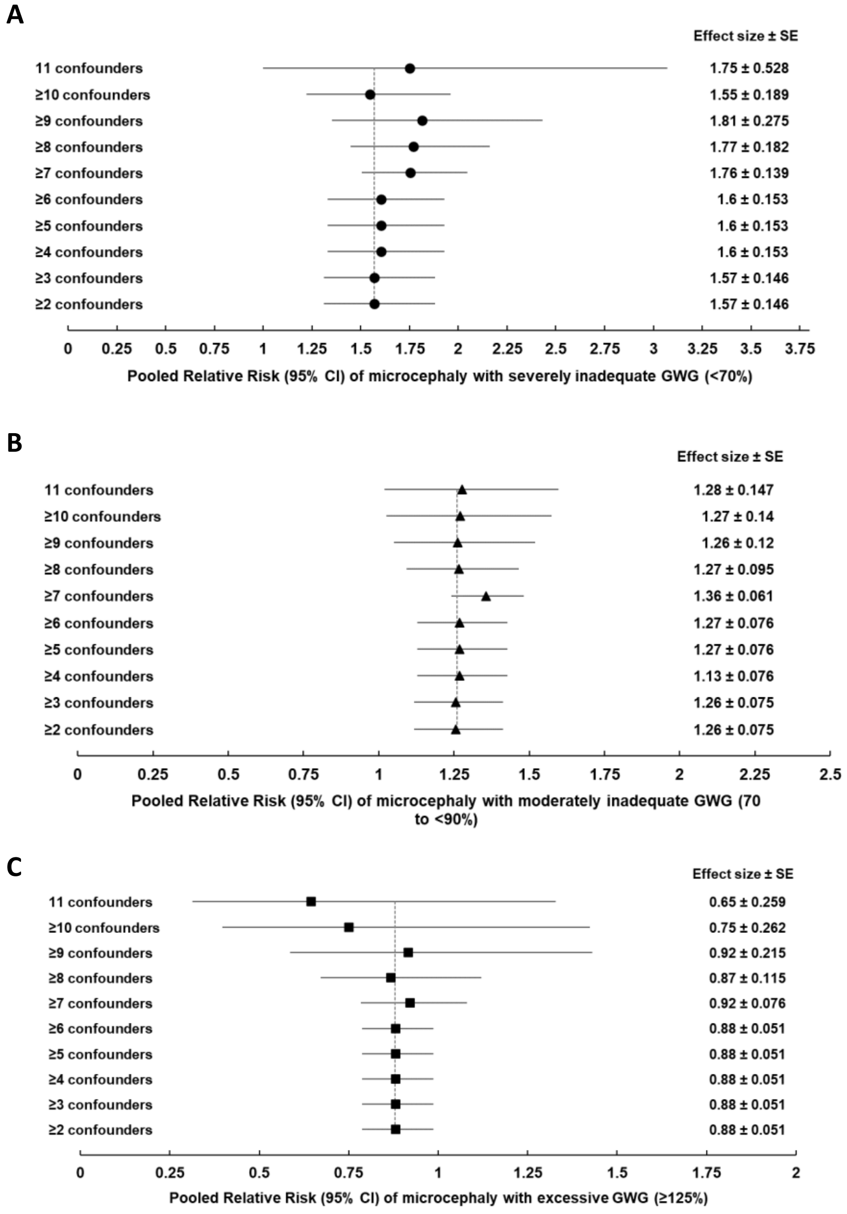

Supplementary Figure 27: Pooled relative risk (RR) and 95% CI of the associations between severely inadequate gestational weight gain (GWG) (A), moderately inadequate GWG (B), and excessive GWG (C) compared to adequate GWG (90-<125%) with **microcephaly** using two-stage meta-analysis when studies with fewer confounders available for adjustment are included sequentially. The dashed vertical line in each panel is the pooled effect size in primary analyses using all studies and adjusting for all available confounders.

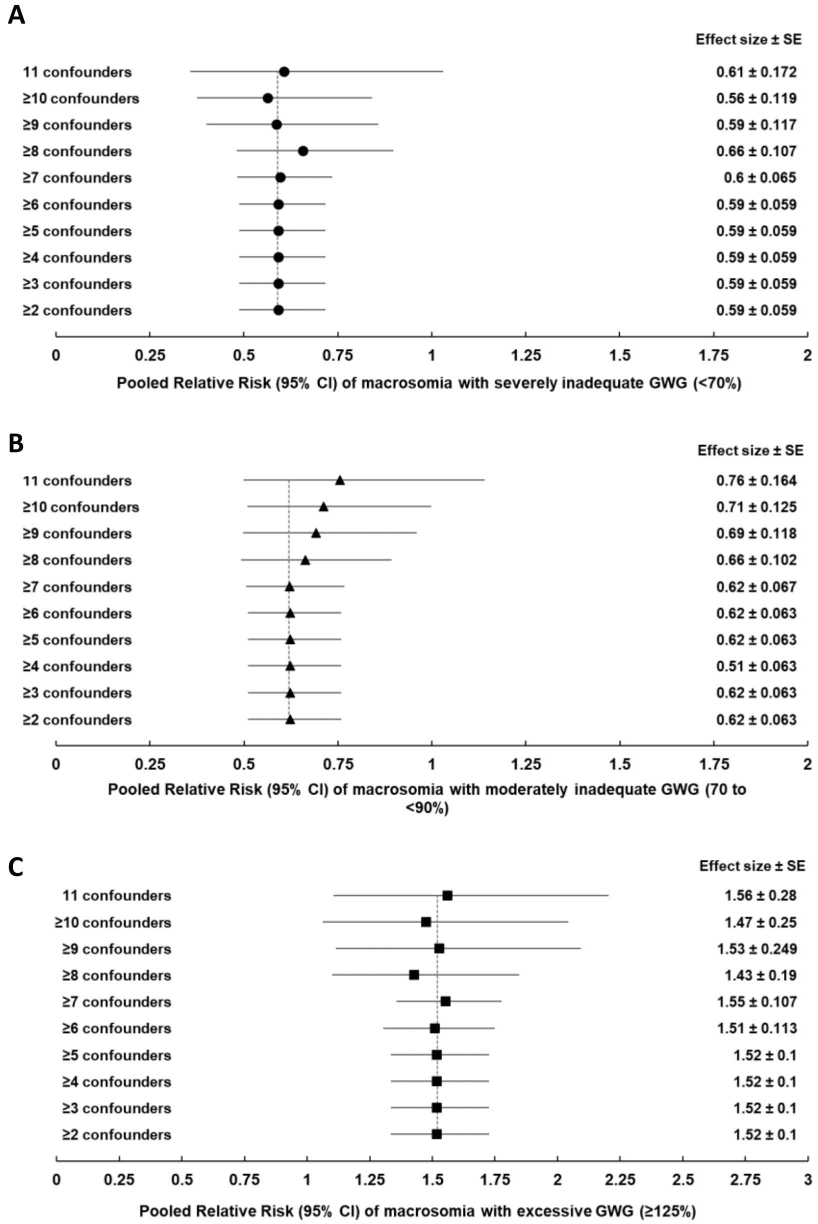

Supplementary Figure 28: Pooled relative risk (RR) and 95% CI of the associations between severely inadequate gestational weight gain (GWG) (A), moderately inadequate GWG (B), and excessive GWG (C) compared to adequate GWG (90-<125%) with **macrosomia** using two-stage meta-analysis when studies with fewer confounders available for adjustment are included sequentially. The dashed vertical line in each panel is the pooled effect size in primary analyses using all studies and adjusting for all available confounders.
