# Supplementary material for: Suboptimal gestational weight gain and neonatal outcomes in low and middle income countries: individual participant data meta-analysis
Source: BMJ. 2023 Sep 21;382:e072249. doi: 10.1136/bmj-2022-072249 (PMC10512803; doi:10.1136/bmj-2022-072249)
Supplement: Supplementary file 2 — Web appendix: Members of consortium [file pern072249.ww2.pdf]

Members of the GWG Pooling Project Consortium
(Each consortium member is a full author)

1. Ajibola Ibraheem Abioye, Department of Global Health and Population, Harvard T.H. Chan School of Public Health, Boston, MA, USA
2. Manfred Accrombessi, Faculty of Infectious and Tropical Diseases, Disease Control Department, London School of Hygiene and Tropical Medicine, WC1E 7HT London, United Kingdom.
3. Seth Adu-Afarwuah, Department of Nutrition and Food Science, University of Ghana, Legon, Ghana
4. Joao Guilherme Alves, Instituto de Medicina Integral Prof. Fernando Figueira (IMIP)
5. Carla Adriane Leal de Araújo, Instituto de Medicina Integral Prof. Fernando Figueira (IMIP)
6. Shams Arifeen, Maternal and Child Health Division, International Centre for Diarrhoeal Disease Research, Bangladesh (icddr,b)
7. Rinaldo Artes, Instituto de Ensino e Pesquisa (INSPER). Rua Quatá 300, Sao Paulo, CEP-04546-042, Brazil
8. Per Ashorn, Center for Child, Adolescent and Maternal Health Research, Faculty of Medicine and Health Technology, Tampere University and Tampere University Hospital, Tampere, Finland
9. Ulla Ashorn, Center for Child, Adolescent and Maternal Health Research, Faculty of Medicine and Health Technology, Tampere University and Tampere University Hospital, Tampere, Finland
10. Omolola Olukemi Ayoola, Department of Paediatrics, Royal Preston Hospital, Preston, UK
11. Gabriela Chico-Barba, Nutrition and Bioprogramming Coordination, Instituto Nacional de Perinatología, Montes Urales 800, Lomas de Virreyes, Mexico City C. P. 11000, Mexico
12. Robin Bernstein, Department of Anthropology, University of Colorado, Boulder, Colorado, USA
13. Zulfiqar A. Bhutta, Centre for Global Child Health, The Hospital for Sick Children, Toronto, Canada
14. Valérie Briand, Institut de Recherche Pour le Développement (IRD), University of Bordeaux, Inserm, UMR 1219, 146 rue Léo-Saignat, 33076, Bordeaux Cedex, France.
15. Elvira Beatriz Calvo, Former Head, Department of Nutrition, Mother & Child Health Direction. Ministry of Health, Argentina.
16. Marly Augusto Cardoso, School of Public Health, University of Sao Paulo. Av. Dr. Arnaldo 715, 01246-904, Sao Paulo/SP, Brazil
17. Yue Cheng, Department of Nutrition and Food Safety Research, School of Public Health, Xi’an Jiaotong University Health Science Center, Xi’an, Shaanxi 710061, P.R. China
18. Peter Ellis Clayton, Faculty of Biology, Medicine & Health, University of Manchester, UK
19. Shalean M. Collins, Tulane University School of Public Health and Tropical Medicine, New Orleans, LA 70112
20. John Kennedy Cruickshank, St Thomas' & Guy's Hospitals, King’s College/ King’s Health Partners, London, UK
21. Delanjathan Devakumar, UCL Institute for Global Health, 30 Guilford Street, London WC1N 1EH, UK
22. Christopher P. Duggan, Departments of Nutrition and Global Health and Population, Harvard T.H. Chan School of Public Health, Boston, MA, USA; Division of Gastroenterology, Hepatology and Nutrition, Boston Children’s Hospital, Harvard Medical School, Boston, Massachusetts
23. Pratibha Dwarkanath, Division of Nutrition, St. John's Research Institute, Bangalore, India
24. Frankie J. Fair, College of Health, Wellbeing and Life Sciences, Sheffield Hallam University, UK
25. Henrik Friis, Department of Nutrition, Exercise and Sports, University of Copenhagen, Rolighedsvej 26,1958 Frederiksberg C, Denmark

26. Alison D. Gernand, The Pennsylvania State University, Department of Nutritional Sciences; 110 Chandlee Laboratory, University Park, PA 16802
27. Shibani Ghosh, Friedman School of Nutrition Science and Policy, Tufts University, Boston, MA, USA
28. Exnevia Gomo, Faculty of Medicine and Health Sciences, University of Zimbabwe, Box A178 Avondale, Harare
29. Rebecca Grais, Epicentre, 14-34 Avenue Jean Jaurès 75019 Paris, France
30. Ousmane Guindo, Epicentre Niger, Niamey, Niger
31. Guadalupe Estrada Gutierrez, Research Direction, Instituto Nacional de Perinatologia, Mexico City, Mexico
32. K. Michael Hambidge, University of Colorado School of Medicine; Aurora, Colorado (Deceased)
33. Rezwanul Haque, The JiVitA Maternal & Child Health and Nutrition Research Institute, Rangpur, Bangladesh
34. Lieven Huybregts, Department of Food Technology, Safety and Health, Ghent University, Coupure links 653, 9000 Gent, Belgium; Poverty, Health and Nutrition Division, International Food Policy Research Institute, Washington, DC, USA
35. Romaina Iqbal, Department of Community Health Sciences, Aga Khan University, Karachi, Pakistan
36. Sheila Isanaka, Epicentre, 14-34 Avenue Jean Jaurès 75019 Paris, France; Harvard T.H. Chan School of Public Health, Departments of Nutrition and Global Health and Population Boston, MA
37. Keith P. West, Jr., Center for Human Nutrition, Dept of Int'l Health, Bloomberg School of Public Health, Johns Hopkins University, Baltimore, MD, USA
38. José Roberto da Silva Junior, Instituto de Medicina Integral Prof. Fernando Figueira (IMIP)
39. Maria Ome-Kaius, Papua New Guinea Institute of Medical Research, PO Box 60, Goroka, EHP, Papua New Guinea
40. Joanne Katz, Center for Human Nutrition, Department of International Health, School of Public Health, Johns Hopkins University, Baltimore, MD, USA
41. Subarna Khatry, Nepal Nutrition Intervention Project Sarlahi
42. Patrick Kolsteren, Department of Food Technology, Safety and Health, Ghent University, Coupure links 653, 9000 Gent, Belgium
43. Nancy F. Krebs, University of Colorado School of Medicine, Aurora, Colorado
44. Teija Kulmala, Pihlajalinna Group, Kehräsaari B, FIN-33200 Tampere, Finland
45. Pratap Kumar, Reproductive Medicine and Surgery, Kasturba Medical College, Manipal Academy of Higher Education, Manipal-576104, Karnataka, India
46. Anura V Kurpad, Department of Physiology & Nutrition, St. John's Medical College, Bangalore, India
47. Carl Lachat, Department of Food Technology, Safety and Health, Ghent University, Coupure links 653, 9000 Gent, Belgium
48. Anna Lartey, Department of Nutrition and Food Science, University of Ghana, Legon, Ghana
49. Qian Li, Department of Nutrition and Food Hygiene, Hubei Key Laboratory of Food Nutrition and Safety, MOE Key Laboratory of Environment and Health, School of Public Health, Tongji Medical College, Huazhong University of Science & Technology, Wuhan, China.
50. Jacqueline M. Lauer, Department of Health Sciences, College of Health & Rehabilitation Sciences: Sargent College, Boston University, Boston, MA
51. See Ling Loy, Department of Reproductive Medicine, KK Women's and Children's Hospital, 100 Bukit Timah Road, Singapore 229899, Singapore; Duke-NUS Medical School, 8 College Road, Singapore 169857, Singapore
52. Nur Indrawaty Lipoeto, Department of Nutrition, Andalas University, Padang, Indonesia
53. Laura Beatriz López, University of Buenos Aires. Faculty of Medicine, Nutrition School, Marcelo T de Alvear 2202. 4to Piso. C1121ABG CABA Argentina

54. Abdullah Al Mahmud, Nutrition and Clinical Services Division; icddr,b; 68 Shaheed Tajuddin Ahmed Sarani, Mohakhali, Dhaka 1212, Bangladesh
55. G. Arun Maiya, Department of Physiotherapy, Manipal College of Health Professions, Manipal Academy of Higher Education, Manipal-576104, Karnataka, India
56. Kenneth Maleta, Department of Nutrition and Dietetics, School of Global and Public Health, Kamuzu University of Health Sciences, Blantyre, Malawi, Along Mahatma Gandhi Road, Private Bag 360, Blantyre 3, Malawi
57. Maíra Barreto Malta, School of Public Health, University of Sao Paulo. Av. Dr. Arnaldo 715, 01246-904, Sao Paulo/SP, Brazil
58. Dharma S. Manandhar, Mother and Infant Research Activities (MIRA), G.P.O. Box 921, Kathmandu, Nepal
59. Charles Mangani, School of Public Health and Family Medicine, University of Malawi, College of Medicine, Blantyre, Malawi, 1 Mahatma Gandhi Road, Private Bag 360, Blantyre 3, Malawi
60. Yves Martin-Prevel, MoISA, University of Montpellier, IRD, CIRAD, CIHEAM-IAMM, INRAE, Institut Agro, Montpellier, France
61. Reynaldo Martorell, Hubert Department of Global Health, Rollins School of Public Health, Emory University, Atlanta, GA
62. Susana L. Matias, Department of Nutritional Sciences and Toxicology, University of California, Berkeley
63. Elizabeth M. McClure, RTI International, Durham, NC, USA
64. Joshua D. Miller, Department of Nutrition, University of North Carolina Chapel Hill, Chapel Hill, NC 27516
65. Marhazlina Mohamad, School of Nutrition and Dietetics, Faculty of Health Sciences, Universiti Sultan Zainal Abidin (UniSZA), Gong Badak Campus, 21030 Kuala Nerus, Terengganu, Malaysia
66. Hamid Jan Jan Mohamed, Nutrition and Dietetics Programme, School of Health Sciences, Universiti Sains Malaysia, 16150 Kubang Kerian, Kelantan, Malaysia
67. Sophie Moore, Department of Women and Children's Health, King's College London, St Thomas' Hospital, Westminster Bridge Road, London, SE1 7EH; MRC Unit The Gambia at the London School of Hygiene and Tropical Medicine, Fajara, The Gambia.
68. Paola S. Mosquera, School of Public Health, University of Sao Paulo. Av. Dr. Arnaldo 715, 01246-904, Sao Paulo/SP, Brazil
69. Malay Kanti Mridha, Center for Non-communicable Diseases and Nutrition, BRAC James P Grant School of Public Health, BRAC University, Dhaka, Bangladesh
70. Ferdinand M. Mugusi, Department of Internal Medicine, Muhimbili University of Health and Allied Sciences, Dar es Salaam, Tanzania
71. Cinthya Muñoz-Manrique, Nutrition and Bioprogramming Coordination, Instituto Nacional de Perinatología, Montes Urales 800, Lomas de Virreyes cp11000, Mexico City, Mexico
72. Salifu Nanga, Department of Basic Sciences, School of Basic and Biomedical Sciences, University of Health and Allied Sciences, PMB 31, Ho Ghana
73. Barnabas K. Natamba, Department of Research and Development, Ministry of Science Technology and Innovation, Kampala, Republic of Uganda (Deceased)
74. Minyanga Nkhoma, School of Public Health and Family Medicine, University of Malawi, College of Medicine, Blantyre, Malawi, 1 Mahatma Gandhi Road, Private Bag 360, Blantyre 3, Malawi
75. David Osrin, UCL Institute for Global Health, 30 Guilford Street, London WC1N 1EH, UK
76. Andrea B. Pembe, Muhimbili University of Health and Allied Sciences, Dar es Salaam, Tanzania
77. Otilia Perichart-Perera, Nutrition and Bioprogramming Coordination, Instituto Nacional de Perinatología, Montes Urales 800, Lomas de Virreyes cp11000, Mexico City, Mexico
78. Ahmed Tijani Bawah, Department of Medical Laboratory Sciences, School of Allied Health Sciences, University of Health and Allied Sciences, PMB 31, Ho Ghana

79. Eric Kwasi Ofori, School of Allied Health Sciences, University of Health and Allied Sciences, PMB 31, Ho Ghana
80. Zul Premji, Department of Parasitology/Medical Entomology, School of Public Health and Social Sciences, Muhimbili University of Health and Allied Sciences, Dar es Salaam, Tanzania
81. Andrew M. Prentice, MRC Unit The Gambia at London School of Hygiene & Tropical Medicine, Atlantic Boulevard, Fajara, PO Box 273, Banjul, The Gambia.
82. Juha Pyykkö, Center for Child, Adolescent and Maternal Health Research, Faculty of Medicine and Health Technology, Tampere University, Tampere, Finland
83. Preetha Ramachandra, Department of Physiotherapy, Manipal College of Health Professions, Manipal Academy of Higher Education, Manipal-576104, Karnataka, India
84. Usha Ramakrishnan, Hubert Department of Global Health, Rollins School of Public Health, Emory University, Atlanta, GA
85. Juan Rivera, National Institute of Public Health, Cuernavaca, Morelos, Mexico
86. Dominique Roberfroid, Faculty of Medicine, University of Namur, Belgium, and Belgian Health Care Knowledge Centre (KCE), Brussels, Belgium
87. Ameyalli Rodríguez-Cano, Nutrition and Bioprogramming Coordination, Instituto Nacional de Perinatología, Montes Urales 800, Lomas de Virreyes cp11000, Mexico City, Mexico
88. Patricia Lima Rodrigues, Instituto de Puericultura e Pediatria Martagão Gesteira, Divisão de Nutrição, Universidade Federal do Rio de Janeiro, Rua Bruno Lobo, 50, Cidade Universitária, Rio de Janeiro – RJ, 21941-912
89. Stephen J Rogerson, Department of Infectious Diseases, Doherty Institute, The University of Melbourne, Melbourne Australia
90. Hugo Martínez-Rojano, Escuela Superior de Medicina del Instituto Politécnico Nacional, Plan de San Luis y Díaz Mirón s/n, Casco de Santo Tomas, Mexico City C. P. 11340, Mexico
91. Patricia H C Rondó, University of Sao Paulo, School of Public Health, Nutrition Department. Avenida Dr Arnaldo 715, Sao Paulo, CEP-05409-010, Brazil
92. Daniel E. Roth, Department of Pediatrics, The Hospital for Sick Children and University of Toronto, 686 Bay Street, Toronto, ON M5G 0A4
93. Reyna Sámano, Nutrition and Bioprogramming Coordination, Instituto Nacional de Perinatología, Montes Urales 800, Lomas de Virreyes, Mexico City C. P. 11000, Mexico
94. Naomi M. Saville, University College London Institute for Global Health, 30 Guilford Street, London, WC1N 1EH
95. Saijuddin Shaikh, Center for Health Research & Development, Society for Applied Studies, 45 Kalu Sarai, New Delhi-110016, India
96. Bhim P. Shrestha, Health Research & Develop Forum (HRDF), Kathmandu, Nepal
97. Robin Shrestha, Friedman School of Nutrition Science and Policy, Tufts University, Boston, MA, USA
98. Hora Soltani, College of Health, Wellbeing and Life Sciences, Sheffield Hallam University, UK
99. Sajid Soofi, Centre of Excellence in Women and Child Health, the Aga Khan University, Karachi, Pakistan
100. Tinku Thomas, Department of Biostatistics, St. John's Research Medical College, Bangalore, India
101. James Tielsch, George Washington Milken Institute School of Public Health
102. Holger W. Unger, Menzies School of Health Research, Charles Darwin University, PO Box 41096, Casuarina NT 0811, Australia
103. Willy Urassa, Department of Microbiology and Immunology, Muhimbili University of Health and Allied Sciences, Dar es Salaam, Tanzania
104. Juliana dos Santos Vaz, Universidade Federal de Pelotas/Faculty of Nutrition; Rua Gomes Carneiro, 1 Pelotas/RS- 96010-610
105. Lee Wu, Center for Human Nutrition, Department of International Health, Bloomberg School of Public Health, Johns Hopkins University, Baltimore, MD, USA

106. Nianhong Yang, Department of Nutrition and Food Hygiene, Hubei Key Laboratory of Food Nutrition and Safety, MOE Key Laboratory of Environment and Health, School of Public Health, Tongji Medical College, Huazhong University of Science & Technology, Wuhan, China.
107. Sera L. Young, Department of Anthropology, Institute for Policy Research, Northwestern University, Evanston, IL 60208
108. Lingxia Zeng, Department of Epidemiology and Biostatistics, School of Public Health, Xi'an Jiaotong University Health Science Center, Xi'an, Shaanxi 710061, P.R. China
109. Chunrong Zhong, Department of Nutrition and Food Hygiene, Hubei Key Laboratory of Food Nutrition and Safety, MOE Key Laboratory of Environment and Health, School of Public Health, Tongji Medical College, Huazhong University of Science & Technology, Wuhan, China.
110. Zhonghai Zhu, Department of Epidemiology and Biostatistics, School of Public Health, Xi'an Jiaotong University Health Science Center, Xi'an, Shaanxi 710061, P.R. China
